# The antifungal properties of terpenoids from the endophytic fungus *Bipolaris* *eleusines*

**DOI:** 10.1007/s13659-023-00407-x

**Published:** 2023-10-23

**Authors:** Yin-Zhong Fan, Chun Tian, Shun-Yao Tong, Qing Liu, Fan Xu, Bao-Bao Shi, Hong-Lian Ai, Ji-Kai Liu

**Affiliations:** School of Pharmaceutical Sciences, South-Central MinZu University, Wuhan, 430074 People’s Republic of China

**Keywords:** Endophytic fungus, *Bipolariseleusines*, Terpenoids, Isolation and structure elucidation, Antifungal activity

## Abstract

A series of terpenoids (**1–17**), comprising six new compounds designated bipolariterpenes A-F (**1–6**) and eleven recognized compounds (**7–17**), were isolated from the wheat culture of the potato endophytic fungus *Bipolaris* *eleusines*. Their structures and stereochemistry were clarified by HRESIMS, NMR, DP4 + probability analyses, and computations for electronic circular dichroism (ECD). All compounds are made up of six meroterpenoids, four sesterterpenes and seven sesquiterpenes. Among them, four sesterterpenes (**4**, **5**, **10**, **11**) were investigated for their antifungal, antibacterial and cytotoxic properties, and six meroterpenoids (**1–3**, **7–9**) were evaluated for their antifungal properties. The compounds **7**, **9**, and **10** had substantial antifungal activity against *Epidermophyton* *floccosum* at a concentration of 100 µM. No antibacterial and cytotoxic activities were observed.

**Graphical Abstract:**

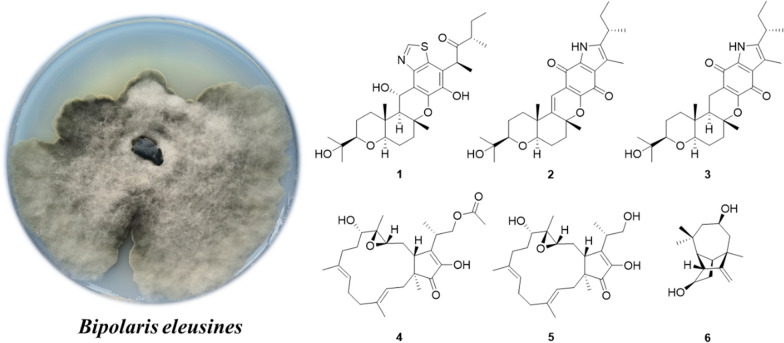

**Supplementary Information:**

The online version contains supplementary material available at 10.1007/s13659-023-00407-x.

## Introduction

Terpenoids, possessed several hundred initial terpene scaffolds, are very important secondary metabolites [1]. Terpenoids have a basic unifying trait that all terpenoids are derived from the five carbon precursor molecules dimethylallyl diphosphate and isopentenyl diphosphate [2]. According to their chemical skeletons, terpenoids are further categorized into monoterpenes, sesquiterpenes, diterpenes, sesterterpenes and triterpenes as well as meroterpenoids derived from the hybridization of polyketide and terpenoid [3]. Terpenoids offer a wide spectrum of biological functions, such as the cancer-fighting monoterpene carvacrol [4], the sesquiterpene atractylenolide V with anti-inflammatory effects [5], and the diterpene 7-deoxynimbidiol with antioxidant activity [6]. One of the most important sources of terpenoids is endophytic fungi, which have been widely reported to have specific biosynthetic capabilities [7, 8]. As a result, the metabolites of endophytic fungi deserve to be further investigated.

**Fig. 1:**
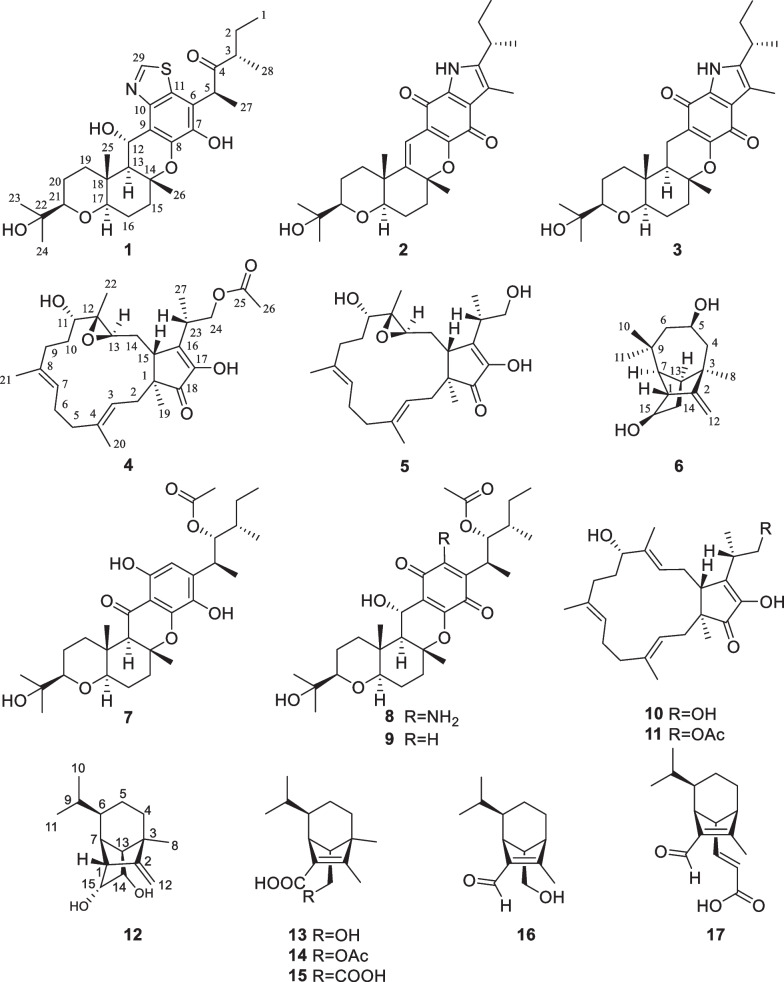
The chemical structures of compounds **1**–**17**

*Bipolaris* is a genus of fungi belonging to the family Pleosporaceae [9, 14], and many metabolites have been reported from several species of this genus, such as terpenes, polyketides, quinones, peptides and alkaloids [10, 11, 14]. The majority of them have a wide spectrum of beneficial biological qualities, such as antifungal, antibacterial, and cytotoxic properties [12–14]. In our previous study on *B.* *eleusines,* a series of meroterpenoids, sesquiterpenes and chromones have been characterized [14–16]. Among them, bipolarithizole A, a new phenylthiazole-sativene merosesquiterpenoid having a MIC value of 16 μg/mL, inhibited *Rhizoctonia* *solani* [14]. To find more potent biological agents, a chemical study on *B.* *eleusines* with a large-scale fermentation was carried out. As a result, seventeen terpenoids, including six undescribed compounds, were isolated from cultures of the fungus *B.* *eleusines*. The details of its isolation, structural elucidation, and antifungal, antibacterial as well as cytotoxic activities are reported in this paper (Fig. 1).

## Results and discussion

**Fig. 2:**
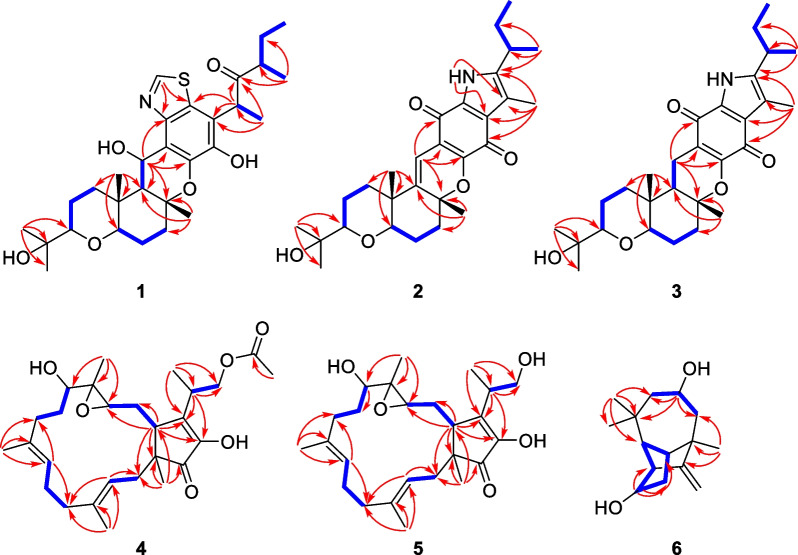
^1^H-^1^H COSY (blue bold lines) and HMBC (red arrows) correlations of compounds **1**–**6**

**Fig. 3:**
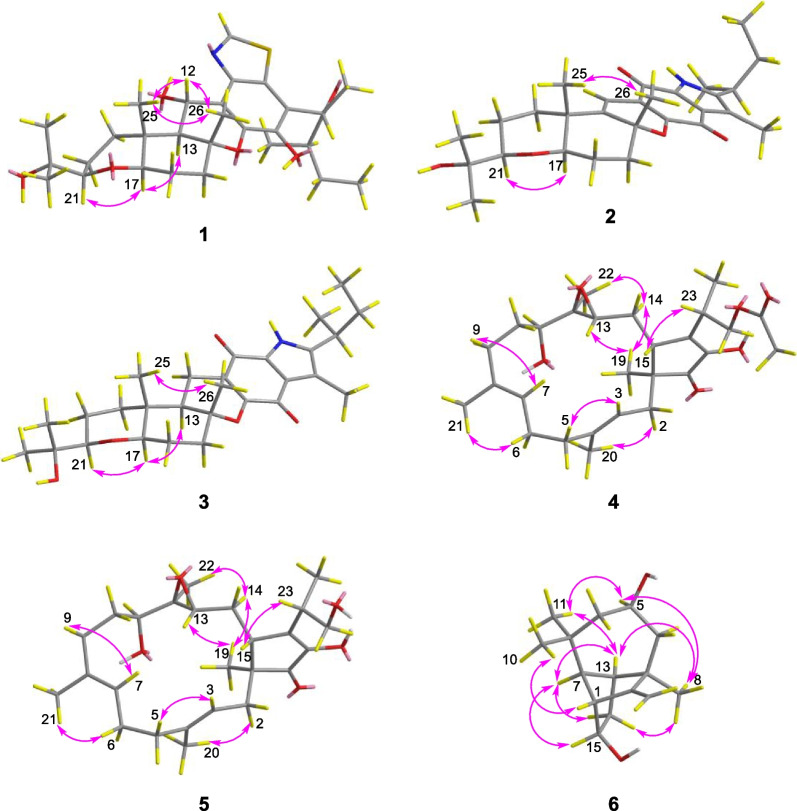
ROESY correlations of compounds **1**–**6**

**Fig. 4:**
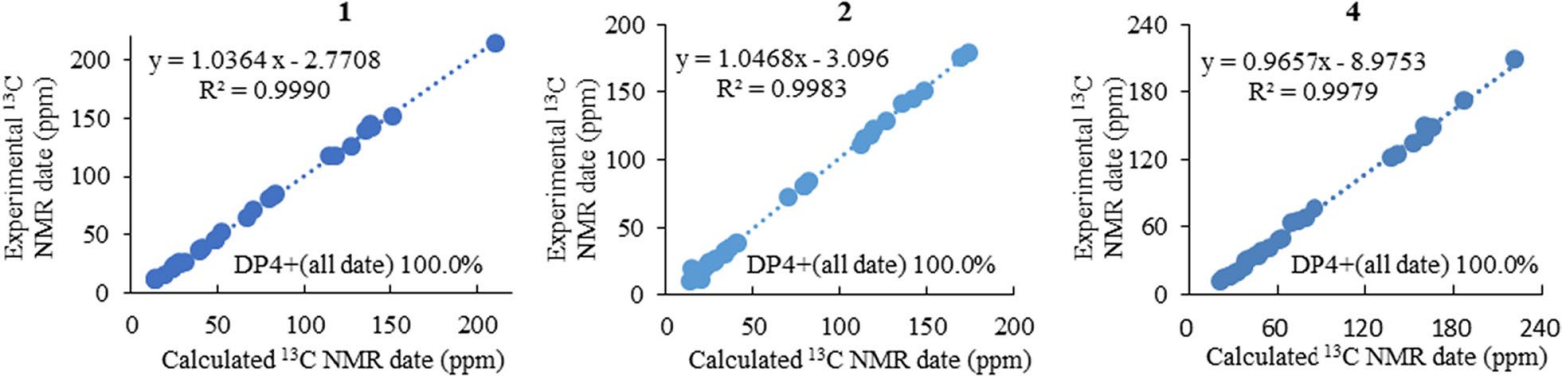
Correlation plots of experimental and calculated ^13^C NMR for **1**, **2**, **4** and DP4 + results of all NMR data

Compound **1** was purified as a colorless solid. The protonated molecule signal in the HRESIMS was used to calculate its chemical formula, which was C_29_H_41_NO_6_S (10 indices of hydrogen deficiency). The ^1^H NMR spectrum of **1** (Table 1) revealed indications for seven methyl groups [*δ*_H_ 0.85 (t, *J* = 7.4 Hz), 1.20 (s), 1.19 (s), 1.11 (s), 1.36 (s), 1.44 (d, *J* = 7.1 Hz) and 0.76 (d, *J* = 6.6 Hz)], two oxymethine protons [*δ*_H_ 3.29 (m), and 3.26 (m)], as well as a number of aliphatic methylene multiplets. In connection with the DEPT experiment, the ^13^C NMR spectrum (Table 1) revealed 29 carbon resonances traceable to seven methyls, five methylenes, seven methines and ten quaternary carbons. The one-dimensional (1D) NMR data of **1** were quite similar to those of known substance 19-dehydroxyl-3-epi-arthripenoid A [17], which is a meroterpenoid with sesquiterpenoid and polyketide components. This was followed by more research into the HMBC and ^1^H − ^1^H COSY spectra, which confirmed the planar structure of **1**. The sesquiterpenoid unit (C-12–C-26) was calculated from the ^1^H–^1^H COSY correlations of H_2_-19/H_2_-20/H-21, H_2_-15/H_2_-16/H-17 and H_2_-12/H-13 as well as HMBC cross-peaks from H_3_-23 to C-21, C-22, and C-24, from H_3_-26 to C-13, C-14, and C-15, and from H_2_-12 to C-13 and C-14 (Fig. 2). The polyketide unit (C-1–C-11, C-27 and C-28) was clarified using the ^1^H–^1^H COSY correlations of H_3_-1/H_2_-2/H-3, as well as HMBC cross-peaks from H_3_-28 to C-2, C-3, and C-4 and from H-27 to C-4, C-5, and C-6 (Fig. 2). Following that, the two units were linked using the HMBC cross-peaks from H_2_-12 to C-8, C-9, C-10, C-13, C-14 and C-18. Furthermore, the ROESY correlations of H-21 (*δ*_H_ 3.29)/H-19*α* (*δ*_H_ 1.49)/H-17 (*δ*_H_ 3.26)/H-13 (*δ*_H_ 2.00) and H-19*β* (*δ*_H_ 2.68)/H_3_-25(*δ*_H_ 1.11)/H_3_-26 (*δ*_H_ 1.36)/H-12 (*δ*_H_ 5.49) proposed that H-21, H-17, and H-13 were *α*-oriented, whereas H_3_-26, H_3_-25 and H-12 were *β*-oriented in the sesquiterpenoid section (Fig. 3). Based on prior discoveries from single crystal X-ray diffraction and the stereoselectivity of enzymes in biosynthesis, the relative configuration of C-5 is known to be fixed (5*S*-configuration) [18, 19]. The absolute configuration of C-3 can be determined using the diagnostic chemical shift of C-2. The ^13^C chemical shift values at the C-2 location are substantially downshifted (*δ*_C_ 26.8 for 3*S* and *δ*_C_ 23.6 for 3*R*), as reported for the 3*S*-conformation [18]. Therefore, the chemical shifts of C-2 at *δ*_C_ 27.3 in **1** revealed that C-3 is *S*-configured. The proposed structure of **1** was further confirmed by the quantum chemical calculations of the NMR data (qccNMR) of two diastereoisomers (3*S*-**1** and 3*R*-**1**). The two epimers were submitted to a thorough conformational screening approach, and the NMR chemical shifts were estimated using the PCM solvent model in methanol at the mPW1PW91/6–31 + G(d,p)//M06-2X/def2-SVP level of theory. As a result, the calculated ^13^C NMR data for 3*S***-1** agreed well with its experimental values and DP4 + analysis also identified 3*S*-**1** as the most instructive structure of **1** with 100% DP4 + chance for all data (Fig. 4 and Additional file 1: Fig. S78). Therefore, compound **1** was defined as (3*S*,5*S*,12*S*,13*S*,14*R*,17*R*,18*R*,21*R*)-**1***.*

**Table 1:** ^1^H (600 MHz) and ^13^C (150 MHz) NMR data of compounds **1–3**

No.	**1**^*b*^		**2**^*a*^		**3**^*b*^	
	*δ*_C_, type	*δ*_H_ (*J* in Hz)	*δ*_C_, type	*δ*_H_ (*J* in Hz)	*δ*_C_, type	*δ*_H_ (*J* in Hz)
1	11.6, CH_3_	0.85, t (7.4)	12.2, CH_3_	0.85, t (7.4)	12.6, CH_3_	0.72, t (7.3)
2	27.3, CH_2_	1.64, m	29.8, CH_2_	1.62, m	30.2, CH_2_	1.54, m
		1.40, m				
3	45.3, CH	2.48, m	32.3, CH	2.84, m	33.7, CH	2.67, m
4	213.4, C		141.6, C		140.2, C	
5	45.6, CH	4.50, q (7.0)	118.5, C		120.2, C	
6	117.5, C		122.5, C		120.0, C	
7	142.3, C		128.8, C		177.5, C	
8	139.7, C		151.0, C		159.2, C	
9	118.3, C		116.3, C		108.2, C	
10	144.8, C		175.3, C		182.9, C	
11	125.6, C		178.9, C		132.7, C	
12	65.3, CH	5.49, d (10.6)	111.1, CH	6.38, s	17.1, CH_2_	2.35, dd (16.8, 4.5)
						1.96, dd (16.7, 13.0)
13	52.5, CH	2.00, d (10.6)	145.2, C		48.5, CH	1.49, dd (13.0, 4.2)
14	81.4, C		81.2, C		83.1, C	
15	38.3, CH_2_	2.04, m	38.0, CH_2_	2.31, m	38.4, CH_2_	2.11, m
		1.99, m		2.05, m		1.81, m
16	25.5, CH_2_	1.83, m	24.7, CH_2_	1.80, m	26.3, CH_2_	1.70, m
		1.64, m		1.69, m		1.56, m
17	84.2, CH	3.26, m	81.3, CH	3.21, m	85.4, CH	3.09, dd (11.7, 3.7)
18	36.9, C		38.5, C		36.8, C	
19	38.8, CH_2_	2.68, m	35.0, CH_2_	2.13, m	37.9, CH_2_	1.79, m
		1.49, m		1.60, m		1.19, m
20	21.8, CH_2_	1.69, m	21.8, CH_2_	1.71, m	22.3, CH_2_	1.58, m
		1.47, m		1.58, m		1.44, m
21	85.3, CH	3.29, m	84.7, CH	3.19, m	86.4, CH	3.13, dd (11.9, 2.9)
22	72.1, C		72.0, C		72.9, C	
23	26.2, CH_3_	1.20, s	26.2, CH_3_	1.20, s	25.6, CH_3_	1.07, s
24	23.8, CH_3_	1.19, s	24.0, CH_3_	1.19, s	25.6, CH_3_	1.09, s
25	12.6, CH_3_	1.11, s	20.4, CH_3_	1.12, s	12.7, CH_3_	0.84, s
26	22.1, CH_3_	1.36, s	26.9, CH_3_	1.53, s	21.4, CH_3_	1.30, s
27	13.2, CH_3_	1.44, d (7.1)	9.9, CH_3_	2.25, s	10.2, CH_3_	2.07, s
28	15.6, CH_3_	0.76, d (6.6)	19.8, CH_3_	1.27, d (7.0)	20.2, CH_3_	1.15, d (6.6)
29	152.2, CH	8.82, s				
NH				9.56, s		

^*a*^Measured in CD_3_OD, ^*b*^Measured in CDCl_3_

Compound **2** was purified as a purple solid. The protonated molecule signal in the HRESIMS was used to calculate its chemical formula, which was C_28_H_37_NO_5_ (11 indices of hydrogen deficiency). The ^1^H NMR spectrum of **2** (Table 1) revealed indications for seven methyl groups [*δ*_H_ 0.85 (t, *J* = 7.4 Hz), 1.20 (s), 1.19 (s), 1.12 (s), 1.53 (s), 2.25 (s) and 1.27 (d, *J* = 7.0 Hz)], two oxymethine protons [*δ*_H_ 3.21 (m) and 3.19 (m)], as well as a number of aliphatic methylene multiplets. In connection with the DEPT experiment, the ^13^C NMR spectrum (Table 1) revealed 28 carbon resonances traceable to seven methyls, five methylenes, four methines (two oxygenated), and twelve quaternary carbons (three oxygenated carbons and two carbonyls). The ^1^H and ^13^C NMR data of **2** were structurally similar to those of cochlioquinone G [17] except for the absence of a hydroxyl group at C-12 and the presence of an additional double bond at *δ*_C_ 111.1 (C-12) and 145.2 (C-13). This hypothesiswas validated by HMBC correlations from H-12 (*δ*_H_ 6.38) to C-10 (*δ*_C_ 175.3), C-9 (*δ*_C_ 116.3), C-8 (*δ*_C_ 151.0) and C-14 (*δ*_C_ 81.2) (Fig. 2). The ROESY correlations of H-21/H-17 indicated that H-21 and H-17 were on the same face and were given as *α*-orientation (Fig. 3). The correlations of CH_3_-25 and CH_3_-26 established the *β*-orientation of H_3_-25 and H_3_-26, which was similar to those of cochlioquinone G. Similar to **1**, the absolute configuration of C-3 was calculated utilizing chemical shifts of C-2 (*δ*_C_ 29.8) and the quantum chemical calculations of the NMR data as well as DP4 + analysis (Fig. 4 and Additional file 1: Fig. S79). Therefore, the absolute configuration of **2** was defined as (3*S*,14*R*,17*R*,18*R*,21*R*)-**2***.*

Compound **3** was purified as a yellow oil. The protonated molecule signal in the HRESIMS was used to calculate its chemical formula, which was C_28_H_39_NO_5_ (10 indices of hydrogen deficiency). The NMR data (Table 1) showed that compound **3** was comparable to compound **2** except for the lack of a double bond group at C-13 (*δ*_C_ 145.2) and C-12 (*δ*_C_ 111.1) and the presence of two alkyl carbons of C-13 (*δ*_C_ 48.5) and C-12 (*δ*_C_ 17.1). This difference indicated that the double bond of C-13 and C-12 were replaced by a single bond, which was confirmed by HMBC correlations from H-12 (*δ*_H_ 2.35) to C-10 (*δ*_C_ 182.9), C-8 (*δ*_C_ 159.2) and C-14 (*δ*_C_ 83.1) and ^1^H–^1^H COSY correlations of H-13/H-12 (Fig. 2). Based on the ROESY profile (Fig. 3) and its likely biosynthesis origin from **2**, the conformation of **3** was determined as the 3*S*,13*R*,14*R*,17*R*,18*R*,21*R* conformation, which should be the same as **2**.

Compound **4** was purified as a colorless powder. The protonated molecule signal in the HRESIMS was used to calculate its chemical formula, which was C_27_H_40_O_6_ (8 indices of hydrogen deficiency). Its ^13^C NMR spectra indicated 27 signals generated by six methyl carbons, seven *sp*^3^ methylene carbons, four *sp*^3^ tertiary carbons, two *sp*^2^ methine carbons, two *sp*^3^ quaternary carbons, four *sp*^2^ nonprotonated carbons and two carbonyl carbons. The ^1^H and ^13^C NMR data of **4** (Table 2) were similar to those of terpestacin (**11**) [20]. The obvious differences disclosed were that the double bond between C-12 and C-13 was absent and two more oxygenated resonances (*δ*_C_ 65.1 and 63.5) occurred, suggesting that the double bond in **11** is oxidized to form an epoxy ring in compound **4**, which was confirmed by the ^1^H–^1^H COSY correlations of H-15/H-14/H-13 and HMBC correlations from H-13 to C-22, C-15 and C-11 (Fig. 2). ROESY correlations between H-3/H-5, H-20/H-2, H-7/H-9 and H-21/H-6 show that the two double bonds in **4** were *E*-configured. While the ROESY correlation between H-15/H-23 implied that they were on the identical side and assigned as *β*-orientation, the correlation between H-19/H-13, H-19/H-14b, H-14b/H-22, suggested that they were assigned as *α*-orientation (Fig. 3). Furthermore, quantum chemical calculations of the nuclear magnetic resonance data (qccNMR) of two diastereoisomers, (12*S*,13*S*)-**4** and (12*R*,13*R*)-**4**, were undertaken to identify the configuration of the epoxy ring on C-12 and C-13. The two epimers were rigorously conformationally screened, and NMR chemical shifts were estimated in methanol using the mPW1PW91/6–31 + G(d,p)//M06-2X/def2-SVP level of theory and the PCM solvent model. With a DP4 + probability of 100%, the DP4 + analysis also revealed (12*R*,13*R*)-**4** as the most plausible structure for **4**. (all data) (Fig. 4), which was compatible with the ROESY study results presented above. Therefore, the absolute configuration of **4** was specified as (1*S*,11*S*,12*R*,13*R*,15*R*,23*S*)-**4**.

**Table 2:** ^1^H (600 MHz) and ^13^C (150 MHz) NMR data of compounds **4–6**

No.	**4**^*a*^		**5**^*b*^		**6**^*a*^	
	*δ*_C_, type	*δ*_H_ (*J* in Hz)	*δ*_C_, type	*δ*_H_ (*J* in Hz)	*δ*_C_, type	*δ*_H_ (*J* in Hz)
1	50.0, C		49.0, C		58.5, CH	2.60, m
2	39.2, CH_2_	2.36, m	37.9, CH_2_	2.40, m	164.4, C	
		1.80, m		1.86, m		
3	122.3, CH	5.37, t (8.4)	120.7, CH	5.29, t (8.2)	42.2, C	
4	139.8, C		138.9, C		53.0, CH_2_	2.07, m
						1.65, m
5	41.0, CH_2_	2.25, m	39.9, CH_2_	2.21, m	67.2, CH	3.87, m
		2.07, m		2.05, m		
6	24.8, CH_2_	2.28, m	23.8, CH_2_	2.21, m	47.1, CH_2_	1.98, m
		2.21, m				1.21, m
7	124.7, CH	5.11, t (6.0)	123.8, CH	5.03, t (6.5)	58.8, CH	1.81, m
8	134.9, C		133.8, C		29.7, CH_3_	0.96, s
9	34.8, CH_2_	2.19, m	34.1, CH_2_	2.21, m	32.5, C	
		1.99, m		1.94, m		
10	31.6, CH_2_	1.76, m	29.7, CH_2_	1.85, m	31.6, CH_3_	0.95, s
		1.65, m		1.62, m		
11	76.6, CH	3.05, t (7.0)	75.9, CH	3.11, dd (8.2, 6.1)	30.4, CH_3_	1.10, s
12	65.1, C		64.6, C		103.7, CH_2_	4.96, s
						4.74, s
13	63.5, CH	2.86, dd (7.4, 2.2)	63.8, CH	2.92, dd (7.5, 2.3)	46.2, CH	2.09, m
14	29.6, CH_2_	1.88, m	28.0, CH_2_	1.88, m	38.7, CH_2_	2.21, m
		1.51, m		1.51, m		1.19, m
15	49.1, CH	2.79, dd (8.1, 4.2)	48.3, CH	2.81, dd (6.9, 4.7)	75.0, CH	3.66, m
16	149.4, C		149.2, C			
17	149.6, C		146.8, C			
18	209.0, C		207.3, C			
19	18.6, CH_3_	1.03, s	18.5, CH_3_	1.05, s		
20	15.8, CH_3_	1.68, s	15.9, CH_3_	1.65, s		
21	16.4, CH_3_	1.63, s	16.1, CH_3_	1.61, s		
22	11.0, CH_3_	1.24, s	10.9, CH_3_	1.27, s		
23	34.8, CH	2.90, m	36.7, CH	2.78, m		
24	67.5, CH_2_	4.29, m	66.3, CH_2_	3.87, m		
				3.80, m		
25	172.8, C		14.5, CH_3_	1.27, d (1.3)		
26	20.8, CH_3_	2.00, s				
27	14.8, CH_3_	1.28, d (7.0)				

^*a*^Measured in CD_3_OD, ^*b*^Measured in CDCl_3_

Compound **5** was purified as a colorless powder. The protonated molecule signal in the HRESIMS was used to calculate its chemical formula, which was C_25_H_38_O_5_ (7 indices of hydrogen deficiency). Its ^13^C NMR spectra revealed 25 signals from five methyl carbons, seven *sp*^3^ methylene carbons, four *sp*^3^ tertiary carbons, two *sp*^2^ methine carbons, two *sp*^3^ quaternary carbons, four *sp*^2^ nonprotonated carbons and a carbonyl carbon. The NMR spectra of **5** indicated that it is closely linked to **4**, except that **4** lacks an acetyl group and has an additional hydroxyl group at C-24, which is corroborated by the 42 Da drop in molecular weight of **5** when compared to **4**. Based on ROESY profile (Fig. 3) and its potential biosynthetic origin from **4**, the configuration of **5** would be equivalent to **4** for (1*S*,11*S*,12*R*,13*R*,15*R*,23*S*)-**5**.

**Fig. 5:**
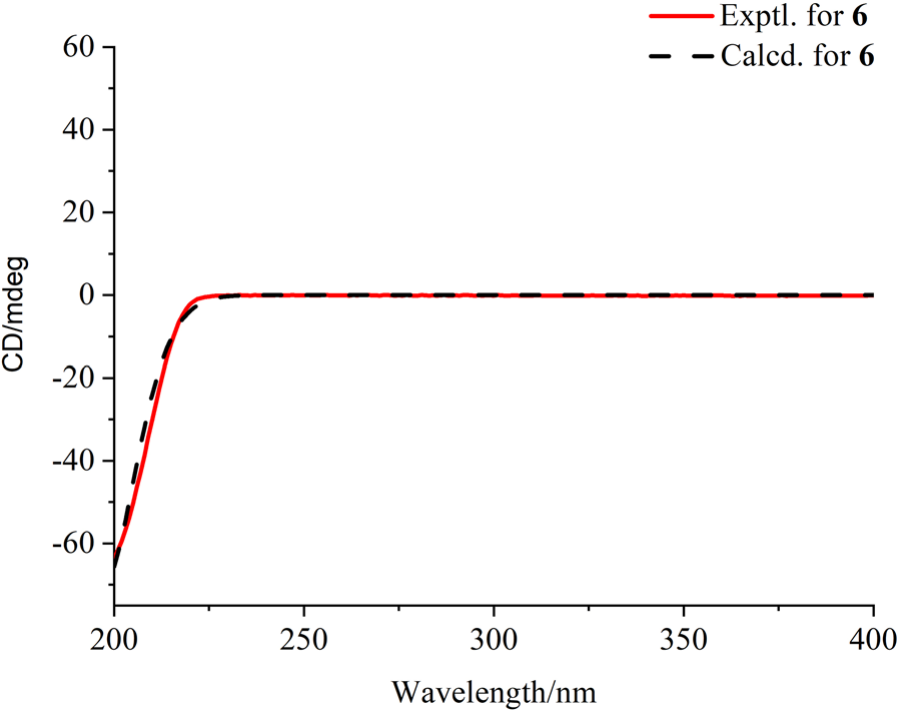
Experimental and calculated ECD curves of compound **6**

Compound **6** was purified as a colorless powder. The protonated molecule signal in the HRESIMS was used to calculate its chemical formula, which was C_15_H_24_O_2_ (4 indices of hydrogen deficiency). The ^1^H and ^13^C NMR spectra (Table 2) revealed 15 signals from three methyl carbons, three *sp*^3^ methylene carbons, one *sp*^2^ methylene carbon, five *sp*^3^ tertiary carbon, two *sp*^3^ quaternary carbons, and one *sp*^2^ nonprotonated carbon. According to the preceding data, the structural properties of **6** were comparable to those of longi-*β-*nozigiku alcohol [21], with the exception of an extra hydroxyl group in **6**. Chemical shifts at the C-5 (*δ*_C_ 67.2) level have increased relative, together with the critical HMBC correlation from H-5 (*δ*_H_ 3.87) to C-3 and C-9, suggesting the hydroxyl group was annexed to C-5 (Fig. 2). The ROESY correlations of H-15/H-7and H-13/H-7 indicated that H-15, H-13 and H-7 are on the identical side and belong to the *α*-orientation (Fig. 3). The correlations of H-1/H-10 determined the *β*-orientation of H-1. Meanwhile, the correlations of H-5/H-11, H-11/H-13 and H-8/H-5 indicated that H-13, H-11, H-8 and H-5 are on the identical side and belong to the *α*-orientation (Fig. 3). Hence, the hydroxyl groups that exist at C-5 is *β***-**configuration. The absolute configurations of **6** was determined by comparing the experimental ECD spectra with calculated ECD spectra (Fig. 5). Therefore, the absolute configuration of **6** was defined as (1*R*,3*R*,5*R*,7*S*,13*S*,15*R*)*-***6**.

Eleven known compounds were identified as isocochlioquinone A (**7**) [11], cochlioquinone N (**8**) [22], stemphone (**9**) [23], (–)-terpestacin (**10**) [24], fusaproliferin (**11**) [20], *cis*-sativenediol (**12**) [25], helminthosporic acid (**13**), cochliobolin B (**14**) and cochliobolin A (**15**) [26], helminthosporol (**16**) [27] and bipolarisorokin H (**17**) [28] respectively, based on spectroscopic data comparisons with reported values.

Since terpenoids are known to exhibit a variety of biological activities, the four sesterterpenes (**4**, **5**, **10** and **11**) were tested for their antibacterial, antifungal and cytotoxic properties, and six meroterpenoids (**1–3**, **7–9**) were evaluated for their antifungal activities. The antibacterial activity was evaluated against the *Escherichia* *coli*, *Staphylococcus* *aureus* subsp. Aureus, *Salmonella* *enterica* subsp. Enterica and *Pseudomonas* *aeruginosa* [29], but all tested compounds did not show any promising activity up to the highest tested concentration of 100 µM (Additional file 1: Table S7). Likewise, no cytotoxic activity against the human cancer cell lines (HL-60, A549, SMMC-7721, MDA-MB-231 and SW480) could be detected (at 40 μM) (Additional file 1: Table S8). The antifungal activity was evaluated against the *Epidermophyton* *floccosum*, *Trichophyton* *rubrum*, *Microsporum* *gypseum* and *Candida* *albicans.* The compounds **7**, **9**, and **10** had good antifungal activity against *E.* *floccosum* at a concentration of 100 µM, and compounds **1**, **4**, **5** and **10** exhibited moderate or weak antifungal activity against *M.* *gypseum* (Table 3)*.*

**Table 3:** Inhibitory effect of compounds on four strains of fungi

Compound	Density (µM)	Inhibition rate (%)			
		*C.* *albicans*	*E.* *floccosum*	*T.* *rubrum*	*M.* *gypseum*
Amphotericin B	0.5	100.092 ± 0.054			
Terbinafine hydrochloride	3		102.044 ± 0.354		101.872 ± 0
	15			101.804 ± 0.773	
**1**	100	− 1.701 ± 0.586	65.06 ± 0	45.771 ± 0.547	51.07 ± 5.954
**2**	100	6.326 ± 0.236	42.382 ± 0.512	5.128 ± 0.241	− 16.246 ± 1.052
**3**	100	− 18.526 ± 0.456	46.977 ± 2.168	− 21.855 ± 3.279	− 20.503 ± 3.681
**4**	100	12.075 ± 3.379	− 1.514 ± 2.471	− 59.348 ± 2.767	53.108 ± 0.354
**5**	100	6.969 ± 0.265	8.094 ± 0.412	− 95.87 ± 0.923	55.981 ± 1.237
**7**	100	− 8.244 ± 0.738	**81.611 ± 2.601**	− 3.693 ± 2.733	24.967 ± 8.383
**8**	100	− 7.815 ± 0.786	18.78 ± 0.434	− 0.215 ± 3.279	18.418 ± 24.981
**9**	100	− 5.977 ± 2.731	**94.484 ± 0.867**	61.228 ± 0.547	38.439 ± 20.418
**10**	100	12.309 ± 0.133	**99.806 ± 1.165**	− 46.957 ± 0	60.354 ± 1.767
**11**	100	− 3.104 ± 2.187	− 3.844 ± 1.647	19.566 ± 1.845	37.741 ± 0.177

## Conclusions

In conclusion, seventeen terpenoids, including six undescribed compounds, were identified from the endophytic fungus *B.* *eleusines*. Among these isolates, no compounds exhibited antibacterial and cytotoxic activities. However, the compounds **7**, **9** and **10** had significant antifungal activity against *E.* *floccosum*. Our initial findings indicate that the endophytic fungus *B.* *eleusines* is a plentiful supply of different and bioactive terpenoids.

### General experimental procedures

A Bruker spectrometer (Bruker, Germany, model AM600) was used to obtain 1D and 2D spectra. A Q Exactive HF mass spectrometer (Thermo Fisher Scientific, USA) was used to collect HRESIMS date. Applied Circular dichroism spectrometer (Chirascan, New Haven, USA) was used to record CD spectra. Column chromatography (CC) was performed on silica gel (Qingdao Marine Chemical Ltd., Qingdao, China), RP-18 gel (Fuji Silysia Chemical Ltd., Japan), and Sephadex LH-20 (Pharmacia Fine Chemical Co., Ltd., Sweden) [30]. Semipreparative HPLC experiments were carried out on an Agilent 1260 HPLC with an Agilent Zorbax SB-C_18_ column (5 μm, 250 × 9.8 mm). Thin layer chromatography (GF 254) was used to monitor fractions, and spots were seen by heating silica gel plates coated with vanillin and 10% H_2_SO_4_ in ethanol.

### Culture and fermentation of fungal material

This fungus was identified by PCR amplification using fungal 18S universal primers ITS1 and ITS4. The sequenced 18S rDNA ITS sequences were subjected to homology sequence alignment analysis, which, using BLAST, showed 99% maximum similarity to *Bipolaris* *eleusines* with the gene bank registration number (KY909768.1). Therefore, it was identified as *B.* *eleusines*, which was preserved in South-Central Minzu University, China. The strains were inoculated into PDA agar medium and incubated for 3–5 days at a constant temperature of 25 °C. The medium was then cut into several small pieces and placed into a solid wheat medium (50 g wheat and 50 mL water in 500 mL culture flasks, autoclaved at 120 °C for 30 min, for a total of 212 flasks) and fermented at 25 °C for 28 days.

### Extraction and isolation

The wheat medium was pounded and extracted five times with EtOAc to yield 167 g of raw extract. The EtOAc extract was subjected to silica gel CC and eluted with a gradient of CH_2_Cl_2_–MeOH (100:0–0:100, *v/v*) to obtain six fractions (A–F). Fraction D (31.6 g) was subjected to C18 MPLC using MeOH–H_2_O (30:70–100:0, *v/v*), yielding ten subfractions (D1–D10). Fraction D5 (1.9 g) was divided by CC over silica gel and eluted in PE with a gradient of increasing acetone concentrations (30–100) to provide six fractions (D5-1–D5-6). Fraction D5-5 (613.9 mg) was divided by using preparative HPLC (gradient elution with MeOH–H_2_O, 18:82–40:60) to get **11** (3.2 mg, *t*_R_ = 12.7 min) and **12** (2.1 mg, *t*_R_ = 13.5 min). The Fraction D9 (903.5 mg) was separated into six subfractions D9-1–D9-6 by column chromatography (Sephadex LH-20, MeOH). Fraction D9-3 (113.6 mg) was separated by using preparative HPLC with a gradient of MeCN–H_2_O (50:50–60:40, *v/v*) to obtain **8** (4.3 mg, *t*_R_ = 20.5 min), **9** (1.2 mg, *t*_R_ = 24.6 min) and **10** (2.2 mg, *t*_R_ = 25.2 min). Fraction D9-4 (160.1 mg) was separated by using preparative HPLC with a gradient of MeCN–H_2_O (40:60–60:40, *v/v*) to get **7** (3.5 mg, *t*_R_ = 12.1 min) and **16** (2.1 mg, *t*_R_ = 14.5 min). Fraction D9-6 (106.2 mg) was separated by Sephadex LH-20 (MeOH) to give four subfractions D9-6–1–D9-6–4 and then purified by usingpreparative HPLC with MeCN–H_2_O (50:50–60:40, *v/v*) to obtain **2** (12.2 mg, *t*_R_ = 32.6 min), **14** (4.5 mg, *t*_R_ = 34.2 min) and **13** (1.1 mg, *t*_R_ = 35.3 min). Fraction E (33.2 g) was separated by C18 MPLC using MeOH–H_2_O (10:90–100:0, *v/v*), yielding eight subfractions (E1–E8). The fraction E4 (3.4 g) was isolated by using Sephadex LH-20 and eluted with MeOH to yield five subdivisions E4-1–E4-5. Fraction E4-2 (532.6 mg) was divided by C18 MPLC with MeOH–H_2_O (60:40–75:25, *v/v*) to obtain **3** (5.2 mg, *t*_R_ = 18.2 min) and **15** (13.5 mg, *t*_R_ = 24.5 min). Fraction E4-5 (267.9 mg) was separated by using preparative HPLC with MeOH–H_2_O (65:35–80:20, *v/v*) to afford **6** (20.6 mg, *t*_R_ = 22.4 min) and **4** (15.7 mg, *t*_R_ = 26.8 min). Fraction E8 (7.4 g) was subjected to CC over silica gel with a gradient elution of PE-acetone (30:1–30:60, *v/v*) and then separated using a preparative C18 HPLC column with MeCN–H_2_O (45:55–60:40, *v/v*) to afford **1** (4.1 mg, *t*_R_ = 26.4 min), **5** (20.6 mg, *t*_R_ = 34.3 min) and **17** (6.2 mg, *t*_R_ = 36.4 min).

#### *Bipolariterpene A* (1)

Colorless solid; mp 247–252 ℃; UV (CH_3_OH) *λ*_max_ (log *ε*) = 235 (3.61); IR (KBr): 3414, 2970, 2939, 2874, 1713, 1655, 1458, 1431, 1381, 1342, 1308, 1246, 1146, 1092, 1026, 833 cm^−1^; ${\left[\boldsymbol{\alpha }\right]}_{\mathrm{D}}^{24}$ +361 (*c* 0.5, CH_3_OH); ^1^H NMR and ^13^C NMR (CDCl_3_) data are shown in Table 1; HRESIMS *m/z* 532.27271 [M + H] ^+^ (calcd for C_29_H_42_NO_6_S, 532.27274).

#### *Bipolariterpene B* (2)

Yellow oil; UV (CH_3_OH) *λ*_max_ (log *ε*) = 210 (2.49), 225 (2.49), 275 (2.57); IR (KBr): 3248, 2963, 2936, 1666, 1632, 1578, 1493, 1443, 1377, 1304, 1269, 1207, 1138, 1096, 1053, 1022, 957 cm^−1^; ${\left[\boldsymbol{\alpha }\right]}_{\mathrm{D}}^{24}$ +315 (*c* 0.5, CH_3_OH); ^1^H NMR and ^13^C NMR (CDCl_3_) data are shown in Table 1; HRESIMS *m/z* 490.25650 [M + Na] ^+^ (calcd for C_28_H_37_NNaO_5_, 490.25639).

#### *Bipolariterpene C* (3)

Purple solid; mp 259–264 ℃; UV (CH_3_OH) *λ*_max_ (log *ε*) = 245 (2.81); IR (KBr): 3453, 2959, 2939, 2874, 2862, 1666, 1647, 1628, 1570, 1516, 1458, 1381, 1334, 1227, 1207, 1142, 1092, 1022 cm − 1; ${[\alpha ]}_{\mathrm{D}}^{25}$ +216 (*c* 0.1, CH_3_OH); ^1^H NMR and ^13^C NMR (methanol-*d*_4_) data are shown in Table 1; HRESIMS *m/z* 470.28995 [M + H] ^+^ (calcd for C_28_H_40_NO_5_, 470.29010).

#### *Bipolariterpene D* (4)

Colorless powder; UV (CH_3_OH) *λ*_max_ (log *ε*) = 205 (2.69), 260 (2.55); IR (KBr): 3387, 2974, 2939, 1740, 1709, 1655, 1458, 1373, 1238, 1038 cm^−1^; ${\left[\boldsymbol{\alpha }\right]}_{\mathrm{D}}^{24}$ +14 (*c* 0.6, CH_3_OH); ^1^H NMR and ^13^C NMR (methanol-*d*_4_) data are shown in Table 2; HRESIMS *m/z* 483.27190 [M + Na]^+^ (calcd for C_27_H_40_NaO_6_, 483.27171).

#### *Bipolariterpene E* (5)

Colorless powder; UV (CH_3_OH) *λ*_max_ (log *ε*) = 265 (1.59); IR (KBr): 3375, 2970, 2936, 1697, 1651, 1450, 1408, 1389, 1312, 1142, 1030, 941, 856 cm^−1^; ${\left[{\varvec{\upalpha}}\right]}_{\mathrm{D}}^{24}$ +19 (*c* 0.5, CH_3_OH); ^1^H NMR and ^13^C NMR (CDCl_3_) data are shown in Table 2; HRESIMS m/z 441.26097 [M + Na]^+^ (calcd for C_25_H_38_NaO_5_, 441.26115).

#### *Bipolariterpene F* (6)

Colorless powder; UV (CH_3_OH) *λ*_max_ (log *ε*) = 210 (2.56); IR (KBr): 3314, 2955, 1659, 1597, 1450, 1346, 1177, 1130, 1042, 1003, 949, 880 cm^−1^; ${\left[\boldsymbol{\alpha }\right]}_{\mathrm{D}}^{20}$ -2 (*c* 0.5, CH_3_OH); ^1^H NMR and ^13^C NMR (methanol-*d*_4_) data are shown in Table 2; HRESIMS *m/z* 237.18486 [M + H]^+^ (calcd for C_15_H_25_O_2_, 237.18491).

### Quantum chemical calculations

#### ^13^C NMR calculation

The NMR chemical shift calculations were carried out in Gaussian 16 using density functional theory (DFT) [31]. Spartan’14 software was used to conduct the preliminary conformational distribution search. GaussView 6.0 was used to view conformational structures and modify computation input files for calculation. At the M062X/def2svp level, all ground-state geometries were optimized, and the stable conformations obtained at that level were then used in magnetic shielding constants at the mpw1pw91/6–31 + g(d,p) level. GaussView 6.0 was used to display the calculated chemical shift of each atom in each conformer, and the final chemical shift of each atom was calculated from the Boltzmann distribution of each conformer. The stereochemistry was assigned using the DP4 + probability analysis of calculated and experimental chemical shifts.

#### ECD calculations

The absolute conformations of the compounds were determined using Gaussian 16 software. Briefly, the relative conformations of the compounds were first determined by ROESY spectroscopy, followed by a preliminary conformational analysis based on Spartan’14software. The obtained conformations were optimized at the M062X/def2svp level of density functional theory (TDDFT), and then ECD calculations were performed by M062X/def2svp. The ECD curves were generated by Origin software.

### Antifungal assay

The endophytic fungus used in this assay was *Bipolaris* *eleusines*, and the samples to be tested were diluted in 96-well plates, and the fungal solution was added to each well. *C.* *albicans* was nurtured at 37 °C for 24 h, and Filamentous fungi were nurtured at 25 °C for 5 days, and the absorbance value at 625 nm was measured by an enzyme marker. The experiment was set up with a medium blank control, a fungal control [32], and positive drug controls for amphotericin B and terbinafine hydrochloride.

### Antibacterial assay

The samples were evaluated for their antibacterial activity against *Escherichia* *coli*, *Staphylococcus* *aureus* subsp. Aureus, *Salmonella* *enterica* subsp. Enterica and *Pseudomonas* *aeruginosa*. Dissolve the compounds to be tested to a concentration range of 100 μM and add it to a 96-well culture plate. Each well received a bacteria solution until the final concentration reached 5 × 10^5^ CFU/ml. It was then cultivated at 27 ℃ for 24 h, and the inhibition rate was determined by the microplate reader at OD 600 nm. The medium blank control was used in the experiment [12]. Sodium penicillin G and ceftazidime were used as the positive control.

### Cytotoxicity assay

Five human cancer cell lines including HL-60 (promyelocytic leukemia), A549 (lung epithelial cancer), SMMC-7721 (liver cancer), MDA-MB-231 (breast cancer), and SW480 (colon cancer) were used for cytotoxicity assays by MTS method [12]. In a word, cells were cultured in DMEM media supplemented with 10% fetal bovine serum before being injected in 96-well culture plates and treated with different concentrations of compounds. After 24 h, each well received 20 μL of MTS, and the OD at 492 nm was determined using a microplate reader.

### Supplementary Information

**Additional file 1: Fig. S1.** ^1^H NMR spectrum of **1.** **Fig. S2.** ^13^C NMR spectrum of **1.** **Fig. S3.** HSQC spectrum of **1.** **Fig. S4.** HMBC spectrum of **1.** **Fig. S5.** ^1^H-^1^H COSY spectrum of **1.** **Fig. S6.** ROESY spectrum of **1.** **Fig. S7.** HRESIMS spectrum of **1.** **Fig. S8.** IR spectrum of **1.** **Fig. S9.** UV spectrum of **1.** **Fig. S10.** ^1^H NMR spectrum of **2.** **Fig. S11.** ^13^C NMR spectrum of **2.** **Fig. S12.** HSQC spectrum of **2.** **Fig. S13.** HMBC spectrum of **2.** **Fig. S14.** ^1^H-^1^H COSY spectrum of **2. Fig. S15.** ROESY spectrum of **2.** **Fig. S16.** HRESIMS spectrum of **2.** **Fig. S17.** IR spectrum of **2.** **Fig. S18.** UV spectrum of **2.** **Fig. S19.** ^1^H NMR spectrum of **3.**  **Fig. S20.** ^13^C NMR spectrum of **3.** **Fig. S21.** HSQC spectrum of **3.** **Fig. S22.** HMBC spectrum of **3.** **Fig. S23.** ^1^H-^1^H COSY spectrum of **3.** **Fig. S24.** ROESY spectrum of **3.** **Fig. S25.** HRESIMS spectrum of **3.** **Fig. S26.** IR spectrum of **3.** **Fig. S27.** UV spectrum of **3.** **Fig. S28.** ^1^H NMR spectrum of **4.** **Fig. S29.** ^13^C NMR spectrum of **4.** **Fig. S30.** HSQC spectrum of **4.** **Fig. S31** HMBC spectrum of **4.** **Fig. S32.** ^1^H-^1^H COSY spectrum of **4.**  **Fig. S33.** ROESY spectrum of **4.**  **Fig. S34.** HRESIMS spectrum of **4.** **Fig. S35.** IR spectrum of **4.** **Fig. S36.** UV spectrum of **4.**  **Fig. S37.** ^1^H NMR spectrum of **5.**  **Fig. S38.** ^13^C NMR spectrum of **5.** **Fig. S39.** HSQC spectrum of **5.**  **Fig. S40.** HMBC spectrum of **5.**  **Fig. S41.** ^1^H-^1^H COSY spectrum of **5.** **Fig. S42.** ROESY spectrum of **5.** **Fig. S43.** HRESIMS spectrum of **5.**  **Fig. S44.** IR spectrum of **5.**  **Fig. S45.** UV spectrum of **5.**  **Fig. S46.** ^1^H NMR spectrum of **6.**  **Fig. S47.** ^13^C NMR spectrum of **6.**  **Fig. S48.** HSQC spectrum of **6.** **Fig. S49.** HMBC spectrum of **6.** **Fig. S50.** ^1^H-^1^H COSY spectrum of **6.** **Fig. S51.** ROESY spectrum of **6.**  **Fig. S52.** HRESIMS spectrum of **6.** **Fig. S53.** IR spectrum of **6.** **Fig. S54.** UV spectrum of **6.** **Fig. S55.** ^1^H NMR spectrum of **7.** **Fig. S56.** ^13^C NMR spectrum of **7.** **Fig. S57.** ^1^H NMR spectrum of **8.**  **Fig. S58.** ^13^C NMR spectrum of **8.** **Fig. S59.** ^1^H NMR spectrum of **9.** **Fig. S60.** ^13^C NMR spectrum of **9.** **Fig. S61.** ^1^H NMR spectrum of **10.** **Fig. S62.** ^13^C NMR spectrum of **10.** **Fig. S63.** ^1^H NMR spectrum of **11.** **Fig. S64.** ^13^C NMR spectrum of **11.** **Fig. S65.** ^1^H NMR spectrum of **12.** **Fig. S66.** ^13^C NMR spectrum of **12.** **Fig. S67.** ^1^H NMR spectrum of **13.** **Fig. S68.** ^13^C NMR spectrum of **13.** **Fig. S69.** ^1^H NMR spectrum of **14.** **Fig. S70.** ^13^C NMR spectrum of **14.** **Fig. S71.** ^1^H NMR spectrum of **15.** **Fig. S72.** ^13^C NMR spectrum of **15.** **Fig. S73.** ^1^H NMR spectrum of **16.** **Fig. S74.** ^13^C NMR spectrum of **16.** **Fig. S75.** ^1^H NMR spectrum of **17.** **Fig. S76** ^13^C NMR spectrum of **17.** **Fig. S77**. Correlations between calculated and experimental ^13^C NMR chemical shifts of **1A** and **1B.** **Fig. S78**. DP4 + analysis results of **1.** **Table S1.** Energy analysis for conformers of **1Aa** ~ **1Ae** at mpw1pw91/6–31 + g(d,p) level in the gas phase. **Table S2**. Cartesian coordinates for the low-energy optimized conformers of **1A** at M062X/def2svp level. **Fig. S79.** Correlations between calculated and experimental ^13^C NMR chemical shifts of **2A** and **2B.** **Fig. S80**. DP4 + analysis results of **2.** **Table S3.** Energy analysis for conformers of **2Aa** ~ **2Ae** at mpw1pw91/6–31 + g(d,p) level in the gas phase. **Table S4**. Cartesian coordinates for the low-energy optimized conformers of **2A** at M062X/def2svp level. **Fig. S81.** Correlations between calculated and experimental.^13^C NMR chemical shifts of **4A** and **4B.** **Fig. S82.** DP4 + analysis results of **4**  **Table S5.** Energy analysis for conformers of **4Aa** ~ **4Ae** at mpw1pw91/6–31 + g(d,p) level in the gas phase **Table S6.** Cartesian coordinates for the low-energy optimized conformers of **4A** at M062X/def2svp level. **Table S7.** Inhibitory effect of compounds on four strains of bacteria. **Table S8.** Inhibitory effect of compounds on five strains of Cytotoxicity.

## Data Availability

All data generated and analyzed during this study are included in this published article and its Additional file.
