# Supplementary material for: The antifungal properties of terpenoids from the endophytic fungus Bipolaris eleusines
Source: Nat Prod Bioprospect. 2023 Oct 23;13(1):43. doi: 10.1007/s13659-023-00407-x (PMC10593648; doi:10.1007/s13659-023-00407-x)
Supplement: Supplementary file 1 — Additional file 1: Fig. S1. 1H NMR spectrum of 1. Fig. S2. 13C NMR spectrum of 1. Fig. S3. HSQC spectrum of 1. Fig. S4. HMBC spectrum of 1. Fig. S5. 1H-1H COSY spectrum of 1. Fig. S6. ROESY spectrum of 1. Fig. S7. HRESIMS spectrum of 1. Fig. S8. IR spectrum of 1. Fig. S9. UV spectrum of 1. Fig. S10. 1H NMR spectrum of 2. Fig. S11. 13C NMR spectrum of 2. Fig. S12. HSQC spectrum of 2. Fig. S13. HMBC spectrum of 2. Fig. S14. 1H-1H COSY spectrum of 2. Fig. S15. ROESY spectrum of 2. Fig. S16. HRESIMS spectrum of 2. Fig. S17. IR spectrum of 2. Fig. S18. UV spectrum of 2. Fig. S19. 1H NMR spectrum of 3. Fig. S20. 13C NMR spectrum of 3. Fig. S21. HSQC spectrum of 3. Fig. S22. HMBC spectrum of 3. Fig. S23. 1H-1H COSY spectrum of 3. Fig. S24. ROESY spectrum of 3. Fig. S25. HRESIMS spectrum of 3. Fig. S26. IR spectrum of 3. Fig. S27. UV spectrum of 3. Fig. S28. 1H NMR spectrum of 4. Fig. S29. 13C NMR spectrum of 4. Fig. S30. HSQC spectrum of 4. Fig. S31 HMBC spectrum of 4. Fig. S32. 1H-1H COSY spectrum of 4. Fig. S33. ROESY spectrum of 4. Fig. S34. HRESIMS spectrum of 4. Fig. S35. IR spectrum of 4. Fig. S36. UV spectrum of 4. Fig. S37. 1H NMR spectrum of 5. Fig. S38. 13C NMR spectrum of 5. Fig. S39. HSQC spectrum of 5. Fig. S40. HMBC spectrum of 5. Fig. S41. 1H-1H COSY spectrum of 5. Fig. S42. ROESY spectrum of 5. Fig. S43. HRESIMS spectrum of 5. Fig. S44. IR spectrum of 5. Fig. S45. UV spectrum of 5. Fig. S46. 1H NMR spectrum of 6. Fig. S47. 13C NMR spectrum of 6. Fig. S48. HSQC spectrum of 6. Fig. S49. HMBC spectrum of 6. Fig. S50. 1H-1H COSY spectrum of 6. Fig. S51. ROESY spectrum of 6. Fig. S52. HRESIMS spectrum of 6. Fig. S53. IR spectrum of 6. Fig. S54. UV spectrum of 6. Fig. S55. 1H NMR spectrum of 7. Fig. S56. 13C NMR spectrum of 7. Fig. S57. 1H NMR spectrum of 8. Fig. S58. 13C NMR spectrum of 8. Fig. S59. 1H NMR spectrum of 9. Fig. S60. 13C NMR spectrum of 9. Fig. S61. 1H NMR spectrum of 10. Fig. S62. 13C NMR spectrum of 10. Fig. S63. 1H NMR spectrum of 11. Fig. S64. [file 13659_2023_407_MOESM1_ESM.docx]

Supplementary Material

The antifungal properties of terpenoids from the potato endophytic fungus *Bipolaris eleusines*

Yin-Zhong Fan, Chun Tian, Shun-Yao Tong, Qing Liu, Fan Xu, Bao-Bao Shi^⁎^, Hong-Lian Ai^⁎^, and Ji-Kai Liu^⁎^

*School of Pharmaceutical Sciences, South-Central University for Nationalities, Wuhan 430074, People’s Republic of China*

**Corresponding authors.
E-mail addresses: shibb0505@163.com (B.-B. Shi.);aihonglian@mail.scuec.edu.cn (H.-L. Ai); liujikai@mail.scuec.edu.cn (J.-K. Liu).*

Table of Contents

[**Spectroscopic data** 7](#_Toc138233033)

[**Fig. S1** ^1^H NMR spectrum of **1.** 7](#_Toc138233034)

[**Fig. S2** ^13^C NMR spectrum of **1.** 8](#_Toc138233035)

[**Fig. S3** HSQC spectrum of **1.** 9](#_Toc138233036)

[**Fig. S4** HMBC spectrum of **1.** 10](#_Toc138233037)

[**Fig. S5** ^1^H-^1^H COSY spectrum of **1.** 11](#_Toc138233038)

[**Fig. S6** ROESY spectrum of **1.** 12](#_Toc138233039)

[**Fig. S7** HRESIMS spectrum of **1.** 13](#_Toc138233040)

[**Fig. S8** IR spectrum of **1.** 14](#_Toc138233041)

[**Fig. S9** UV spectrum of **1.** 15](#_Toc138233042)

[**Fig. S10** ^1^H NMR spectrum of **2.** 16](#_Toc138233043)

[**Fig. S11** ^13^C NMR spectrum of **2.** 17](#_Toc138233044)

[**Fig. S12** HSQC spectrum of **2.** 18](#_Toc138233045)

[**Fig. S13** HMBC spectrum of **2.** 19](#_Toc138233046)

[**Fig. S14** ^1^H-^1^H COSY spectrum of **2.** 20](#_Toc138233047)

[**Fig. S15** ROESY spectrum of **2.** 21](#_Toc138233048)

[**Fig. S16** HRESIMS spectrum of **2.** 22](#_Toc138233049)

[**Fig. S17** IR spectrum of **2.** 23](#_Toc138233050)

[**Fig. S18** UV spectrum of **2.** 24](#_Toc138233051)

[**Fig. S19** ^1^H NMR spectrum of **3.** 25](#_Toc138233052)

[**Fig. S20** ^13^C NMR spectrum of **3.** 26](#_Toc138233053)

[**Fig. S21** HSQC spectrum of **3.** 27](#_Toc138233054)

[**Fig. S22** HMBC spectrum of **3.** 28](#_Toc138233055)

[**Fig. S23** ^1^H-^1^H COSY spectrum of **3.** 29](#_Toc138233056)

[**Fig. S24** ROESY spectrum of **3.** 30](#_Toc138233057)

[**Fig. S25** HRESIMS spectrum of **3.** 31](#_Toc138233058)

[**Fig. S26** IR spectrum of **3.** 32](#_Toc138233059)

[**Fig. S27** UV spectrum of **3.** 33](#_Toc138233060)

[**Fig. S28** ^1^H NMR spectrum of **4.** 34](#_Toc138233061)

[**Fig. S29** ^13^C NMR spectrum of **4.** 35](#_Toc138233062)

[**Fig. S30** HSQC spectrum of **4.** 36](#_Toc138233063)

[**Fig. S31** HMBC spectrum of **4.** 37](#_Toc138233064)

[**Fig. S32** ^1^H-^1^H COSY spectrum of **4.** 38](#_Toc138233065)

[**Fig. S33** ROESY spectrum of **4.** 39](#_Toc138233066)

[**Fig. S34** HRESIMS spectrum of **4.** 40](#_Toc138233067)

[**Fig. S35** IR spectrum of **4.** 41](#_Toc138233068)

[**Fig. S36** UV spectrum of **4.** 42](#_Toc138233069)

[**Fig. S37** ^1^H NMR spectrum of **5.** 43](#_Toc138233070)

[**Fig. S38** ^13^C NMR spectrum of **5.** 44](#_Toc138233071)

[**Fig. S39** HSQC spectrum of **5.** 45](#_Toc138233072)

[**Fig. S40** HMBC spectrum of **5.** 46](#_Toc138233073)

[**Fig. S41** ^1^H-^1^H COSY spectrum of **5.** 47](#_Toc138233074)

[**Fig. S42** ROESY spectrum of **5.** 48](#_Toc138233075)

[**Fig. S43** HRESIMS spectrum of **5.** 49](#_Toc138233076)

[**Fig. S44** IR spectrum of **5.** 50](#_Toc138233077)

[**Fig. S45** UV spectrum of **5.** 51](#_Toc138233078)

[**Fig. S46** ^1^H NMR spectrum of **6.** 52](#_Toc138233079)

[**Fig. S47** ^13^C NMR spectrum of **6.** 53](#_Toc138233080)

[**Fig. S48** HSQC spectrum of **6.** 54](#_Toc138233081)

[**Fig. S49** HMBC spectrum of **6.** 55](#_Toc138233082)

[**Fig. S50** ^1^H-^1^H COSY spectrum of **6.** 56](#_Toc138233083)

[**Fig. S51** ROESY spectrum of **6.** 57](#_Toc138233084)

[**Fig. S52** HRESIMS spectrum of **6.** 58](#_Toc138233085)

[**Fig. S53** IR spectrum of **6.** 59](#_Toc138233086)

[**Fig. S54** UV spectrum of **6.** 60](#_Toc138233087)

[**Fig. S55** ^1^H NMR spectrum of **7.** 61](#_Toc138233088)

[**Fig. S56** ^13^C NMR spectrum of **7.** 62](#_Toc138233089)

[**Fig. S57** ^1^H NMR spectrum of **8.** 63](#_Toc138233090)

[**Fig. S58** ^13^C NMR spectrum of **8.** 64](#_Toc138233091)

[**Fig. S59** ^1^H NMR spectrum of **9.** 66](#_Toc138233092)

[**Fig. S60** ^13^C NMR spectrum of **9.** 67](#_Toc138233093)

[**Fig. S61** ^1^H NMR spectrum of **10.** 68](#_Toc138233094)

[**Fig. S62** ^13^C NMR spectrum of **10.** 69](#_Toc138233095)

[**Fig. S63** ^1^H NMR spectrum of **11.** 70](#_Toc138233096)

[**Fig. S64** ^13^C NMR spectrum of **11.** 71](#_Toc138233097)

[**Fig. S65** ^1^H NMR spectrum of **12.** 72](#_Toc138233098)

[**Fig. S66** ^13^C NMR spectrum of **12.** 73](#_Toc138233099)

[**Fig. S67** ^1^H NMR spectrum of **13.** 74](#_Toc138233100)

[**Fig. S68** ^13^C NMR spectrum of **13.** 75](#_Toc138233101)

[**Fig. S69** ^1^H NMR spectrum of **14.** 76](#_Toc138233102)

[**Fig. S70** ^13^C NMR spectrum of **14.** 77](#_Toc138233103)

[**Fig. S71** ^1^H NMR spectrum of **15.** 78](#_Toc138233104)

[**Fig. S72** ^13^C NMR spectrum of **15.** 79](#_Toc138233105)

[**Fig. S73** ^1^H NMR spectrum of **16.** 80](#_Toc138233106)

[**Fig. S74** ^13^C NMR spectrum of **16.** 81](#_Toc138233107)

[**Fig. S75** ^1^H NMR spectrum of **17.** 82](#_Toc138233108)

[**Fig. S76** ^13^C NMR spectrum of **17.** 83](#_Toc138233109)

[**Quantum chemical calculation** 84](#_Toc138233110)

[**Fig. S77**. Correlations between calculated and experimental ^13^C NMR chemical shifts of **1A** and **1B.** 84](#_Toc138233111)

[**Fig. S78**. DP4+ analysis results of **1.** 85](#_Toc138233112)

[**Table S1.** Energy analysis for conformers of **1Aa**~**1Ae** at mpw1pw91/6-31+g(d,p) level in the gas phase. 86](#_Toc138233113)

[**Table S2**. Cartesian coordinates for the low-energy optimized conformers of **1A** at M062X/def2svp level. 87](#_Toc138233114)

[**Fig. S79.** Correlations between calculated and experimental ^13^C NMR chemical shifts of **2A** and **2B.** 92](#_Toc138233115)

[**Fig. S80**. DP4+ analysis results of **2.** 93](#_Toc138233116)

[**Table S3.** Energy analysis for conformers of **2Aa**~**2Ae** at mpw1pw91/6-31+g(d,p) level in the gas phase. 94](#_Toc138233117)

[**Table S4**. Cartesian coordinates for the low-energy optimized conformers of **2A** at M062X/def2svp level. 95](#_Toc138233118)

[**Fig. S81.** Correlations between calculated and experimental ^13^C NMR chemical shifts of **4A** and **4B** 100](#_Toc138233119)

[**Fig. S82.** DP4+ analysis results of **4** 101](#_Toc138233120)

[**Table S5.** Energy analysis for conformers of **4Aa**~**4Ae** at mpw1pw91/6-31+g(d,p) level in the gas phase 102](#_Toc138233121)

[**Table S6.** Cartesian coordinates for the low-energy optimized conformers of **4A** at M062X/def2svp level. 103](#_Toc138233122)

[**Table S7** Inhibitory effect of compounds on four strains of bacteria. 108](#_Toc138233123)

[**Table S8** Inhibitory effect of compounds on five strains of Cytotoxicity. 108](#_Toc138233124)

Spectroscopic data
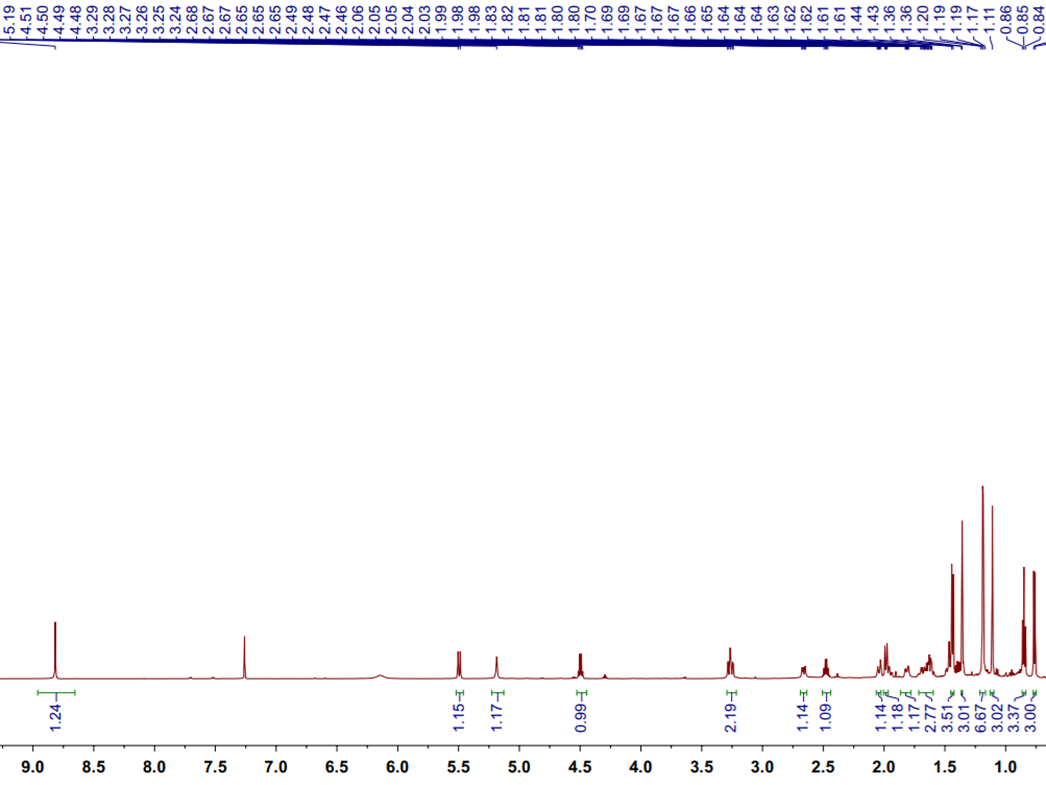


**Fig. S1** ^1^H NMR spectrum of **1.**


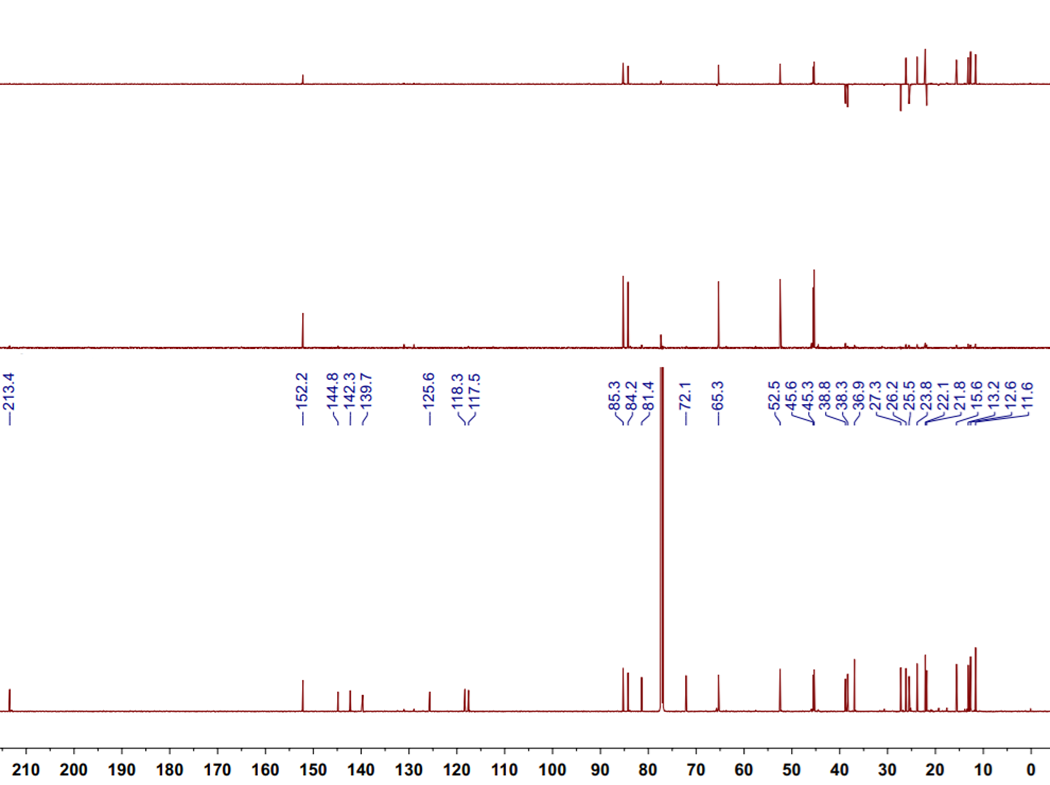


**Fig. S2** ^13^C NMR spectrum of **1.**


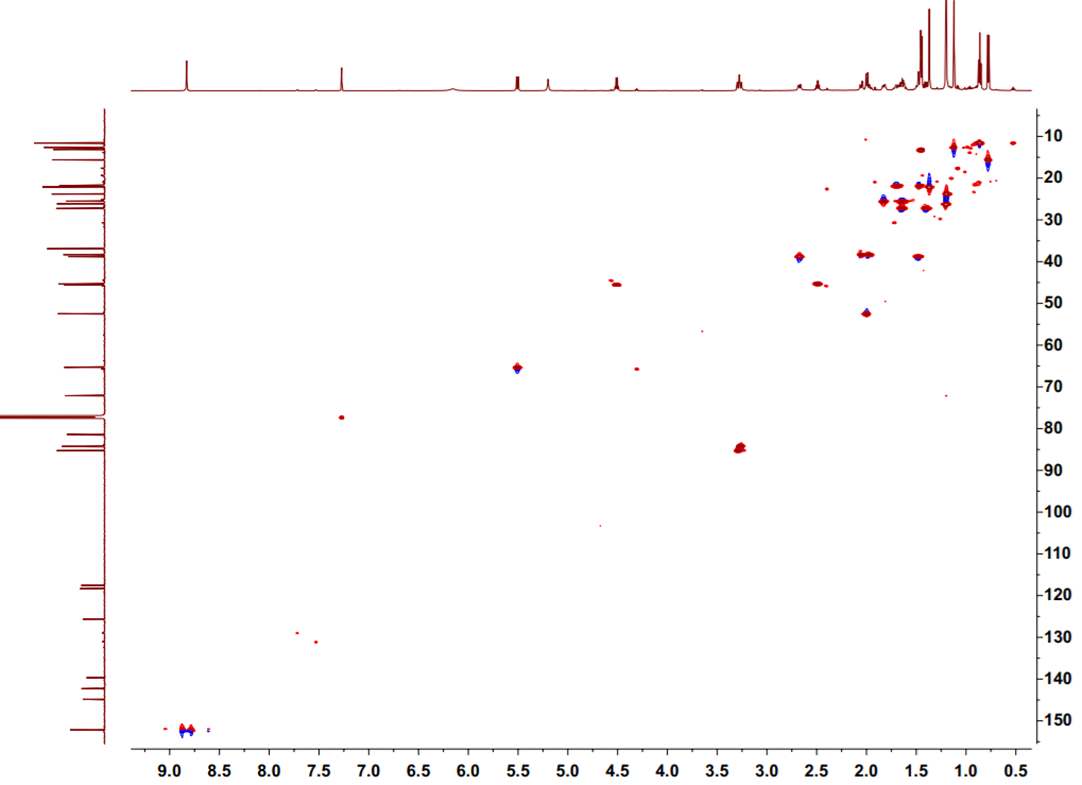


**Fig. S3** HSQC spectrum of **1.**

**
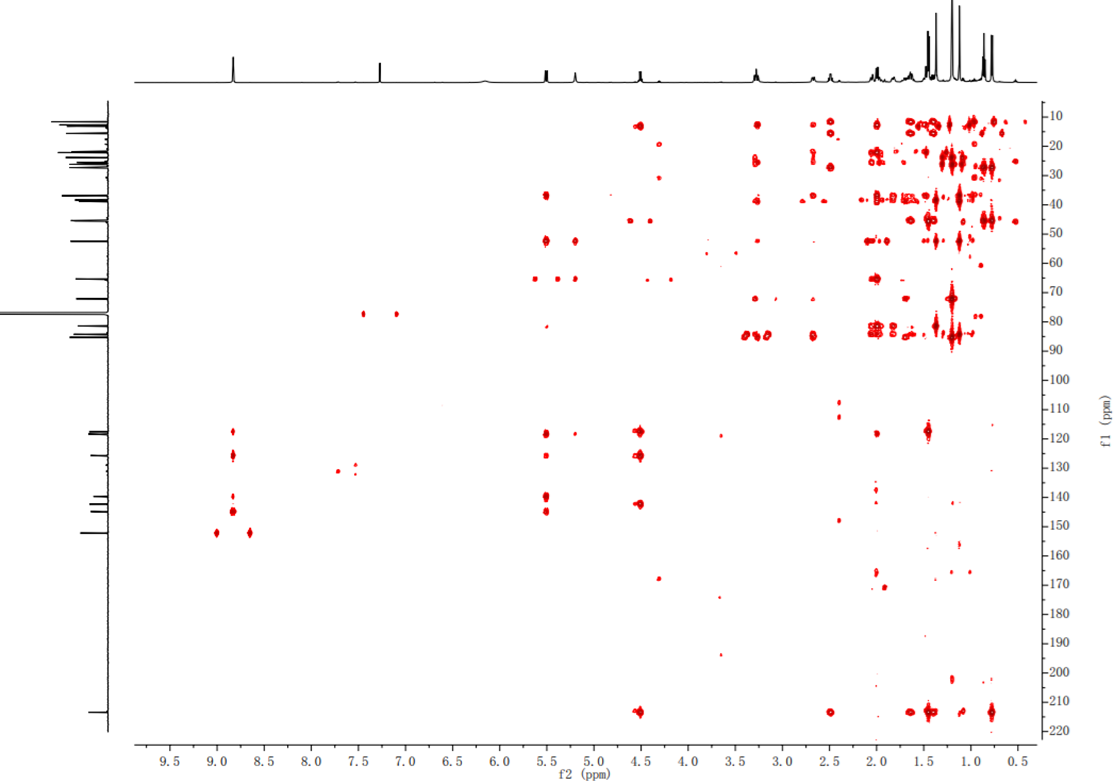
**

**Fig. S4** HMBC spectrum of **1.**

**
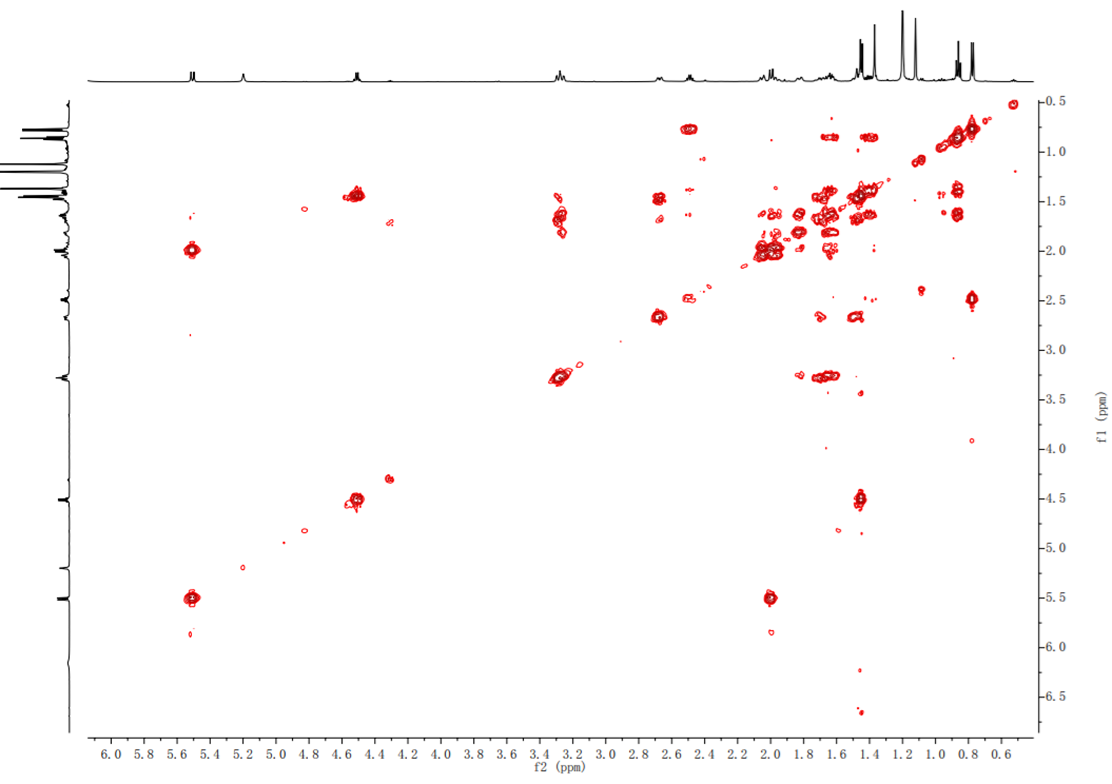
**

**Fig. S5** ^1^H-^1^H COSY spectrum of **1.**

**
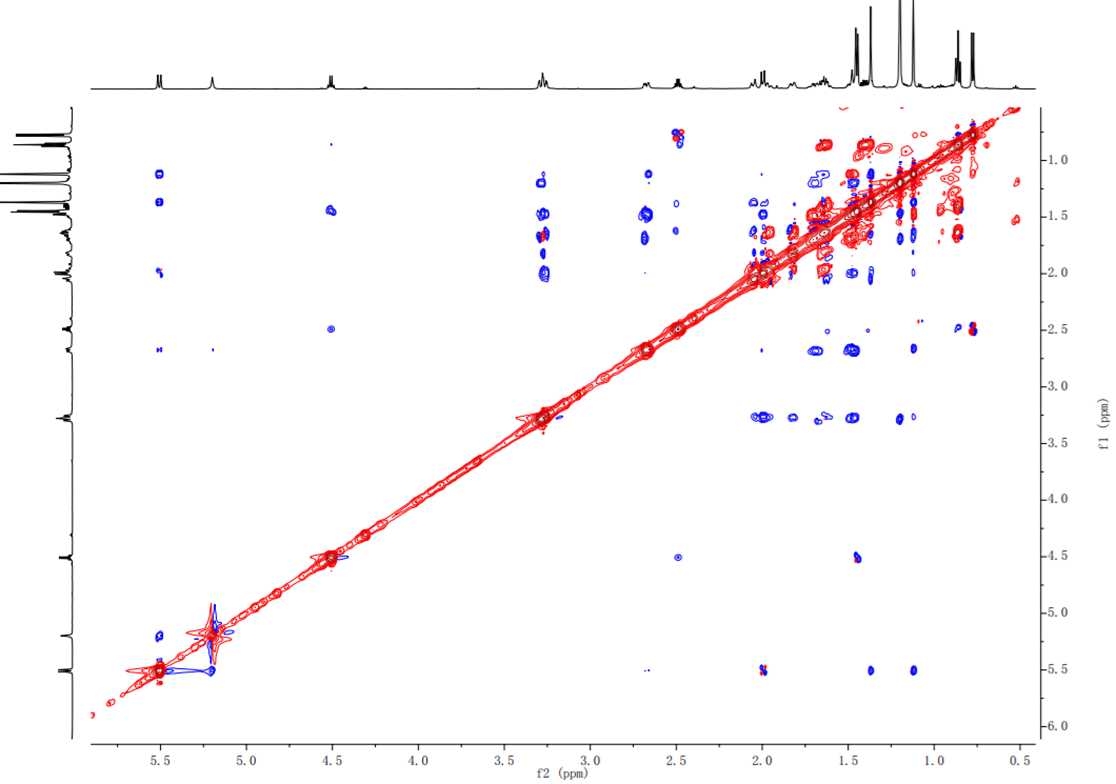
**

**Fig. S6** ROESY spectrum of **1.**


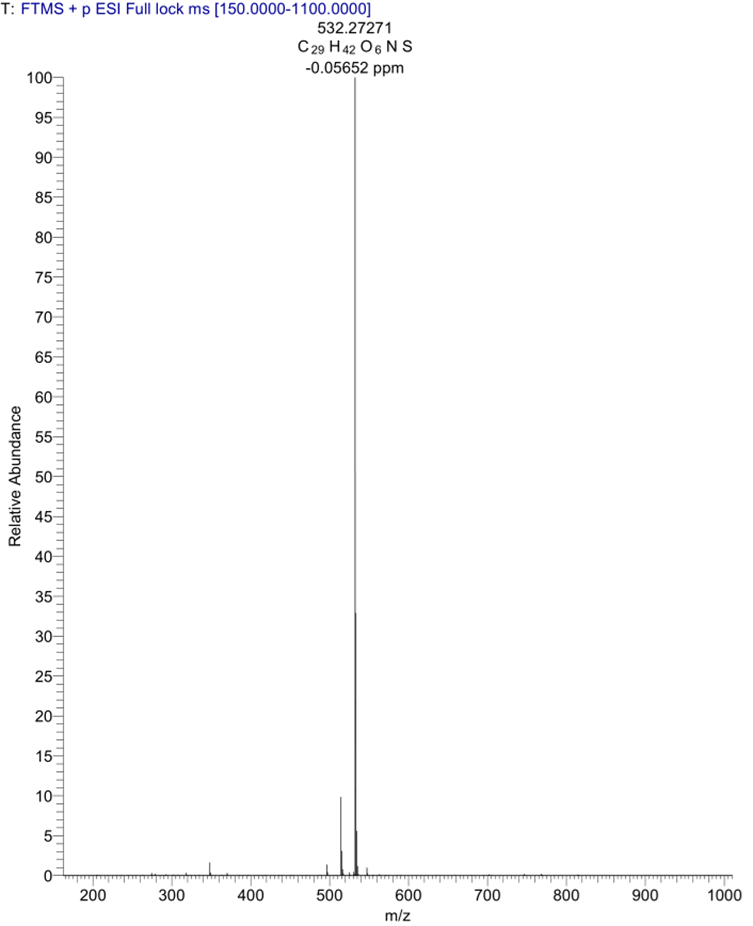


**Fig. S7** HRESIMS spectrum of **1.**


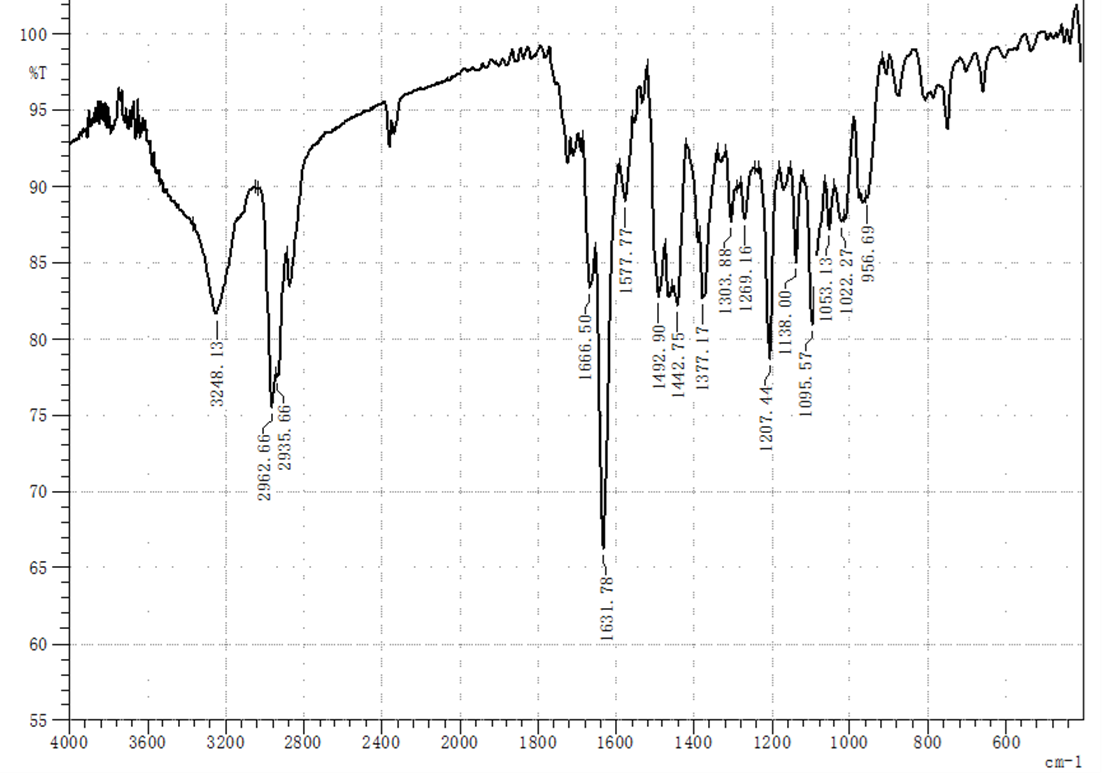


**Fig. S8** IR spectrum of **1.**


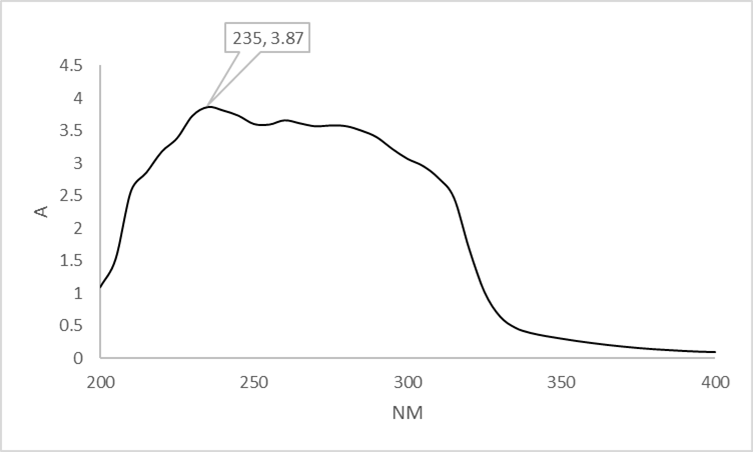


**Fig. S9** UV spectrum of **1.**


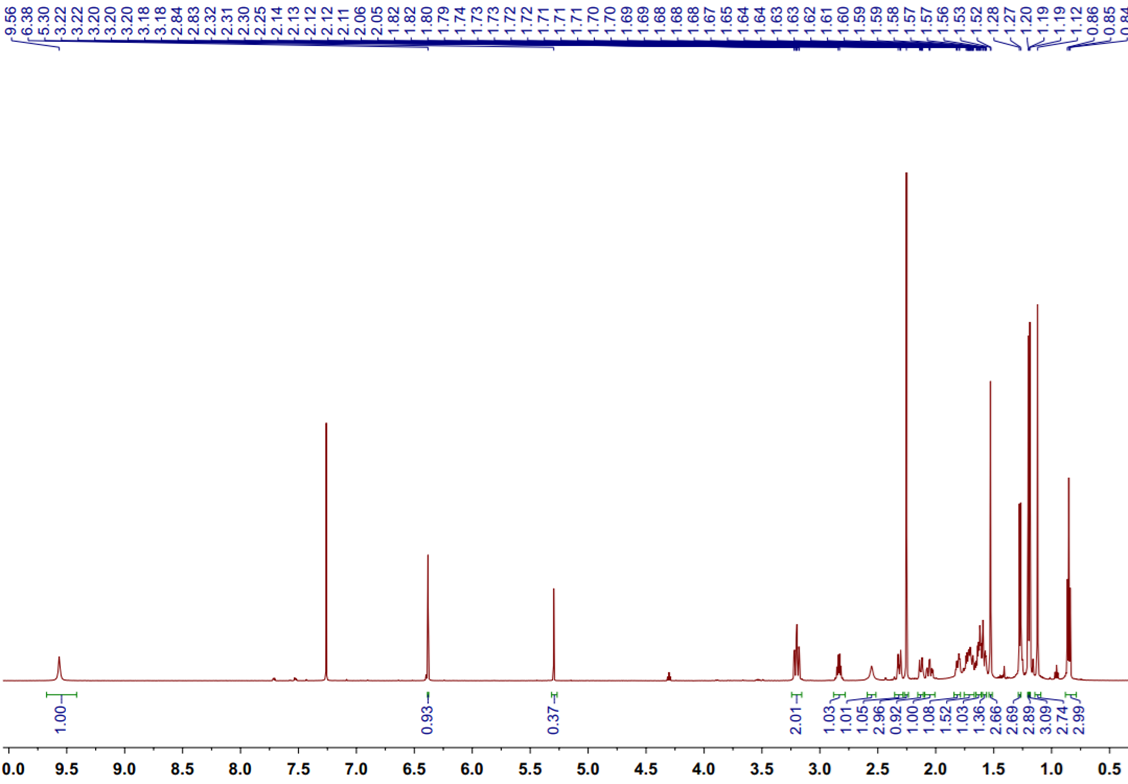


**Fig. S10** ^1^H NMR spectrum of **2.**


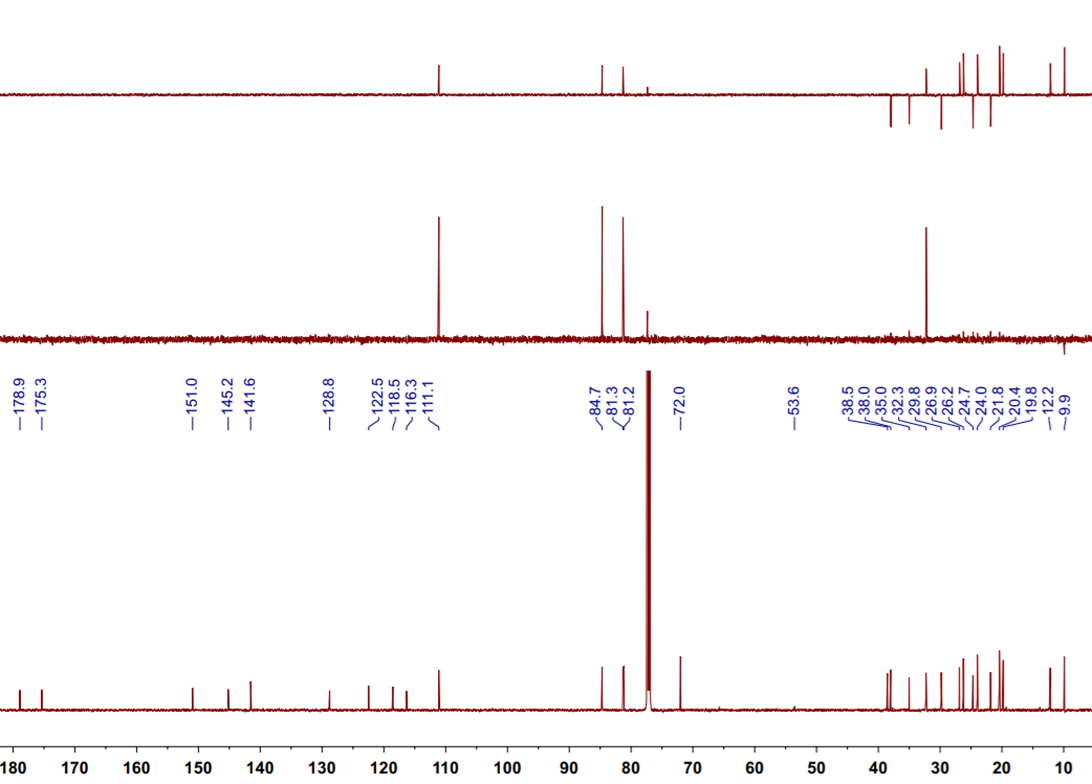


**Fig. S11** ^13^C NMR spectrum of **2.**


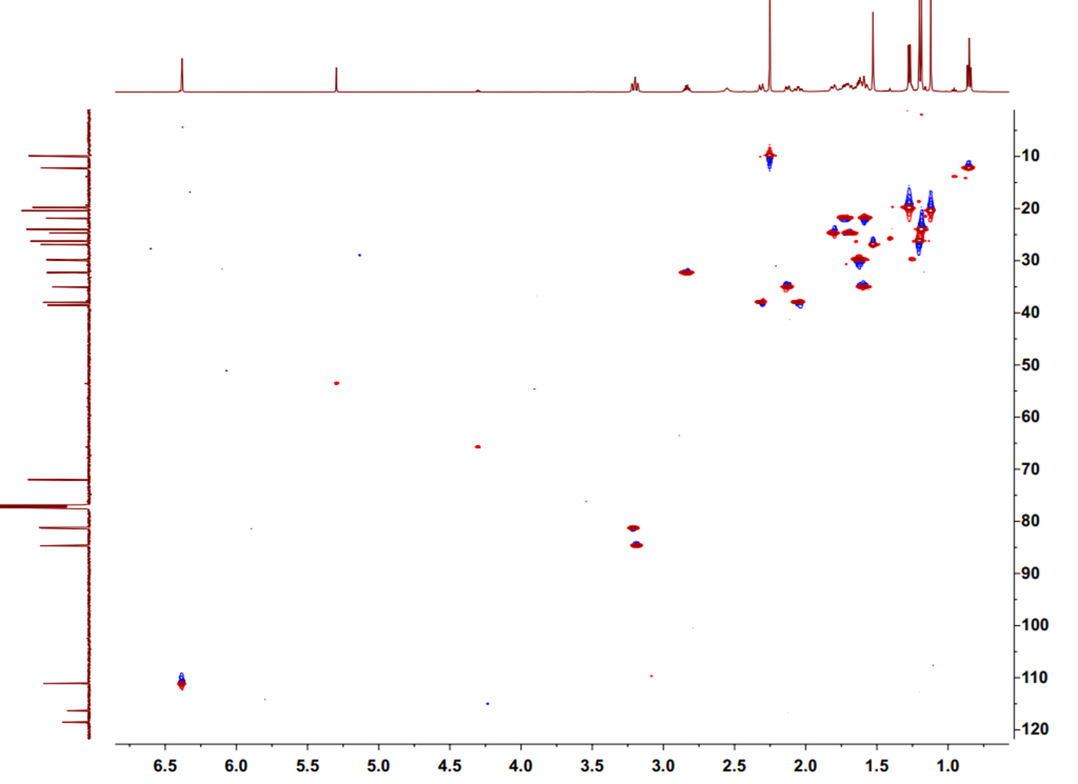


**Fig. S12** HSQC spectrum of **2.**


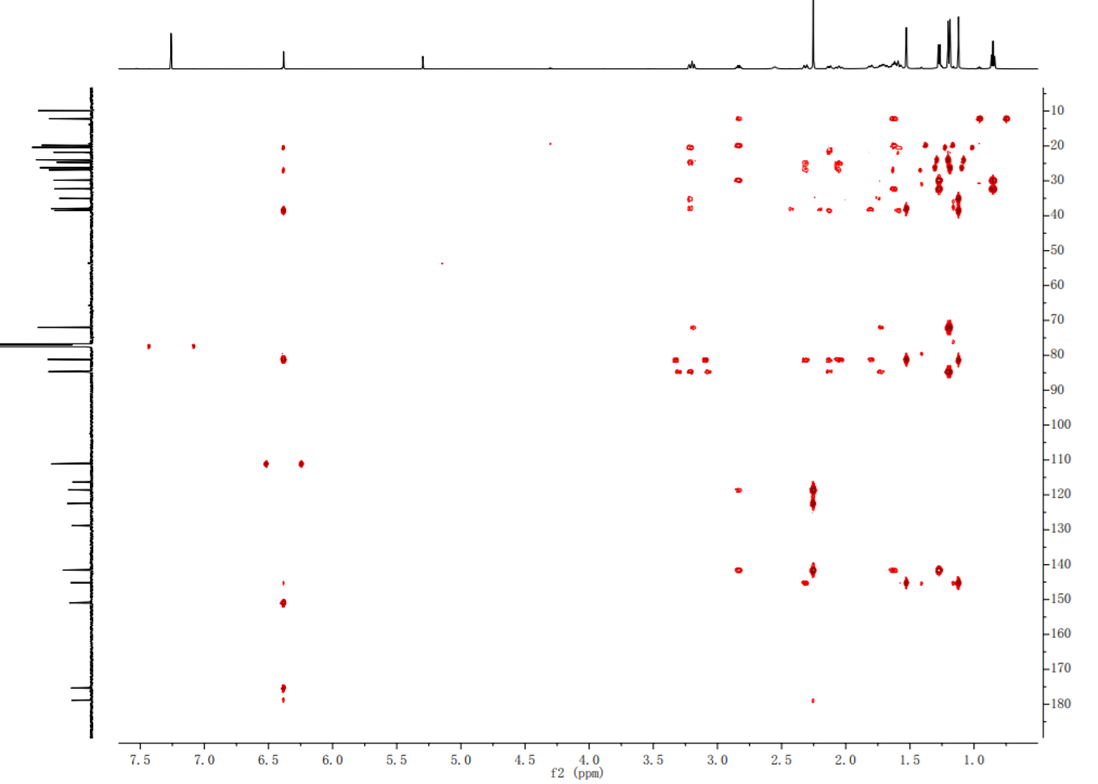


**Fig. S13** HMBC spectrum of **2.**


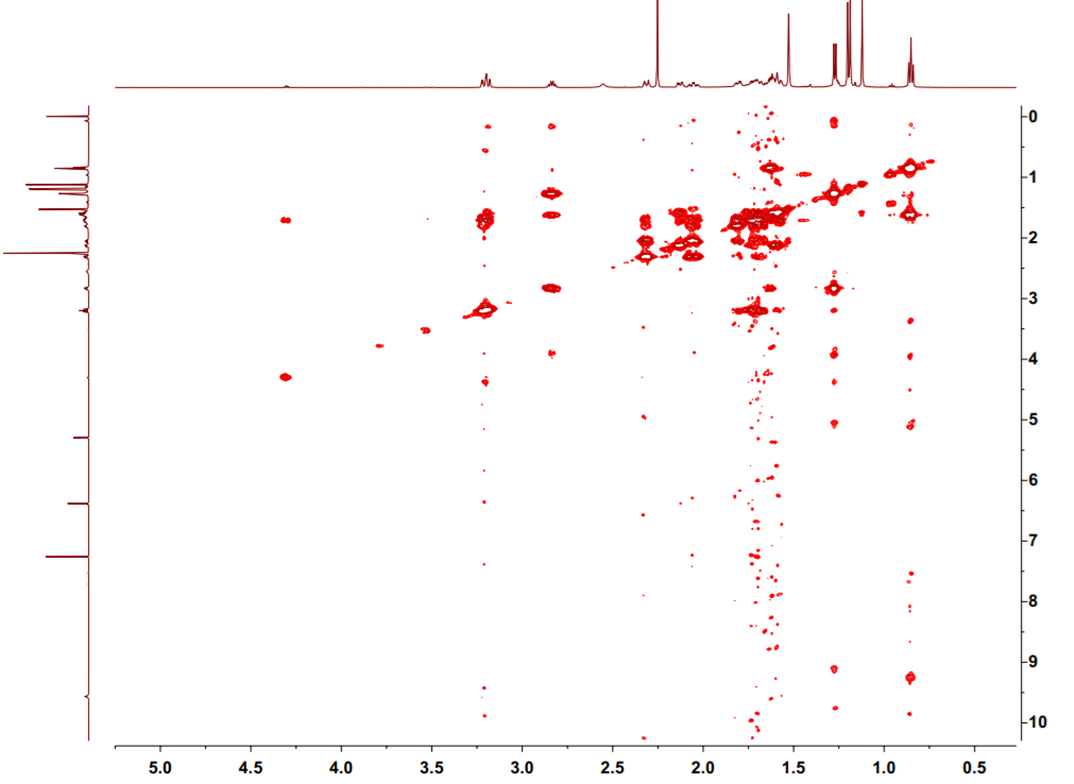


**Fig. S14** ^1^H-^1^H COSY spectrum of **2.**


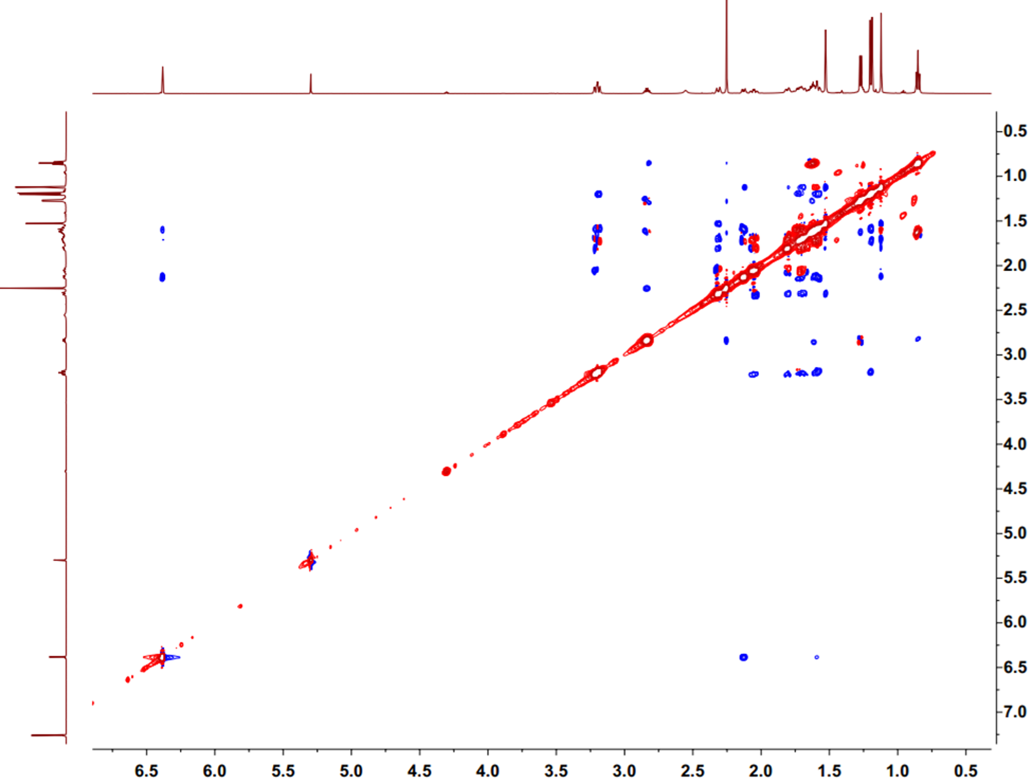


**Fig. S15** ROESY spectrum of **2.**

**
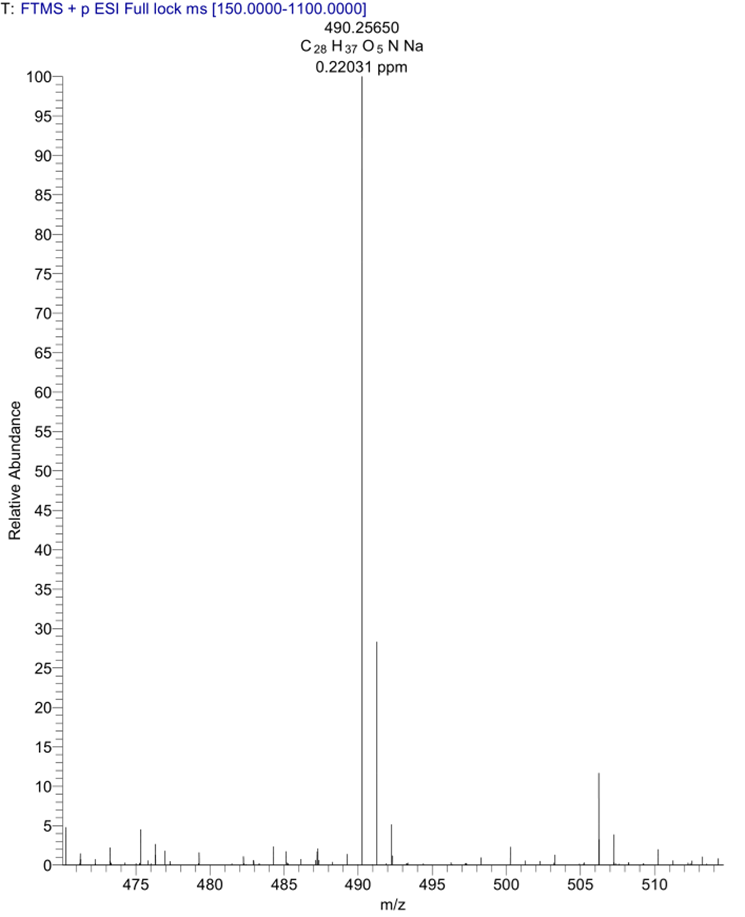
**

**Fig. S16** HRESIMS spectrum of **2.**

**
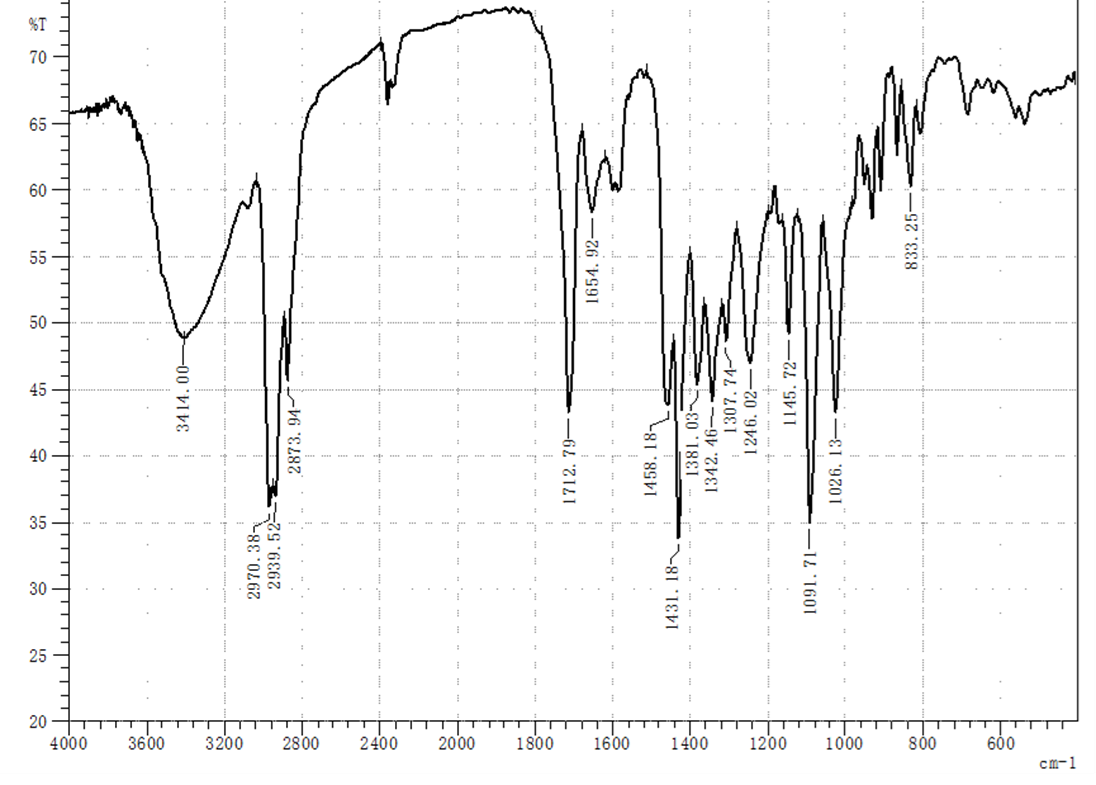
**

**Fig. S17** IR spectrum of **2.**


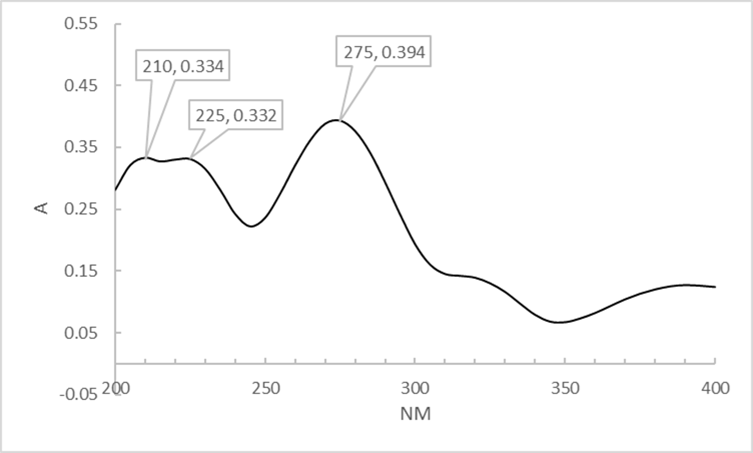


**Fig. S18** UV spectrum of **2.**


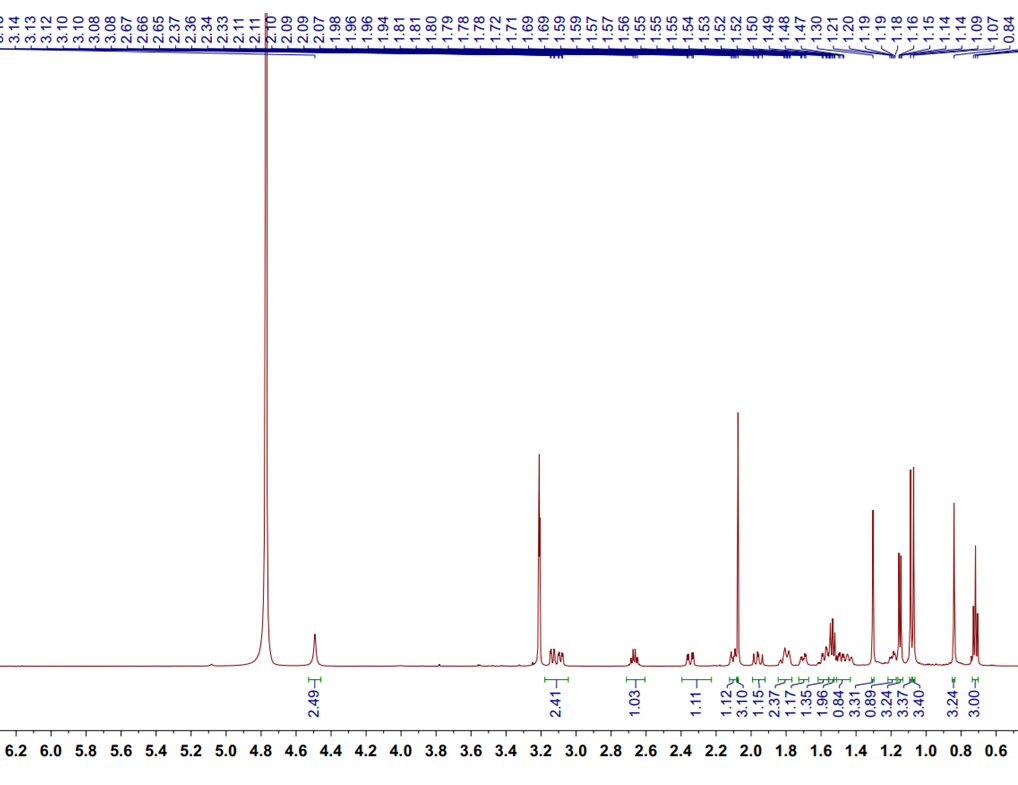


**Fig. S19** ^1^H NMR spectrum of **3.**


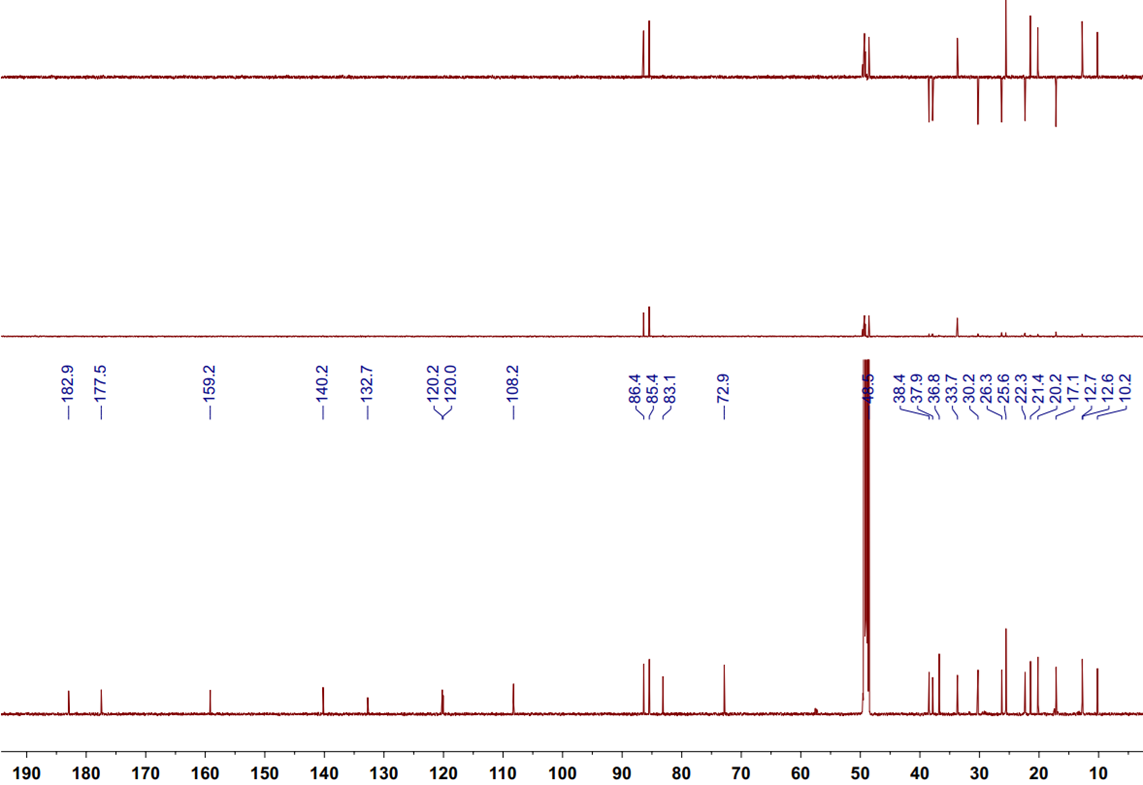


**Fig. S20** ^13^C NMR spectrum of **3.**


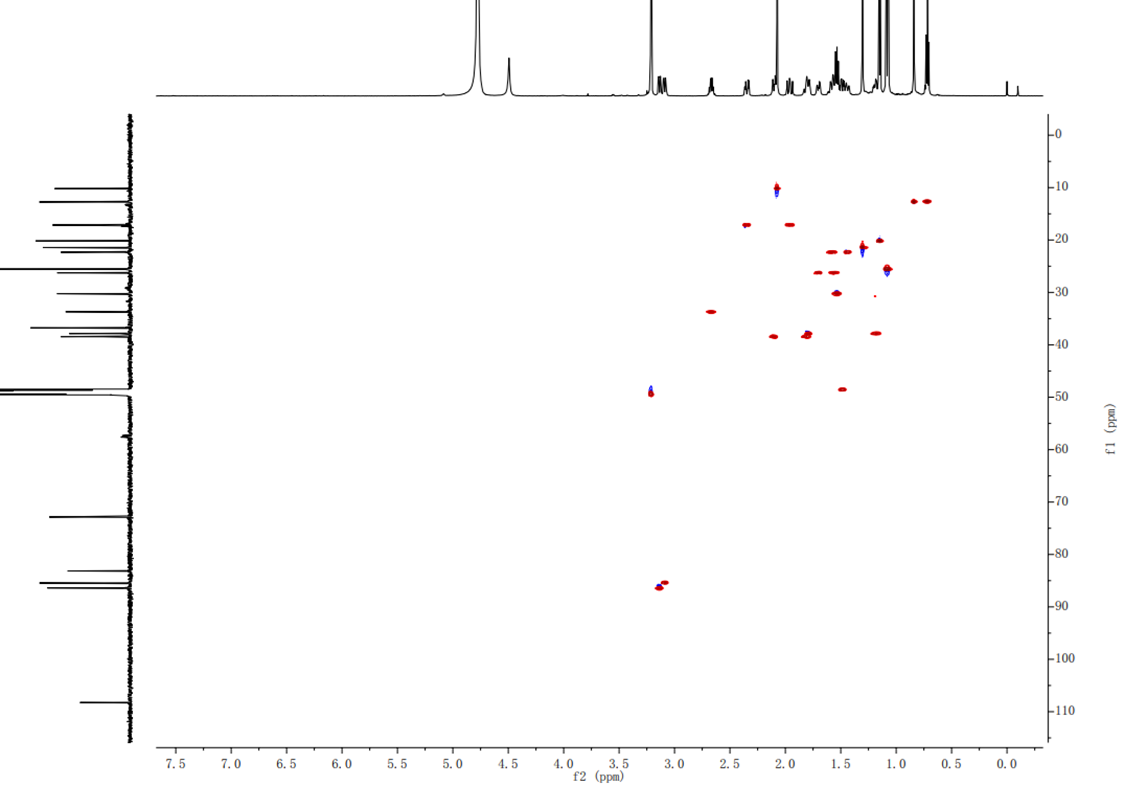


**Fig. S21** HSQC spectrum of **3.**


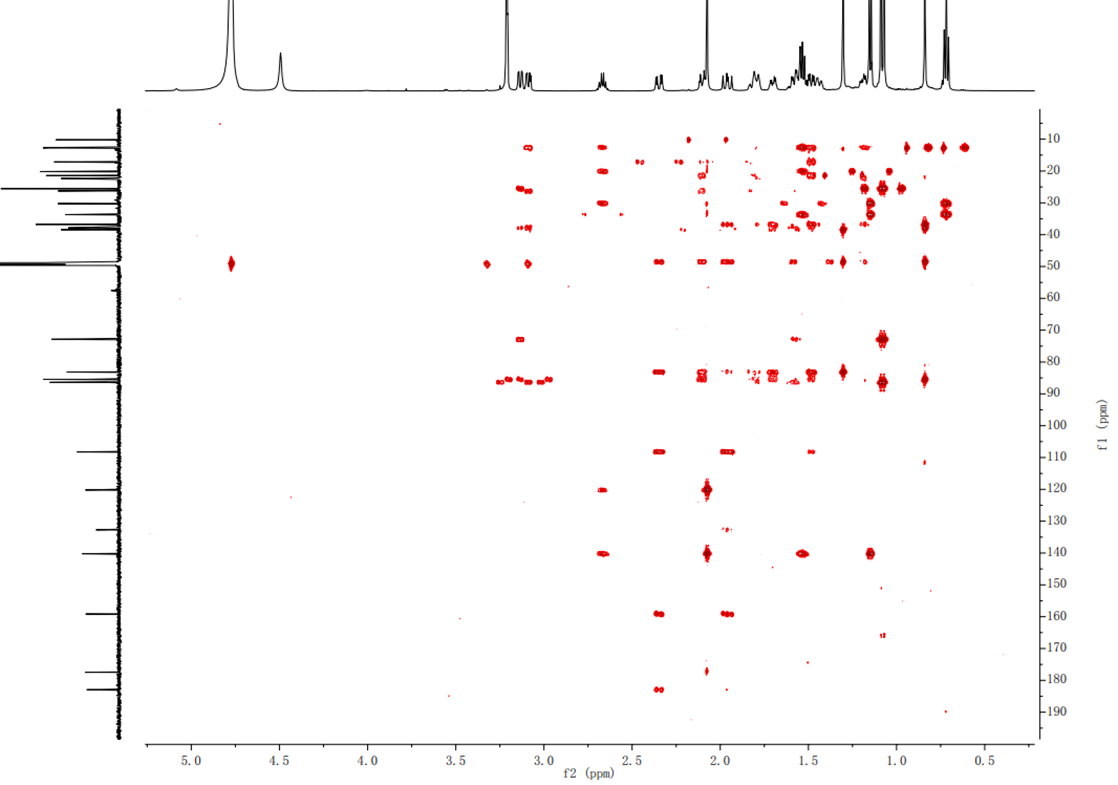


**Fig. S22** HMBC spectrum of **3.**


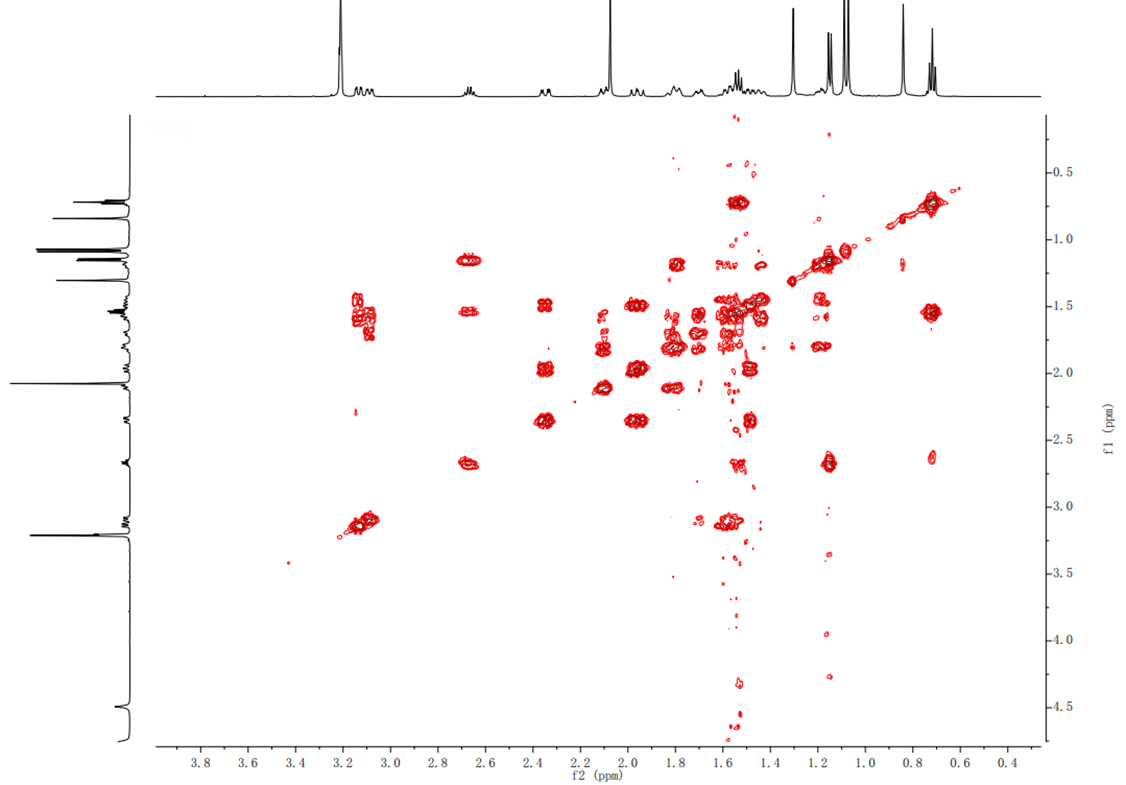


**Fig. S23** ^1^H-^1^H COSY spectrum of **3.**


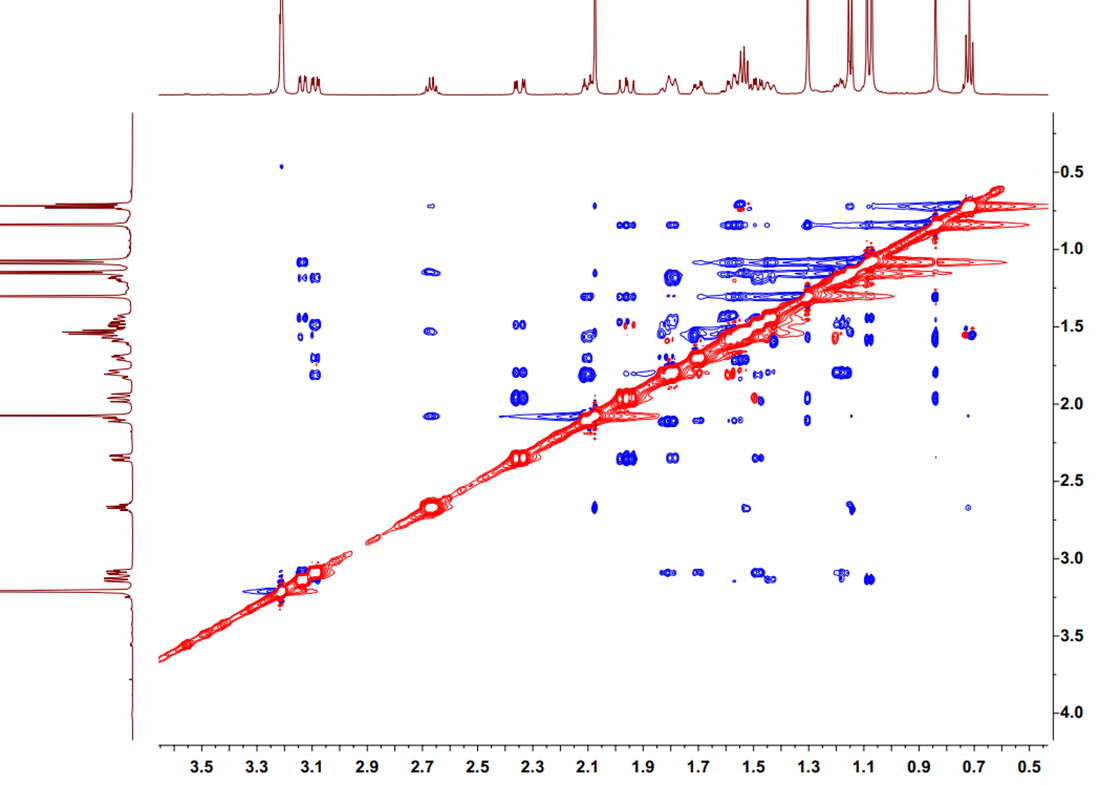


**Fig. S24** ROESY spectrum of **3.**


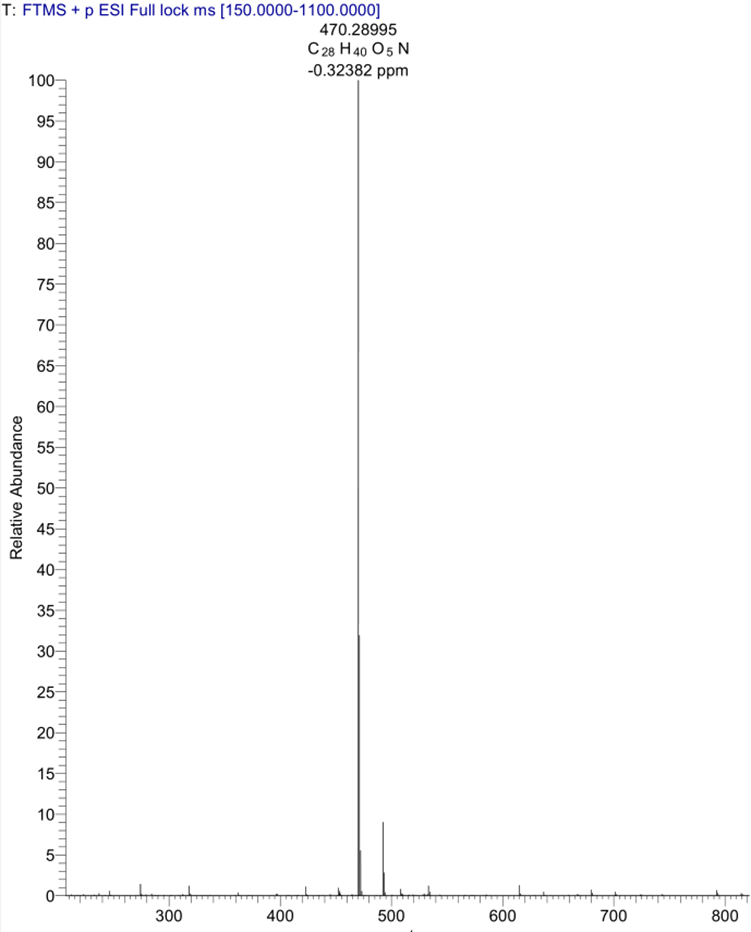


**Fig. S25** HRESIMS spectrum of **3.**


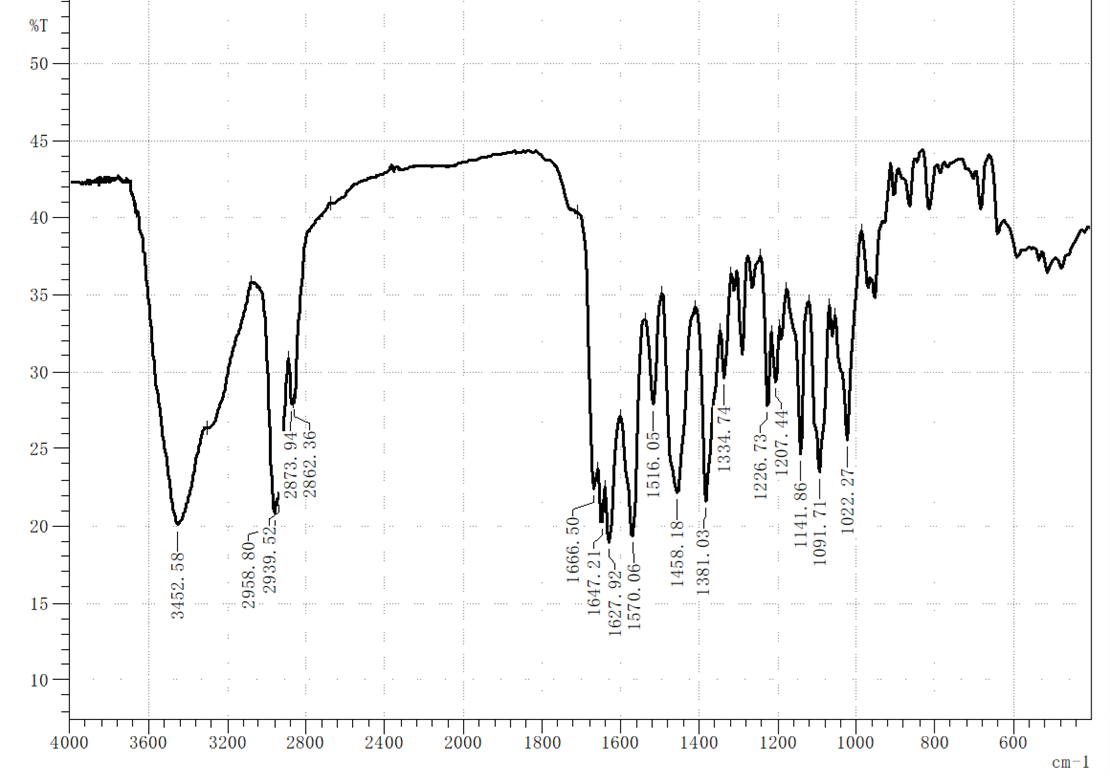


**Fig. S26** IR spectrum of **3.**


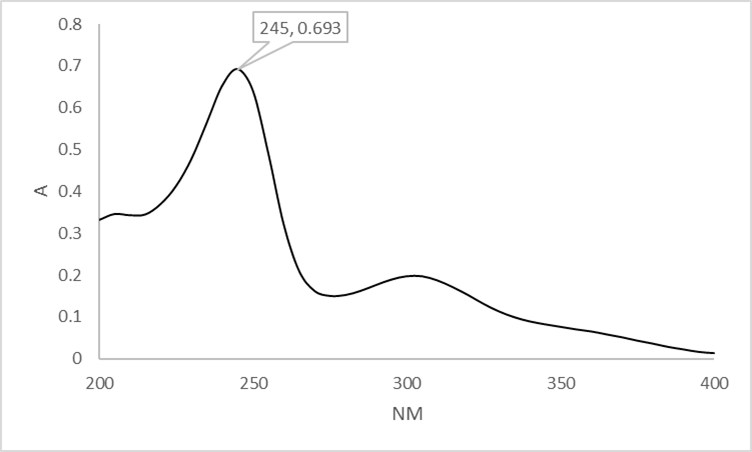


**Fig. S27** UV spectrum of **3.**


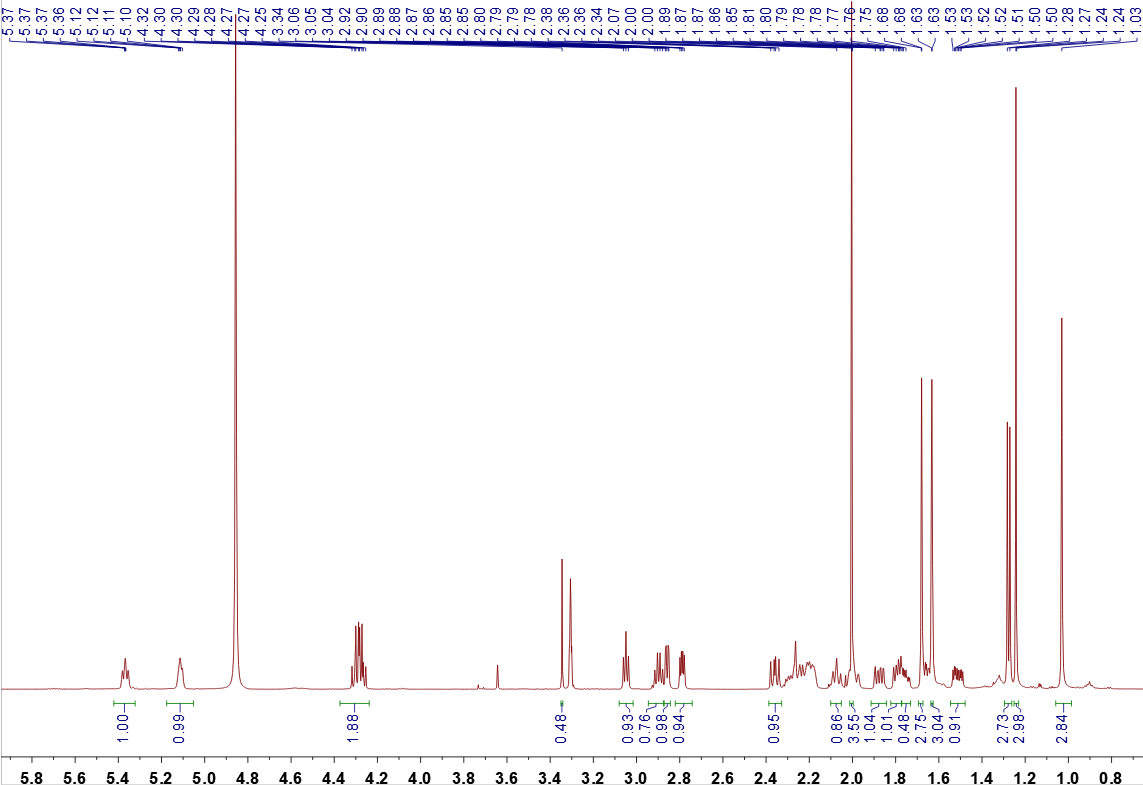


**Fig. S28** ^1^H NMR spectrum of **4.**


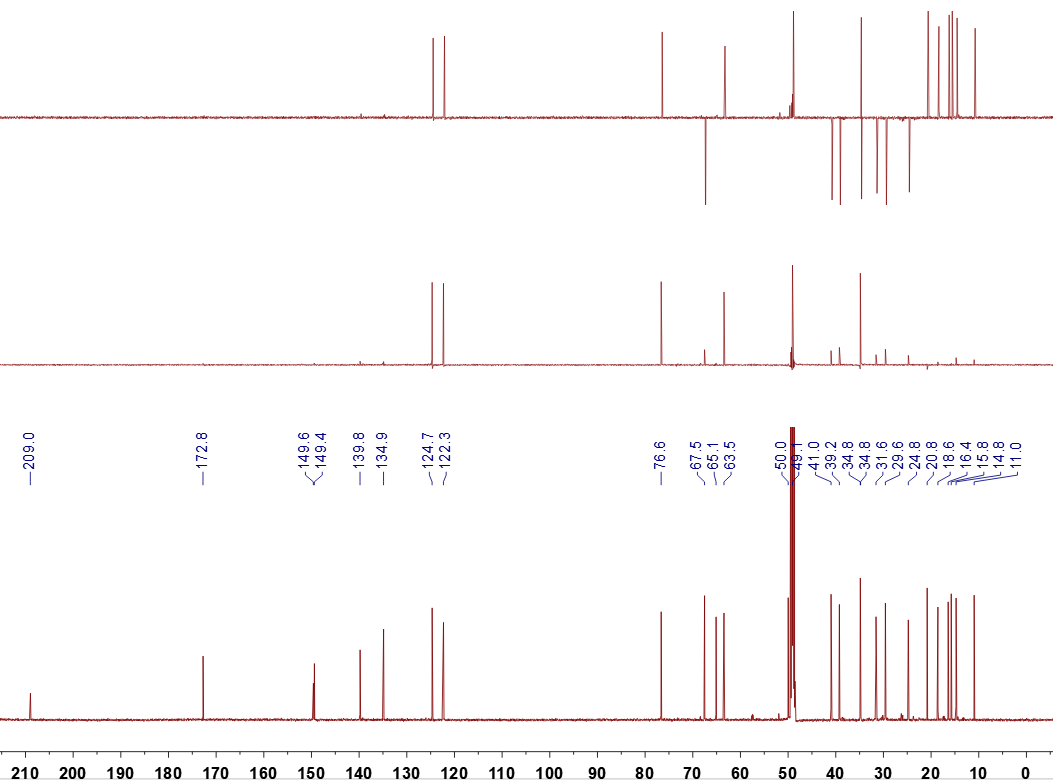


**Fig. S29** ^13^C NMR spectrum of **4.**


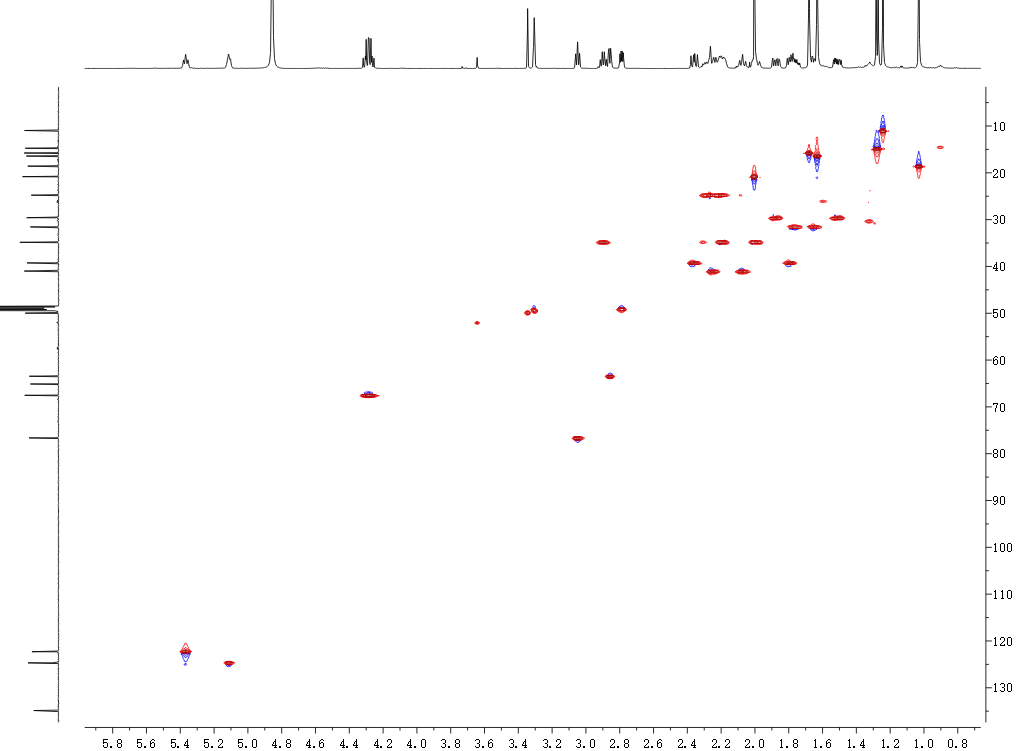


**Fig. S30** HSQC spectrum of **4.**


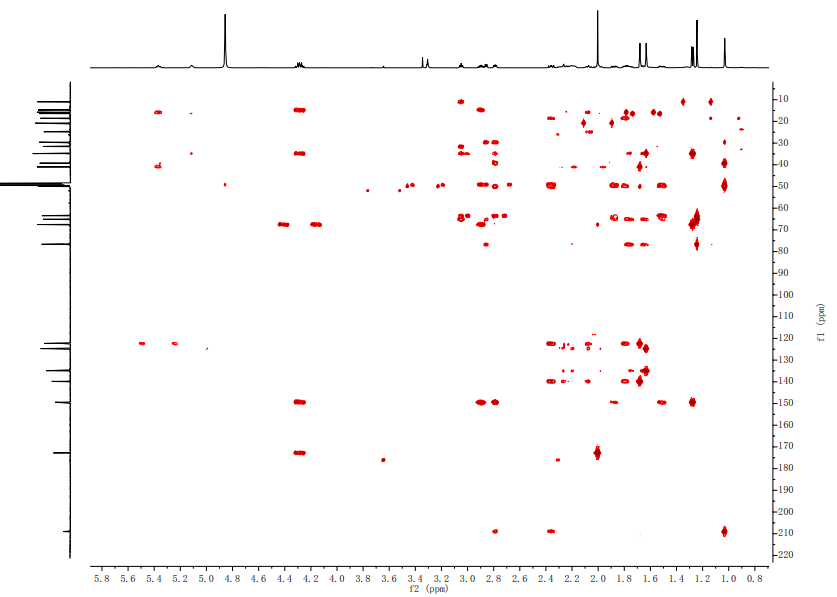


**Fig. S31** HMBC spectrum of **4.**


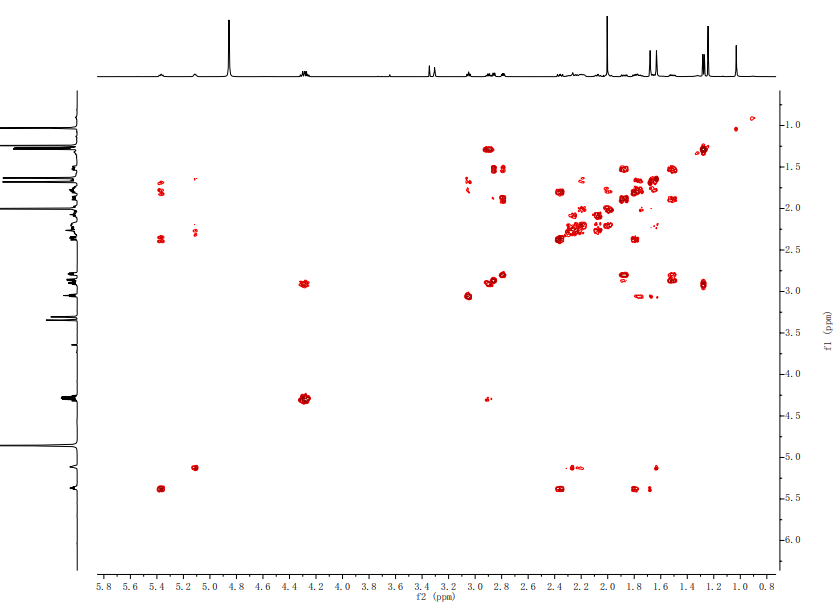


**Fig. S32** ^1^H-^1^H COSY spectrum of **4.**


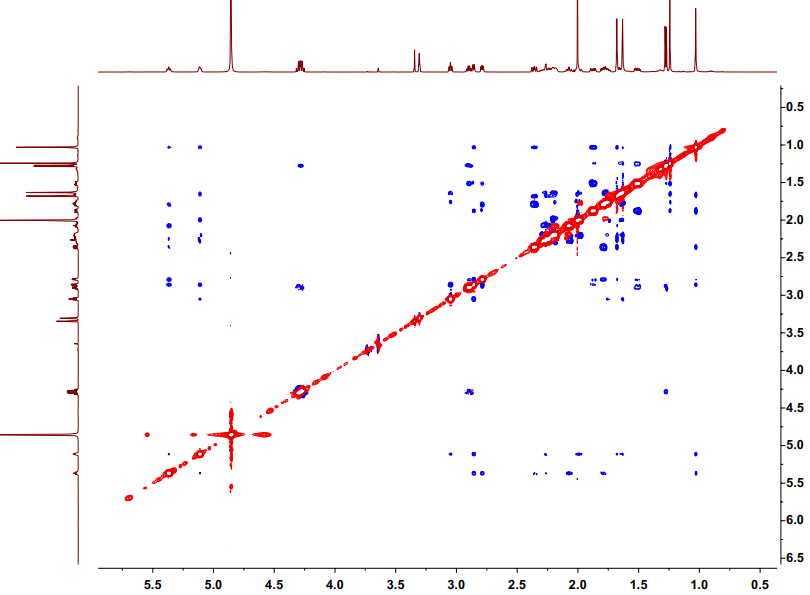


**Fig. S33** ROESY spectrum of **4.**


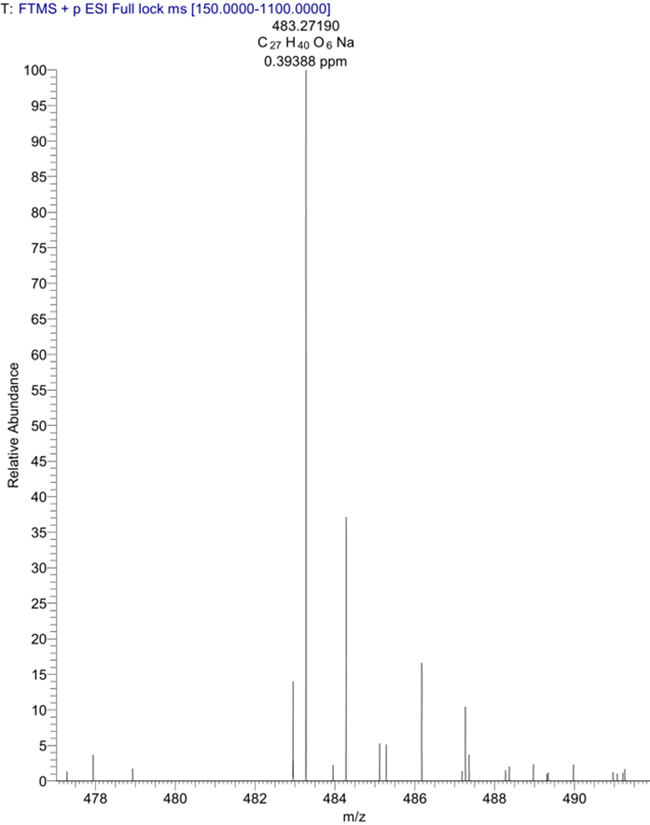


**Fig. S34** HRESIMS spectrum of **4.**


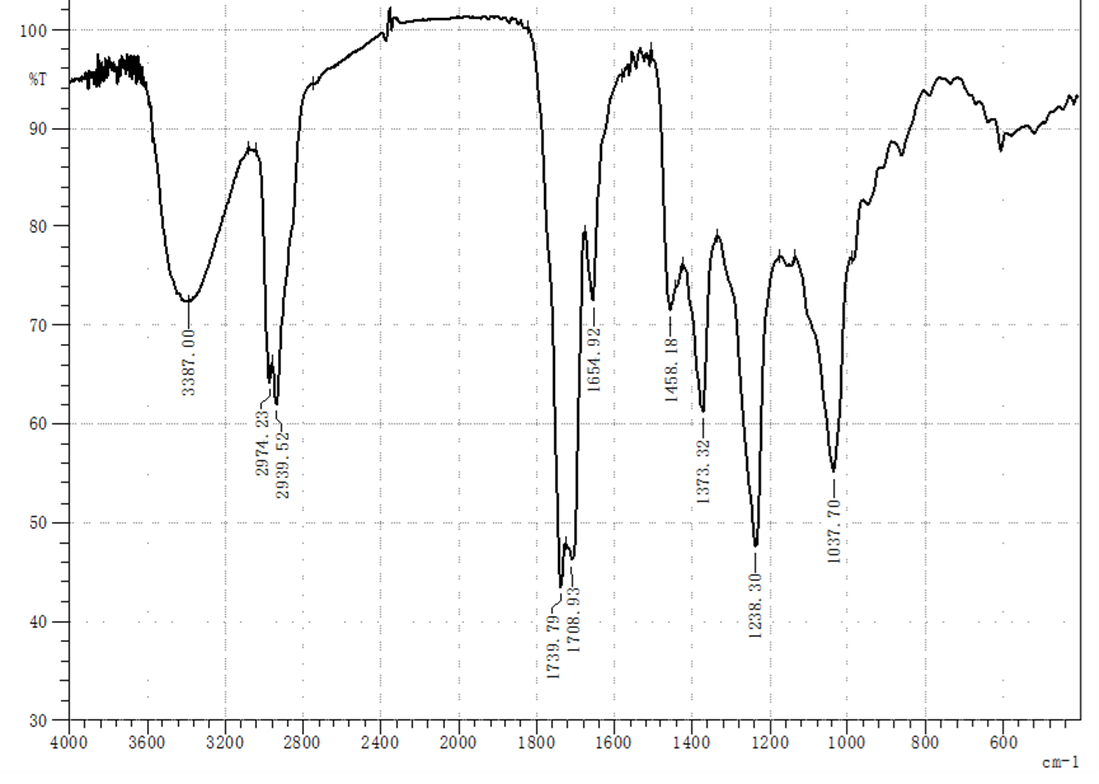


**Fig. S35** IR spectrum of **4.**


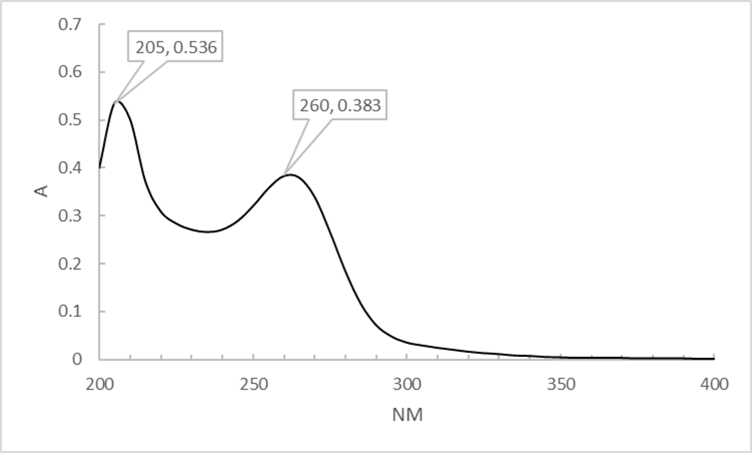


**Fig. S36** UV spectrum of **4.**


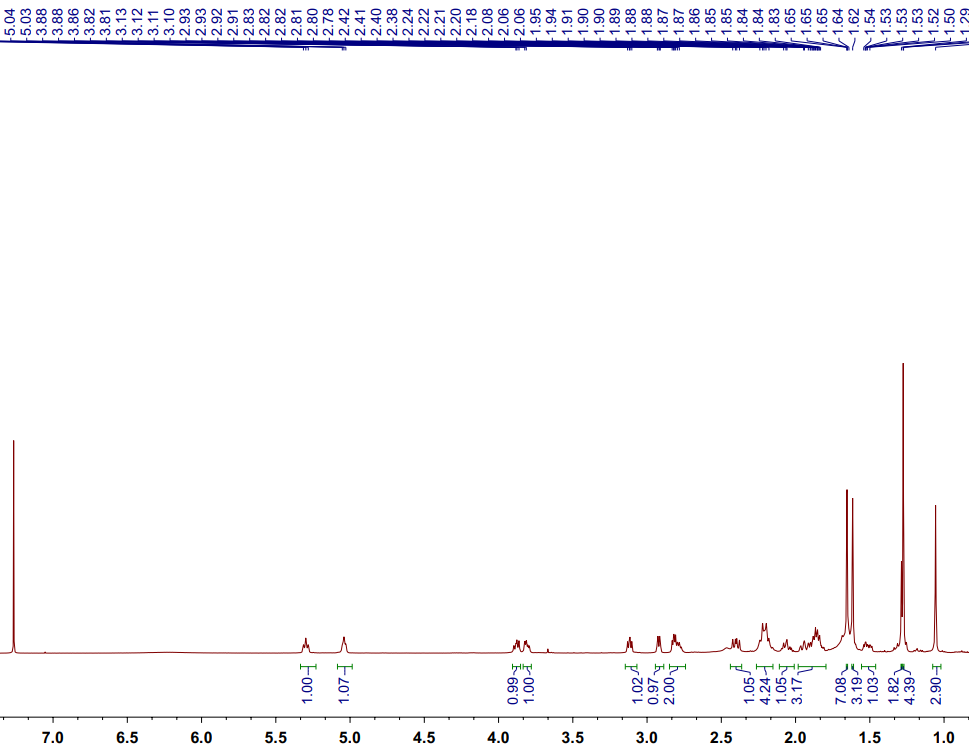


**Fig. S37** ^1^H NMR spectrum of **5.**


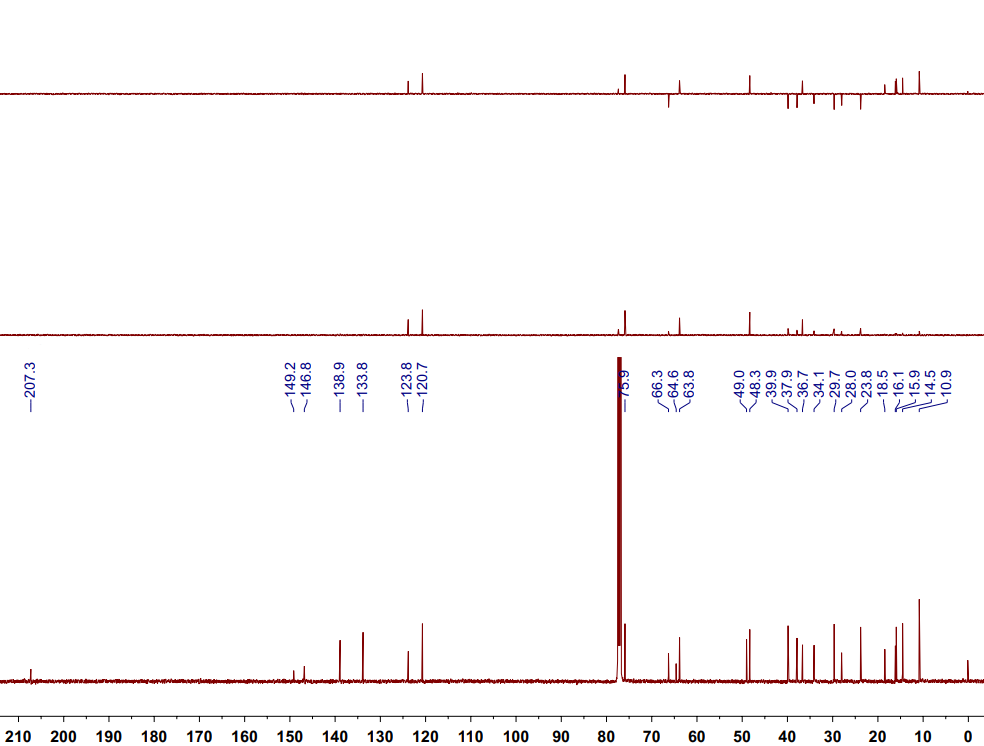


**Fig. S38** ^13^C NMR spectrum of **5.**


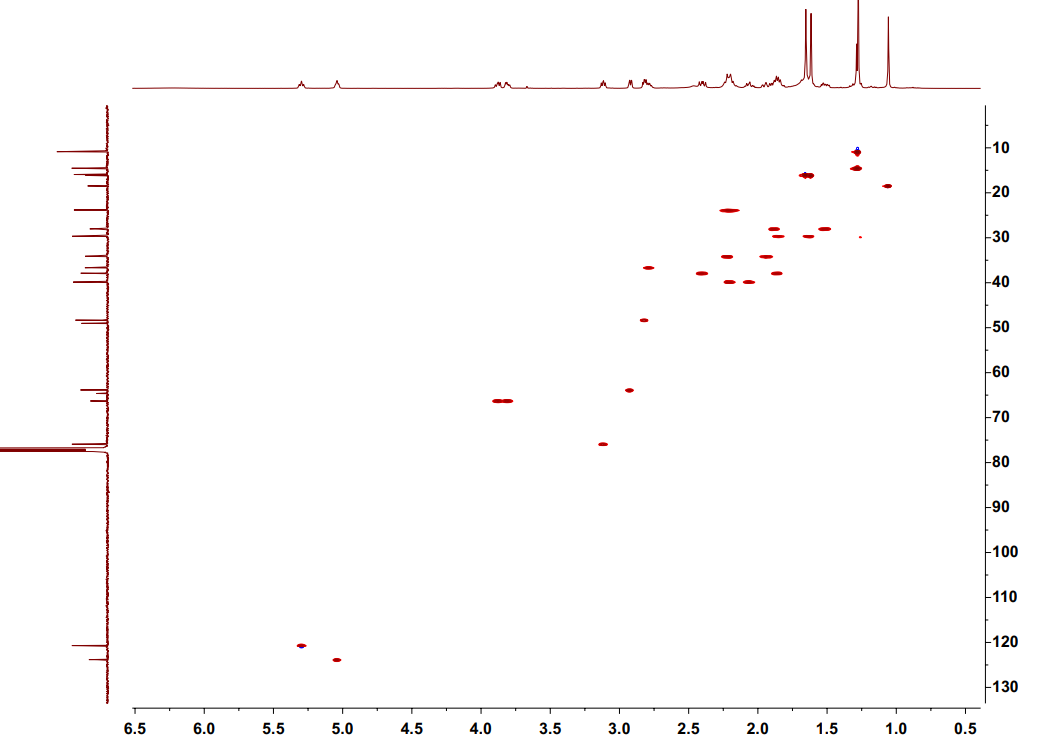


**Fig. S39** HSQC spectrum of **5.**


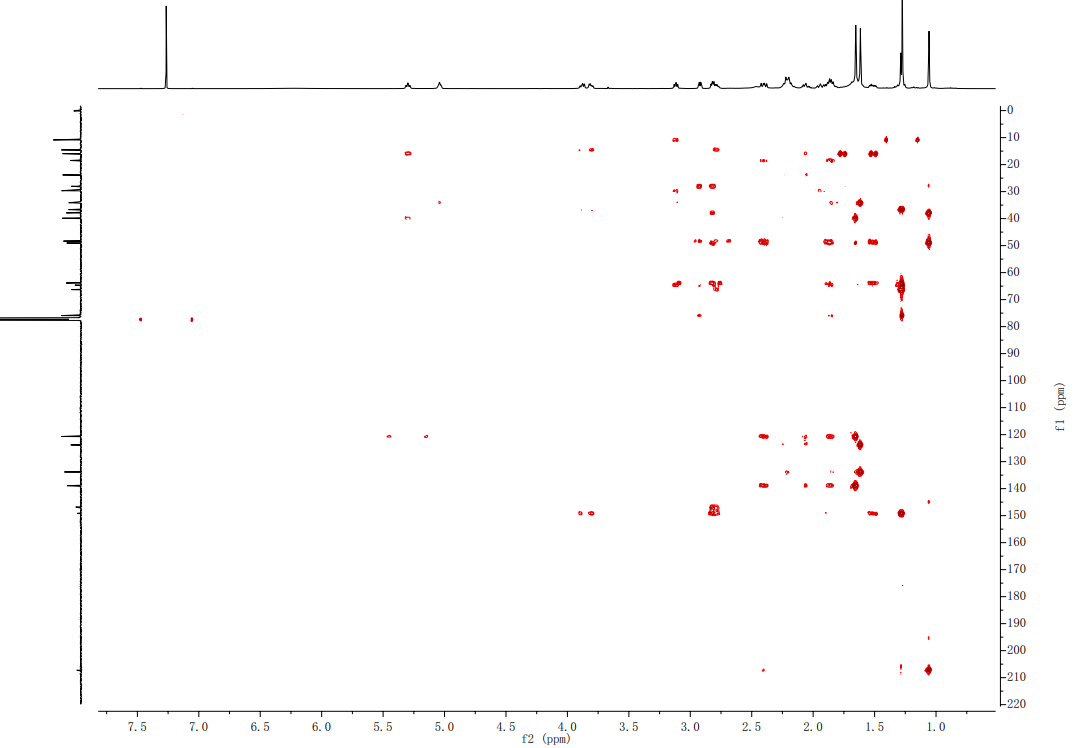


**Fig. S40** HMBC spectrum of **5.**


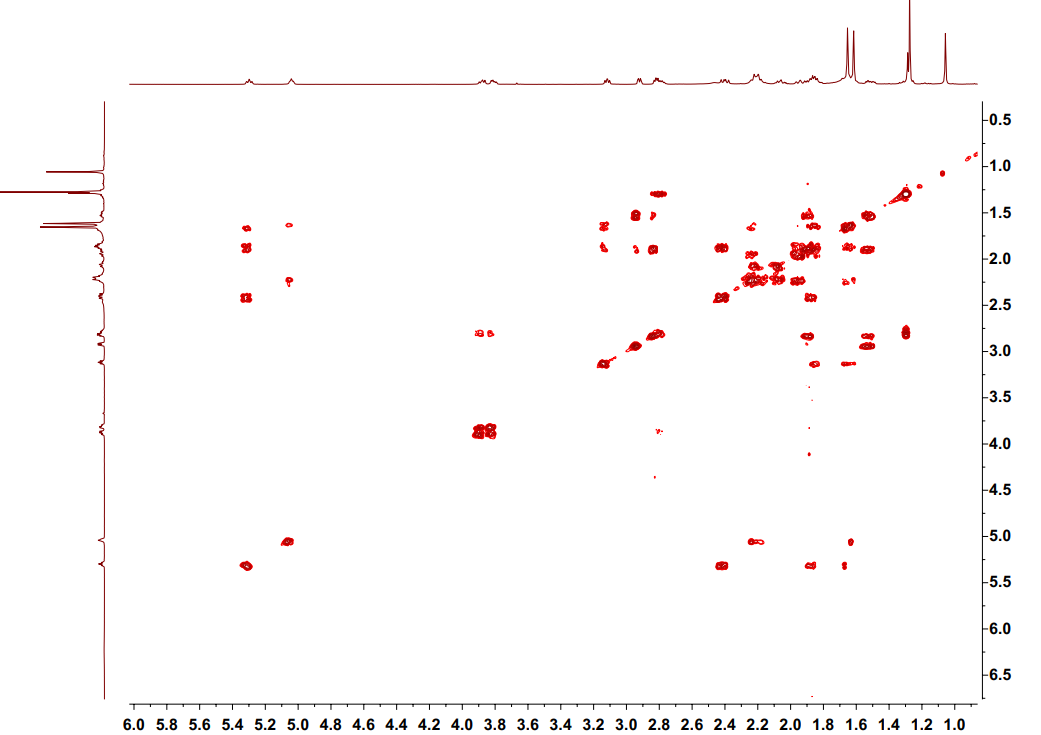


**Fig. S41** ^1^H-^1^H COSY spectrum of **5.**


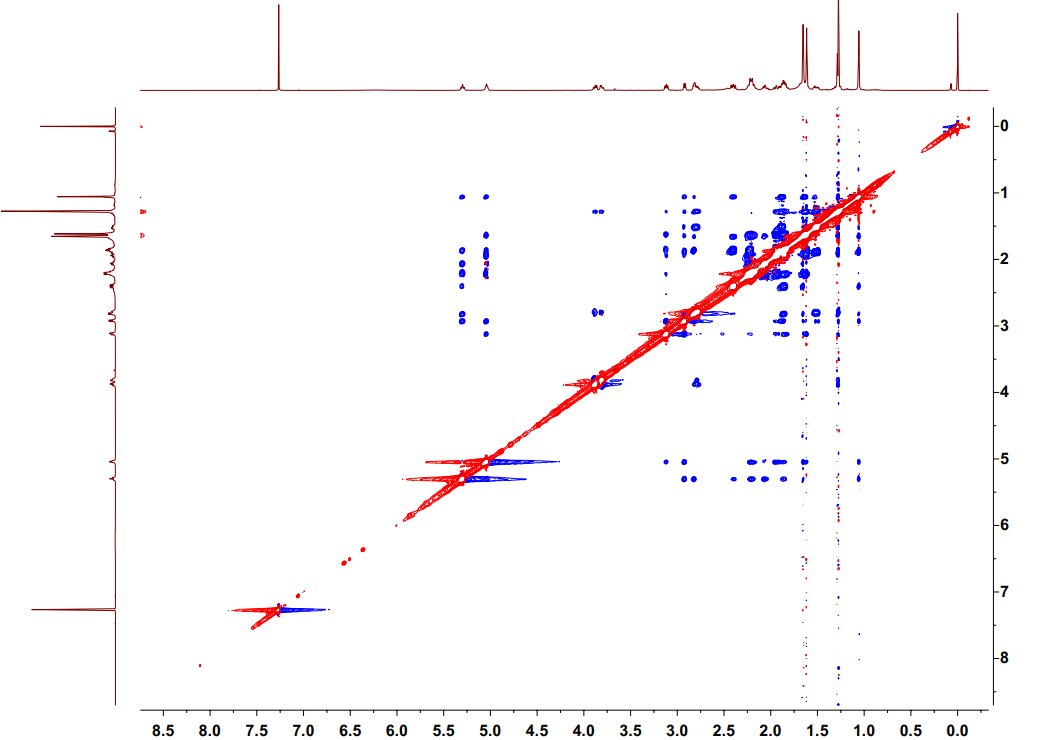


**Fig. S42** ROESY spectrum of **5.**


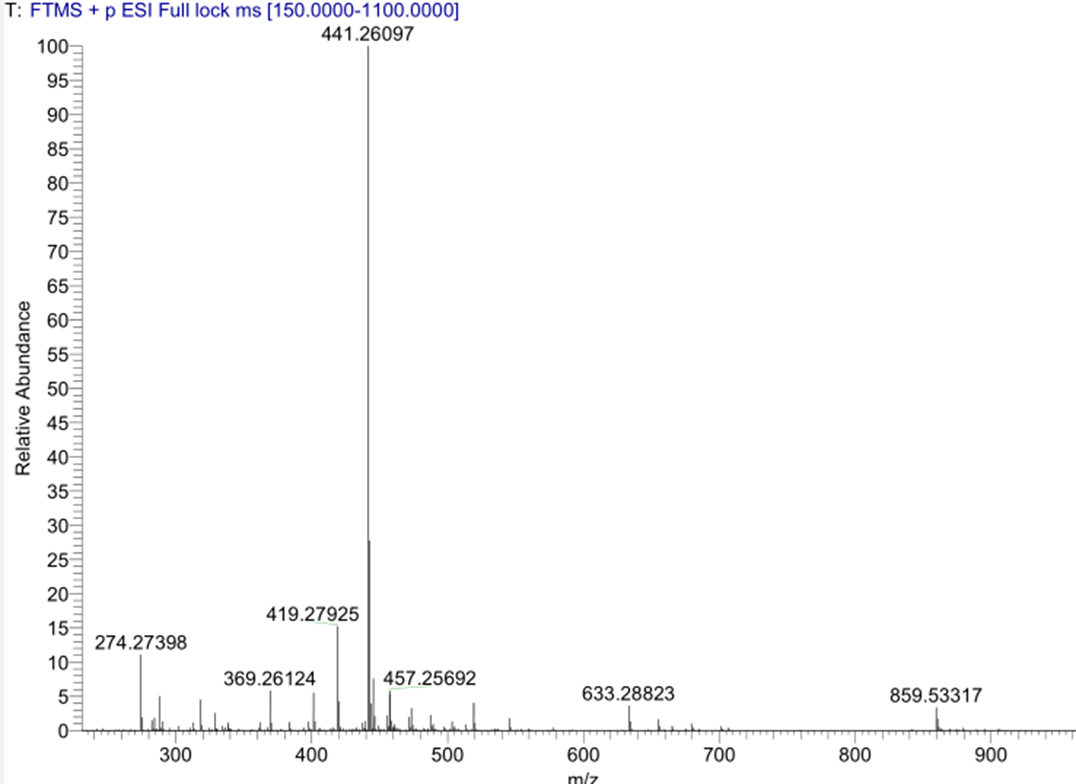


**Fig. S43** HRESIMS spectrum of **5.**


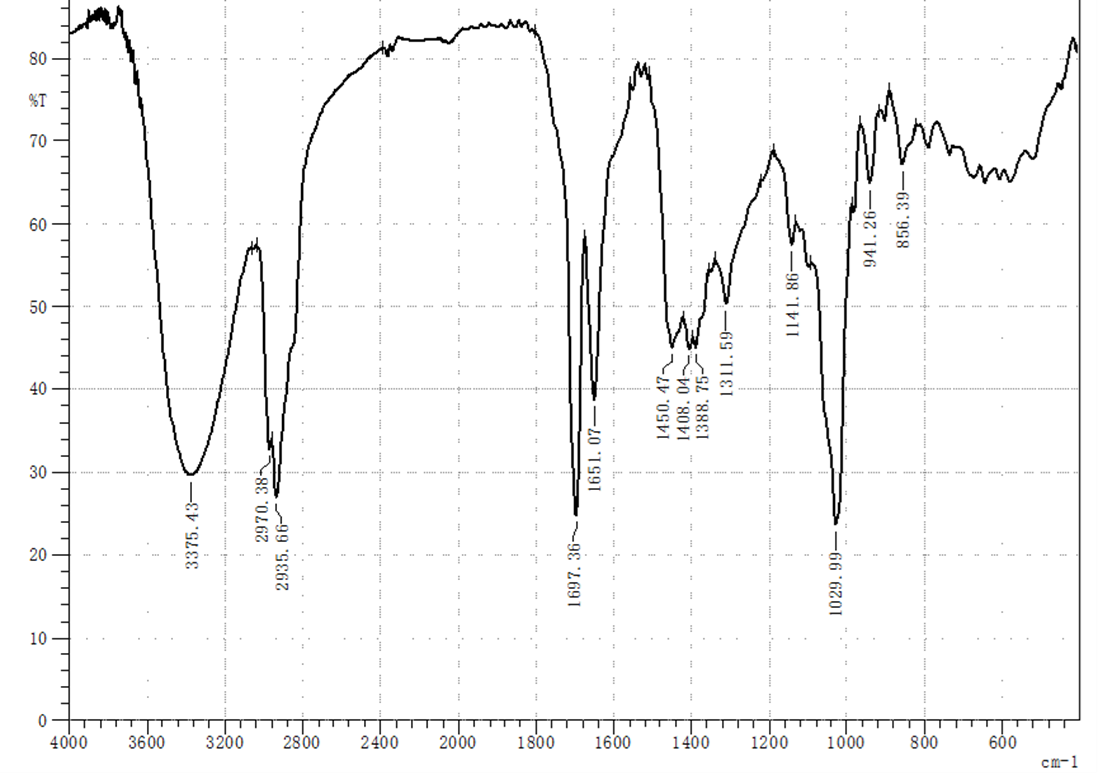


**Fig. S44** IR spectrum of **5.**


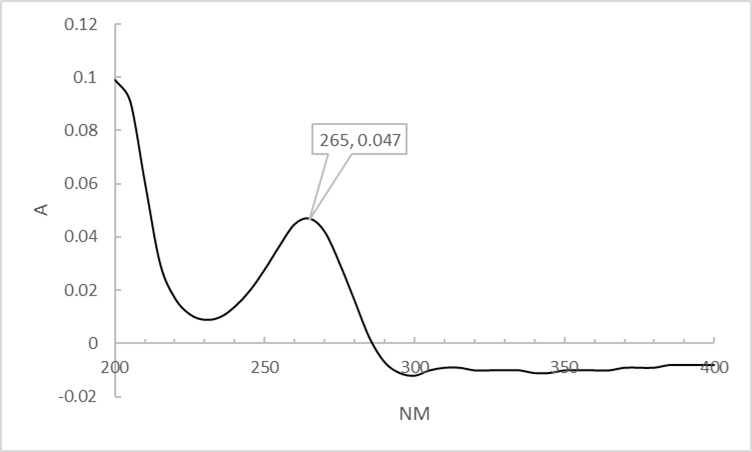


**Fig. S45** UV spectrum of **5.**


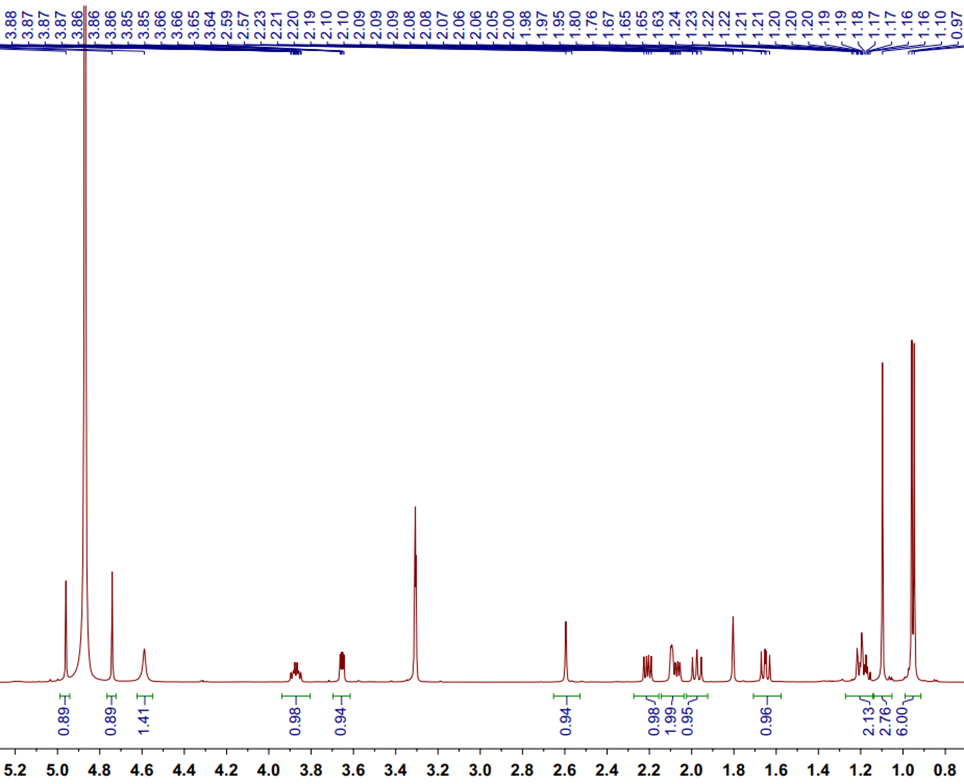


**Fig. S46** ^1^H NMR spectrum of **6.**


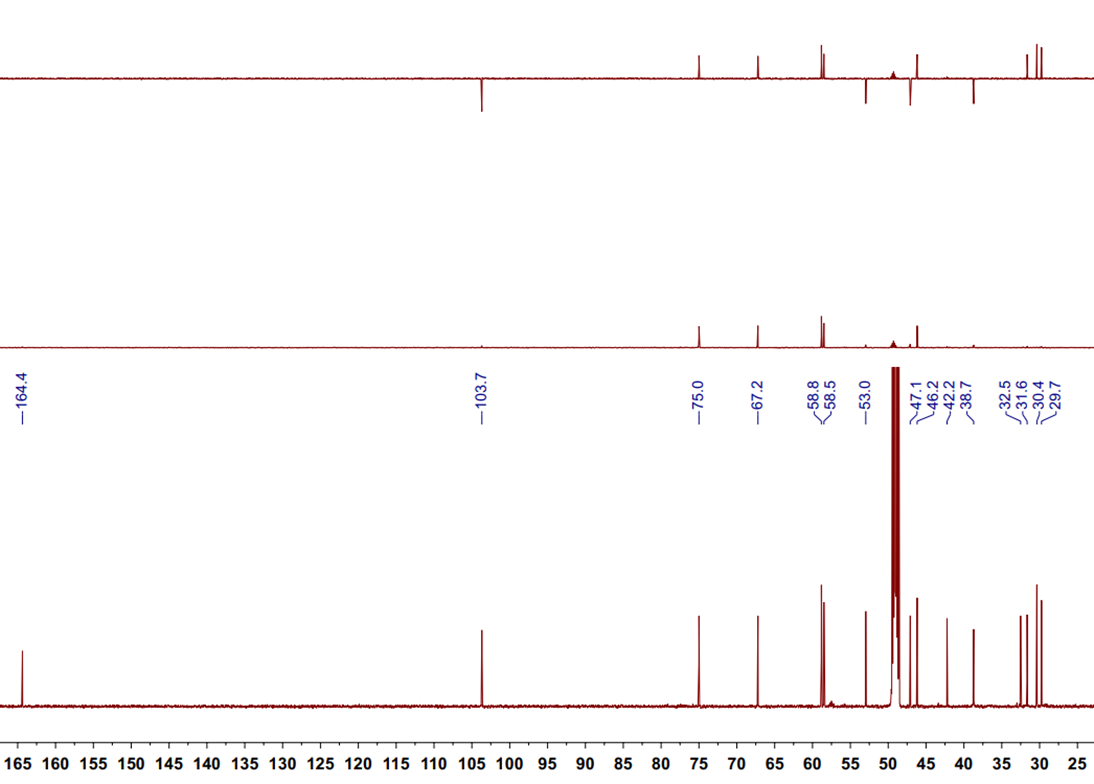


**Fig. S47** ^13^C NMR spectrum of **6.**


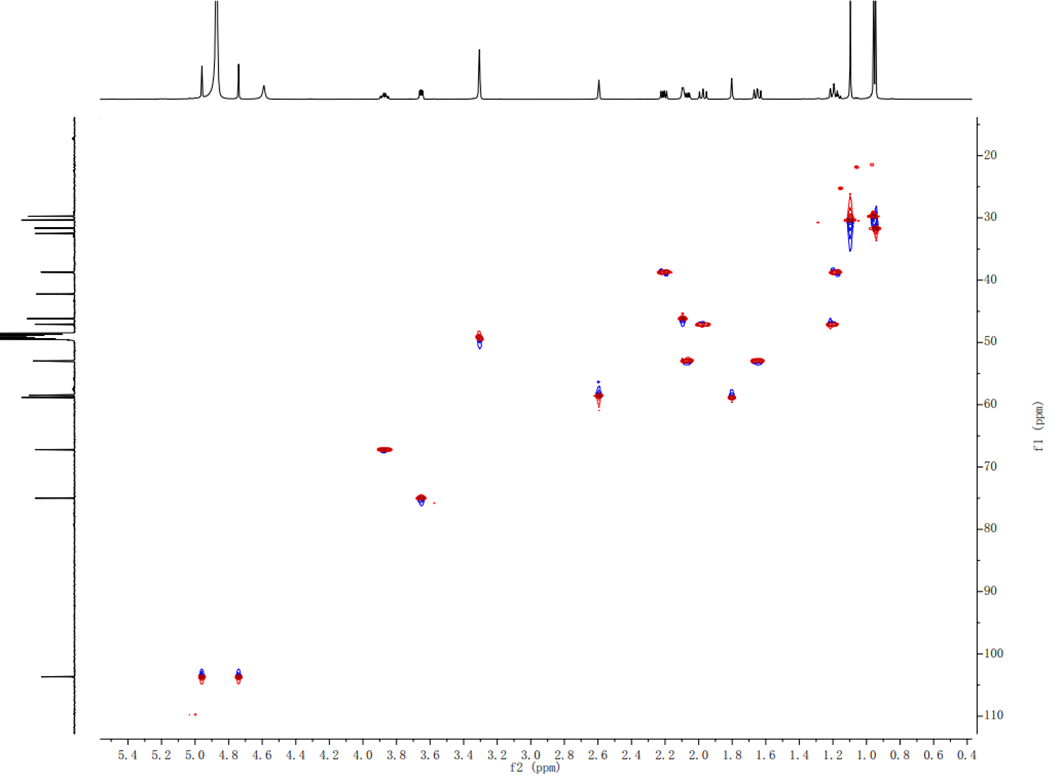


**Fig. S48** HSQC spectrum of **6.**


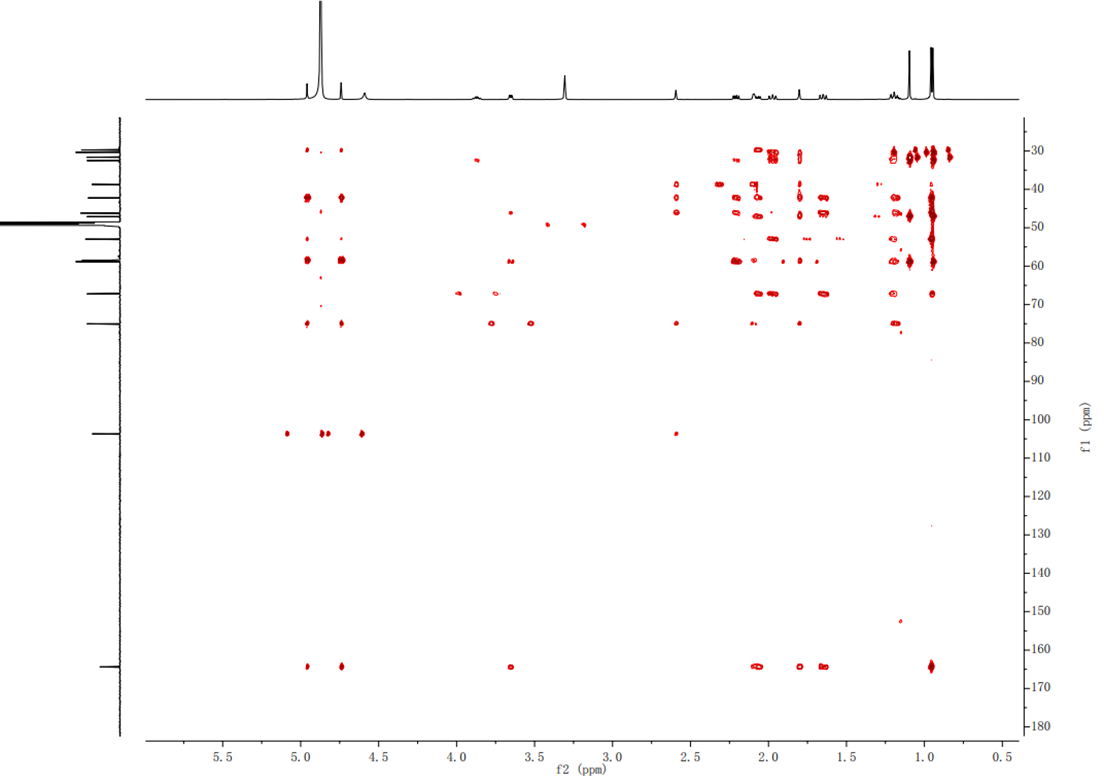


**Fig. S49** HMBC spectrum of **6.**


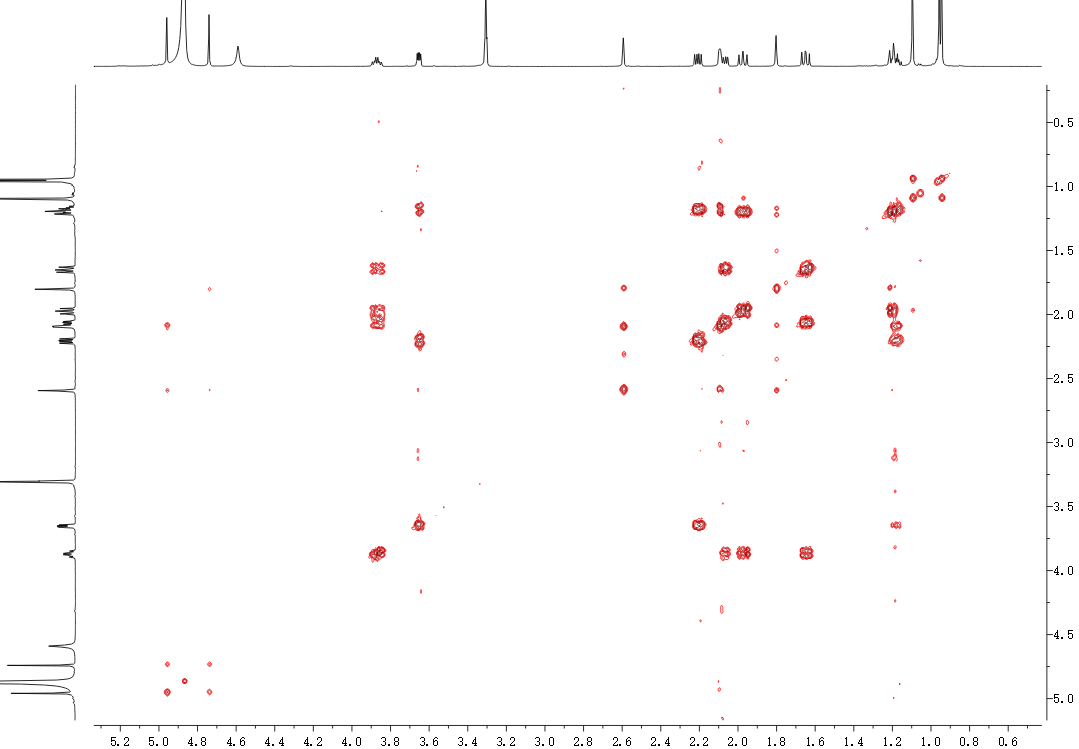


**Fig. S50** ^1^H-^1^H COSY spectrum of **6.**


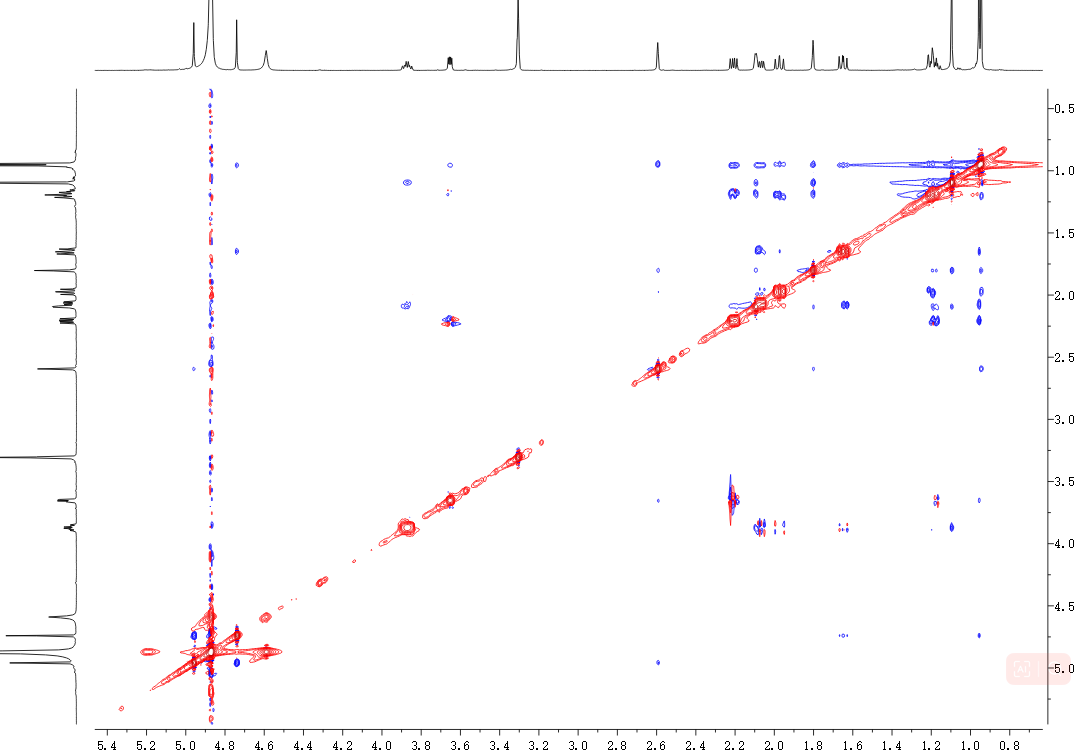


**Fig. S51** ROESY spectrum of **6.**


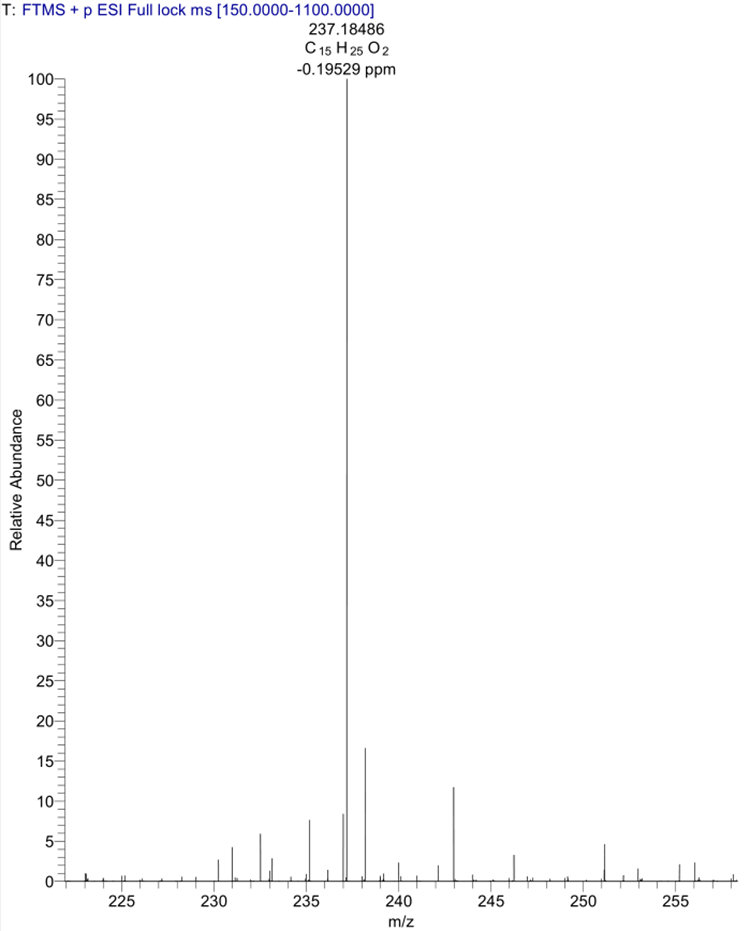


**Fig. S52** HRESIMS spectrum of **6.**


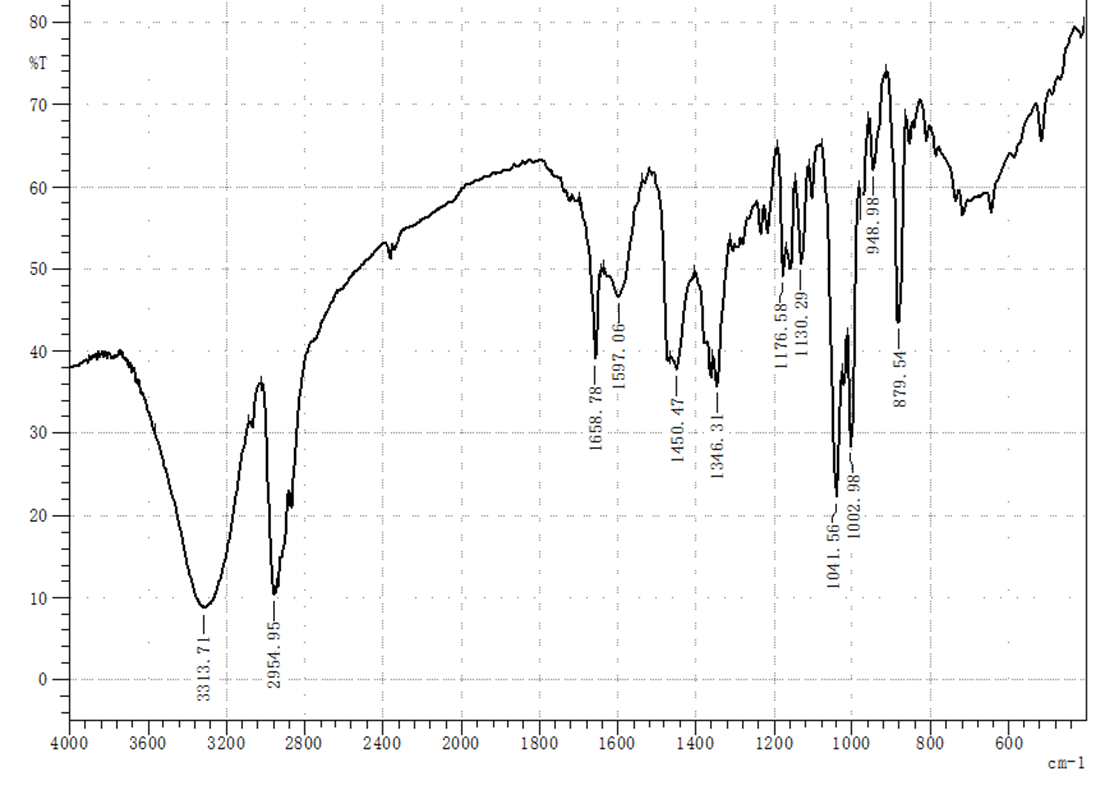


**Fig. S53** IR spectrum of **6.**


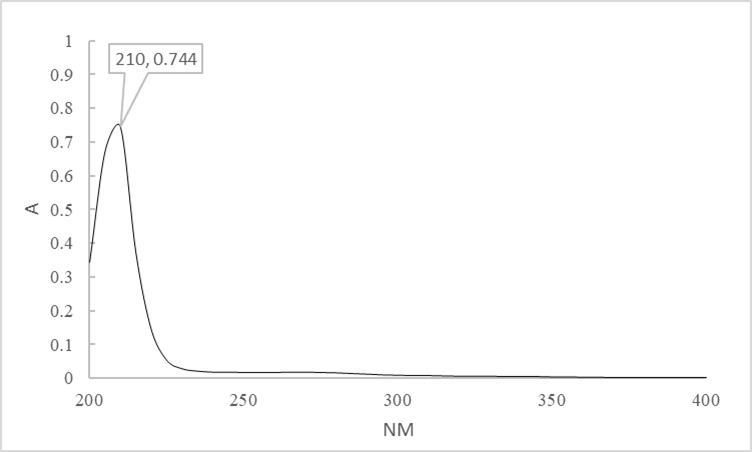


**Fig. S54** UV spectrum of **6.**


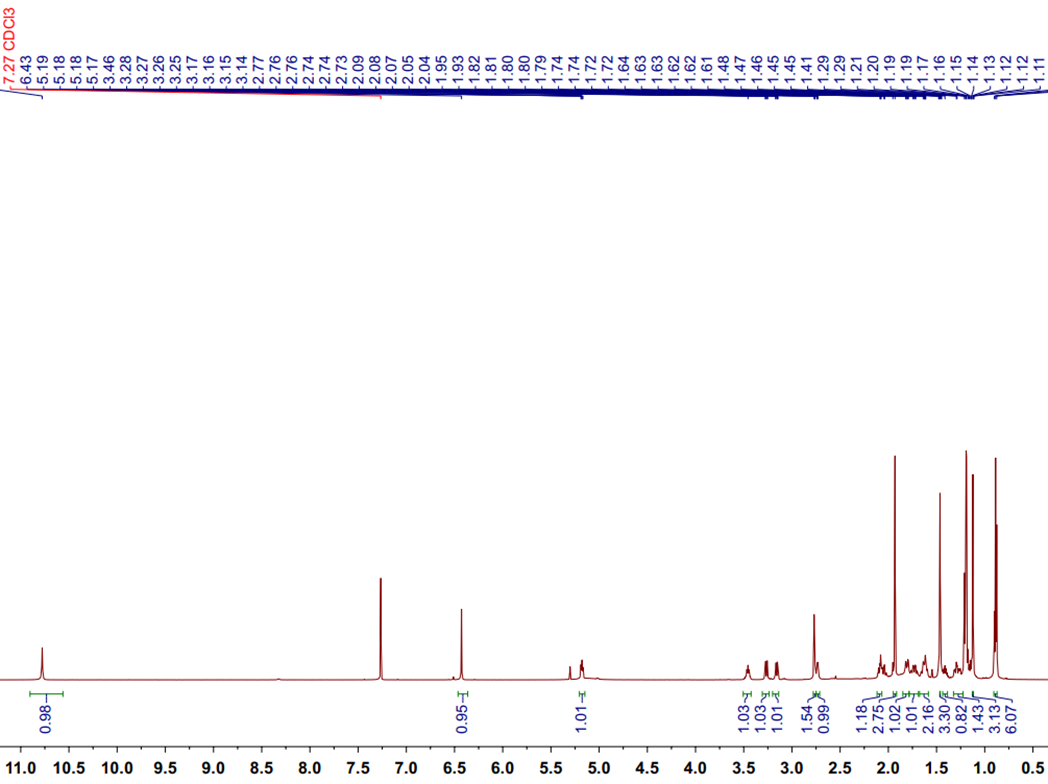


**Fig. S55** ^1^H NMR spectrum of **7.**


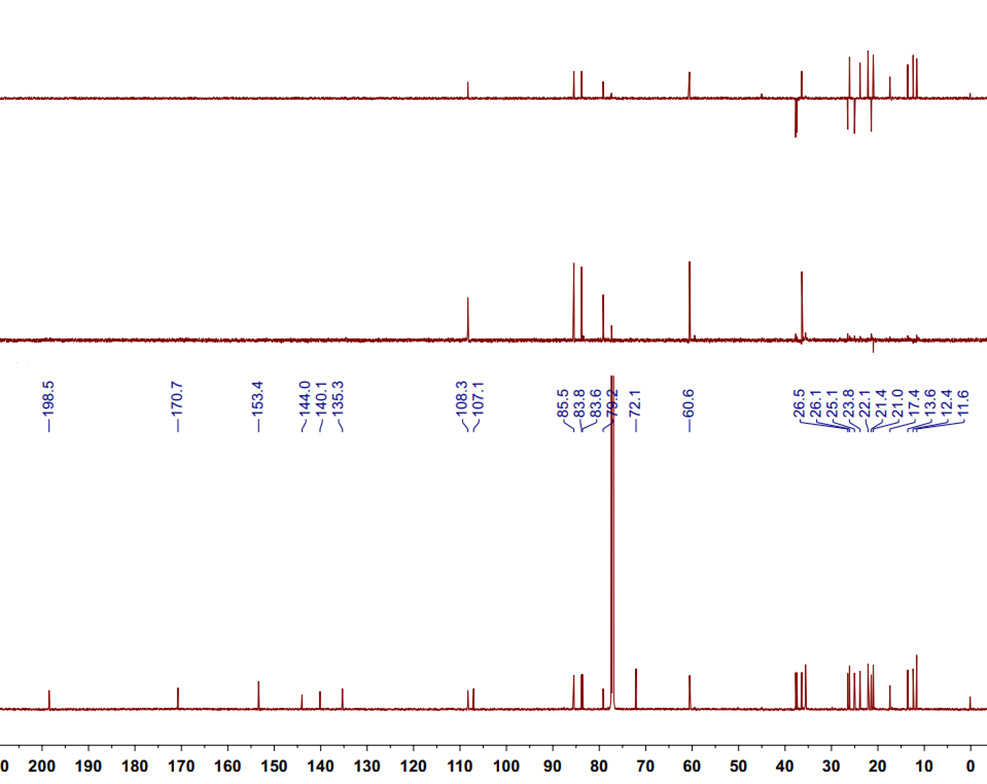


**Fig. S56** ^13^C NMR spectrum of **7.**


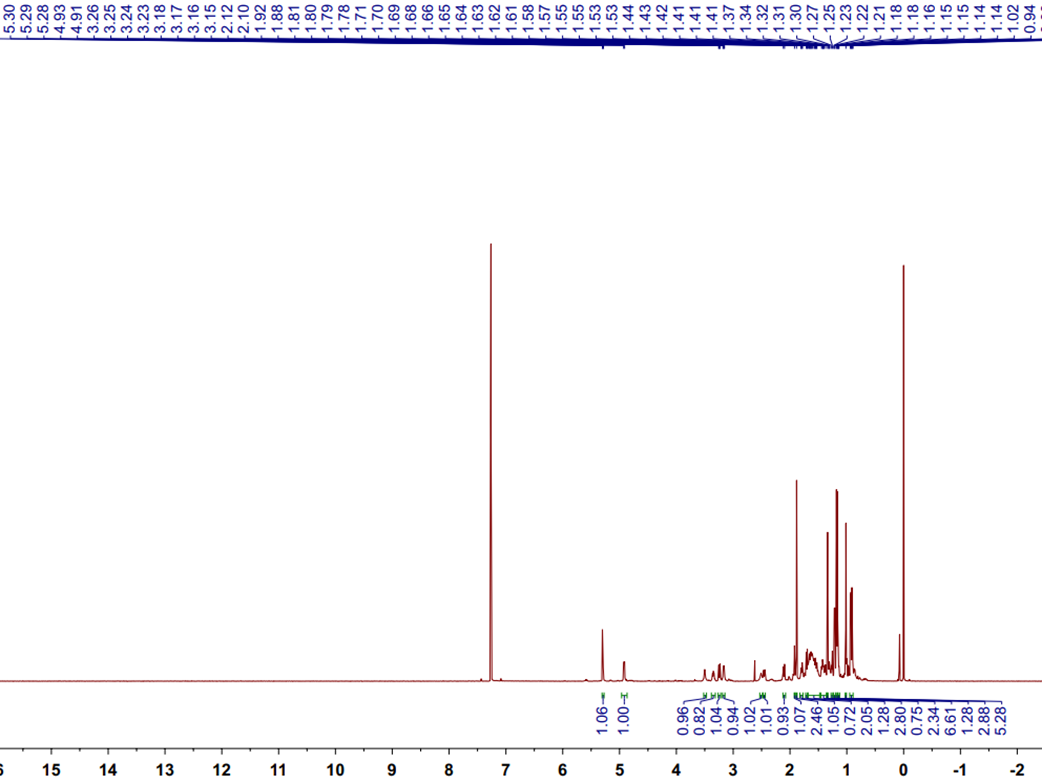


**Fig. S57** ^1^H NMR spectrum of **8.**


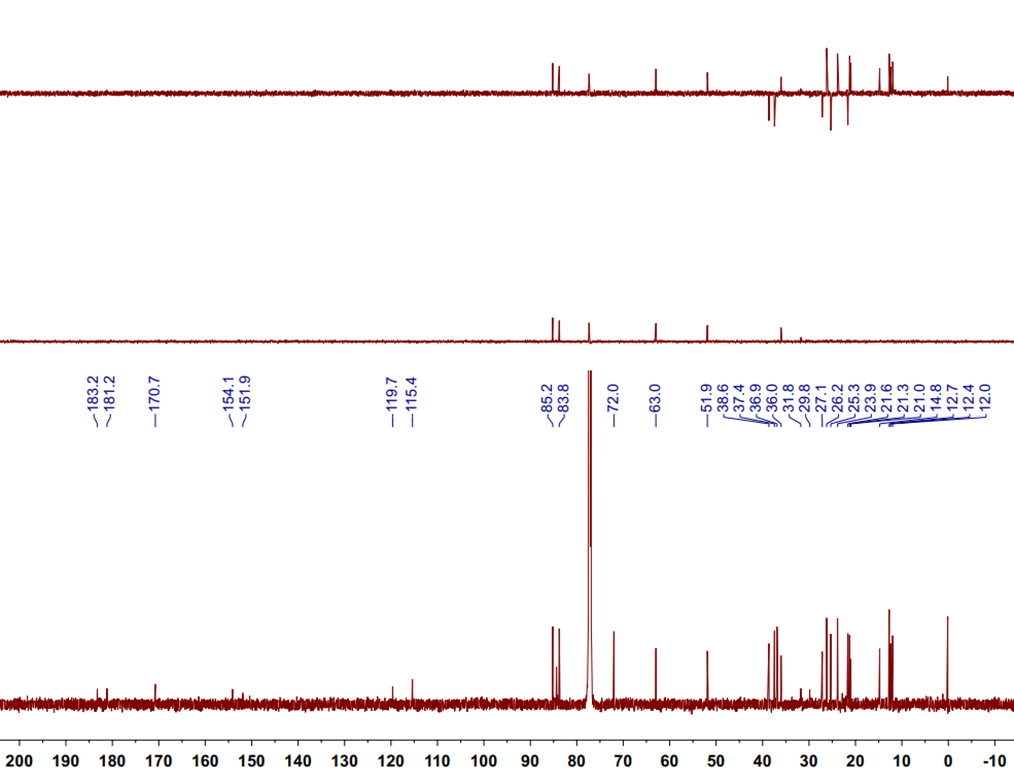


**Fig. S58** ^13^C NMR spectrum of **8.**


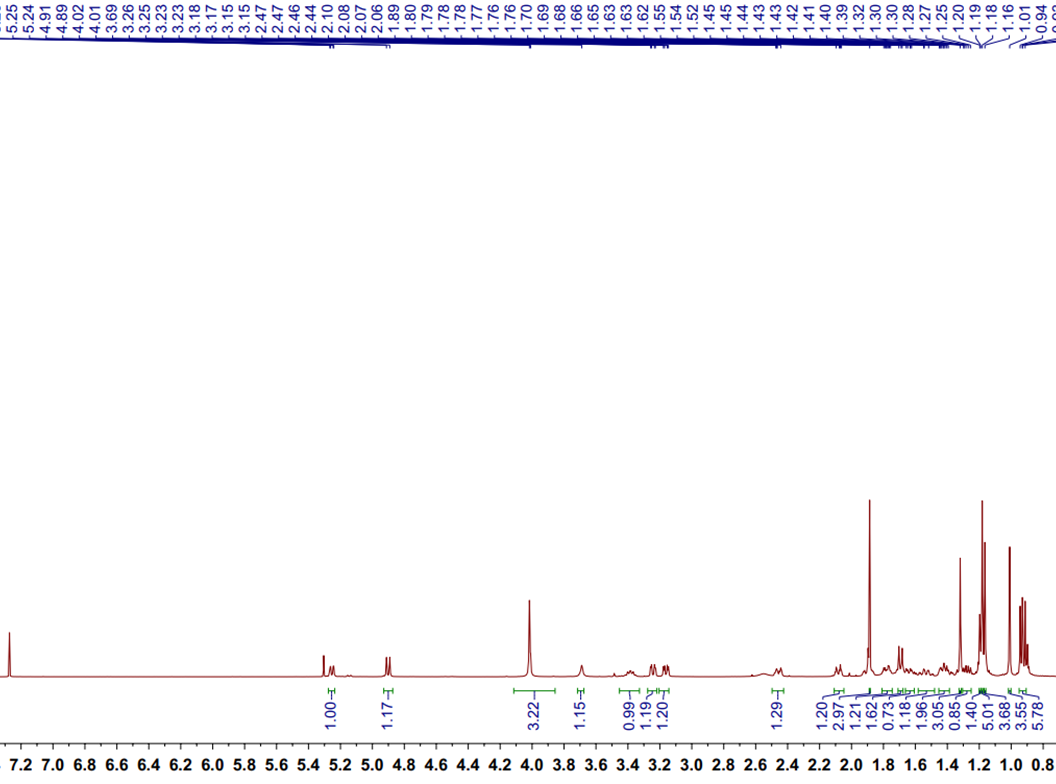


**Fig. S59** ^1^H NMR spectrum of **9.**


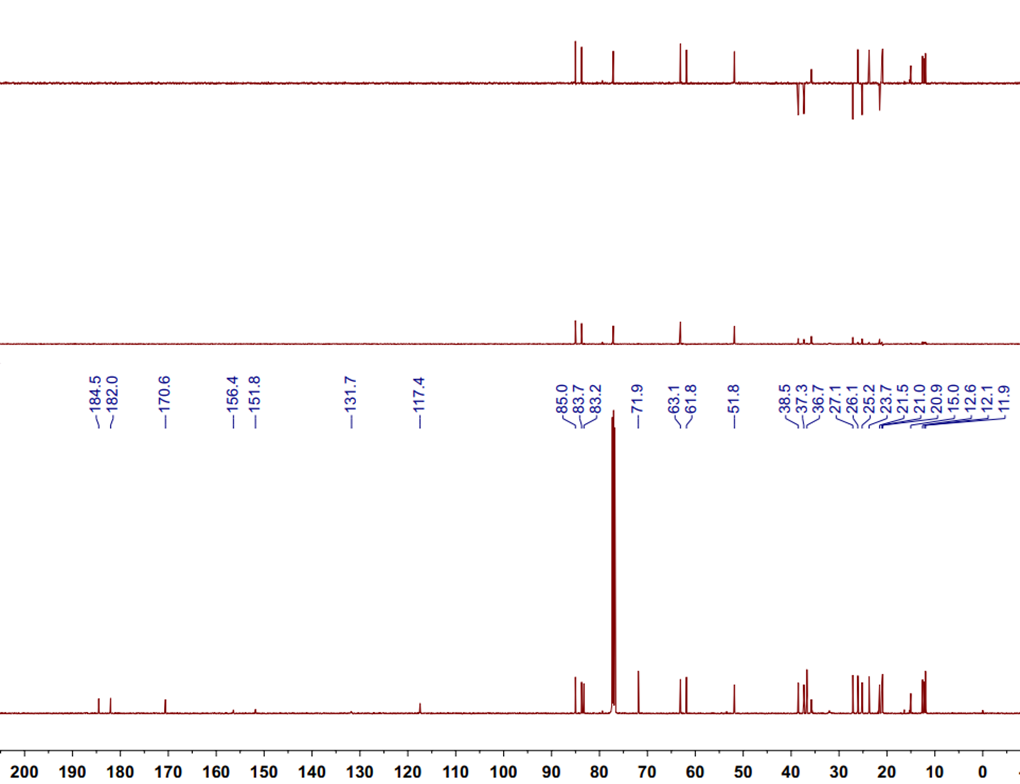


**Fig. S60** ^13^C NMR spectrum of **9.**


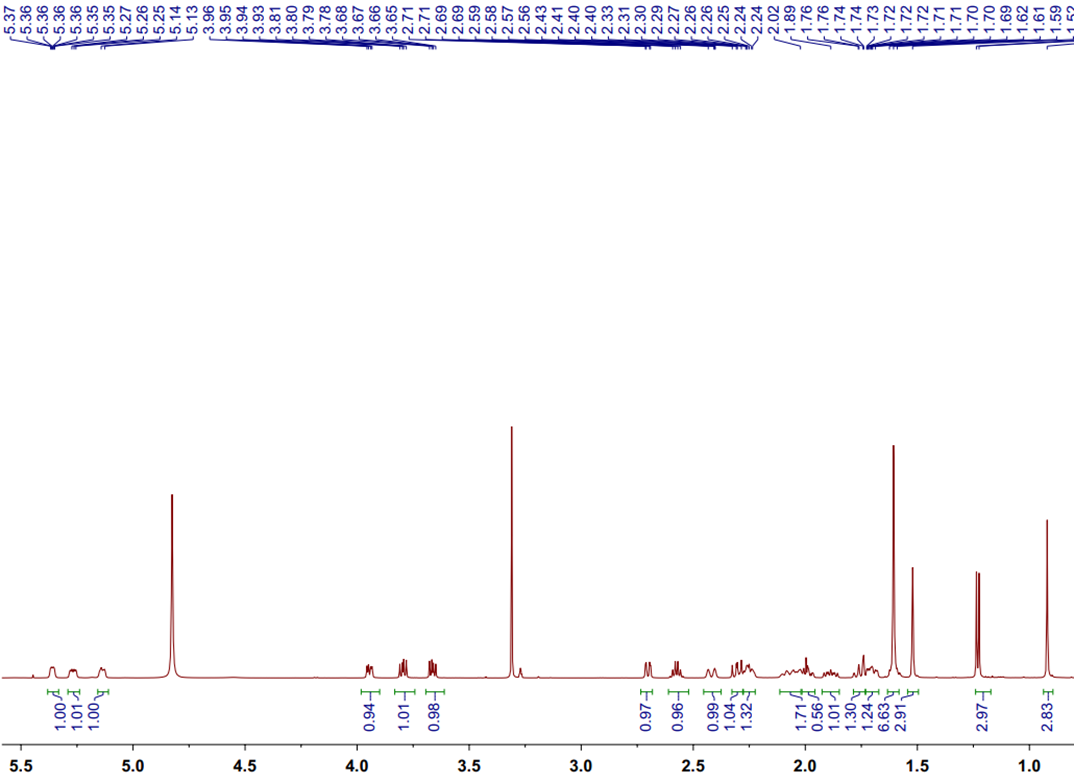


**Fig. S61** ^1^H NMR spectrum of **10.**


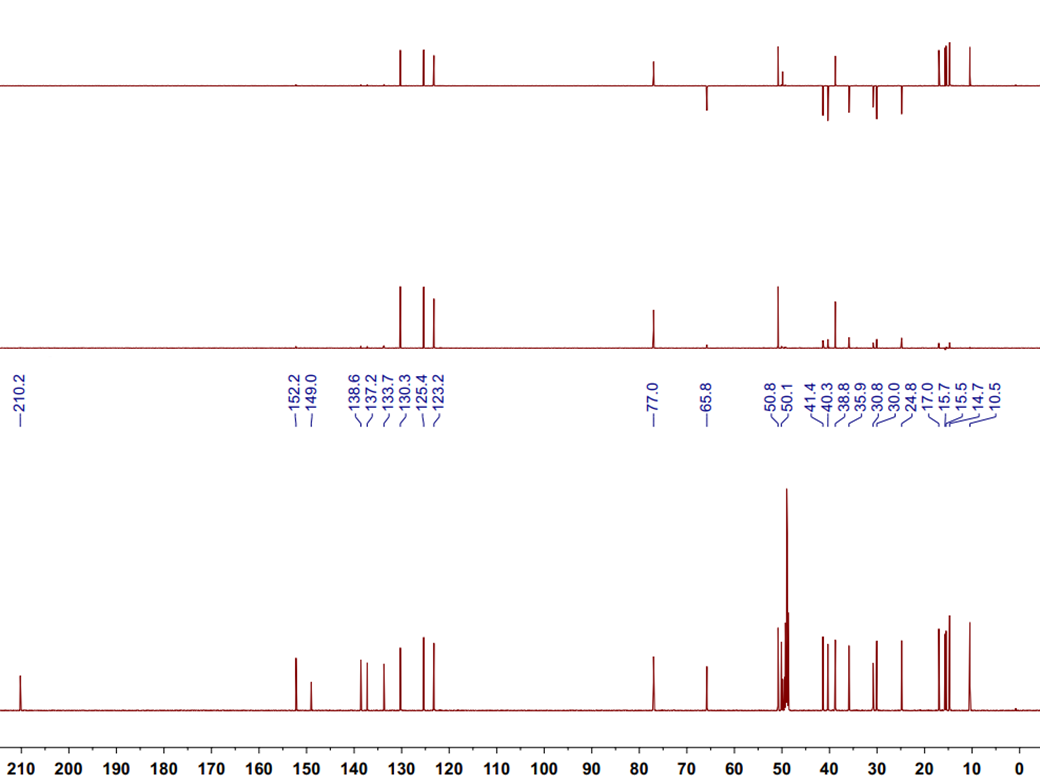


**Fig. S62** ^13^C NMR spectrum of **10.**


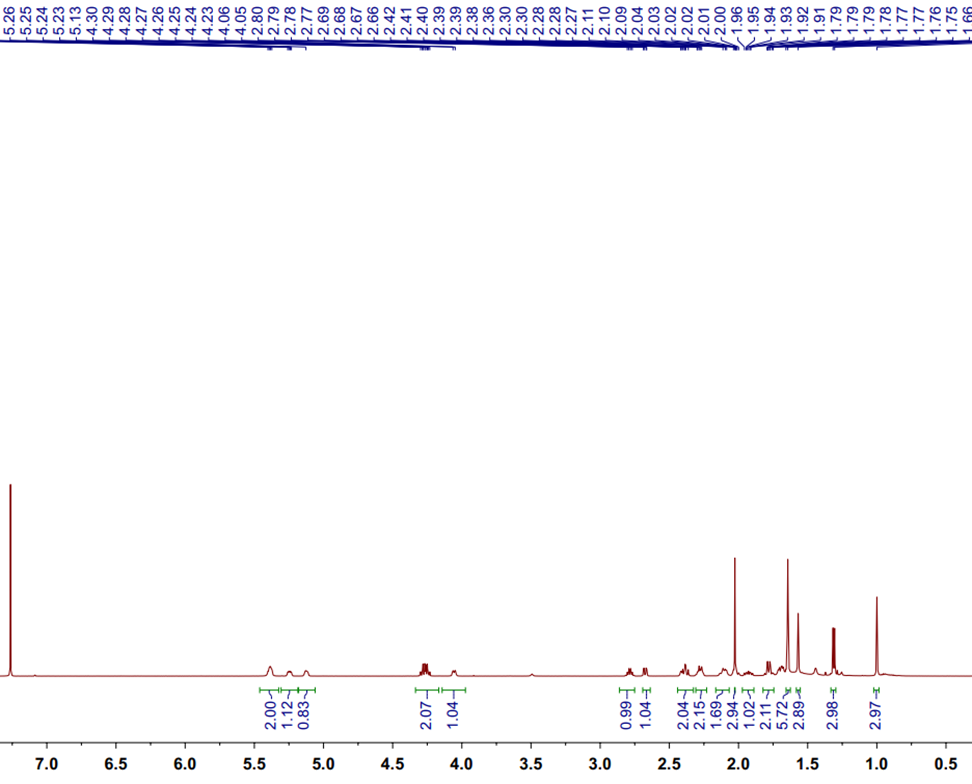


**Fig. S63** ^1^H NMR spectrum of **11.**


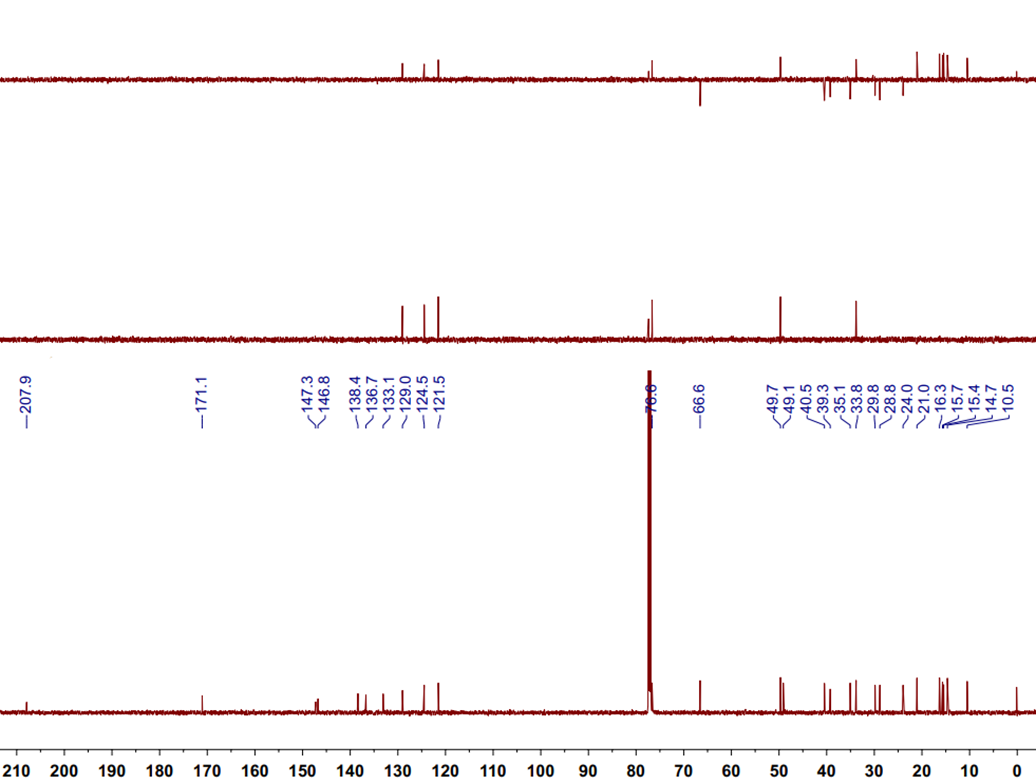


**Fig. S64** ^13^C NMR spectrum of **11.**


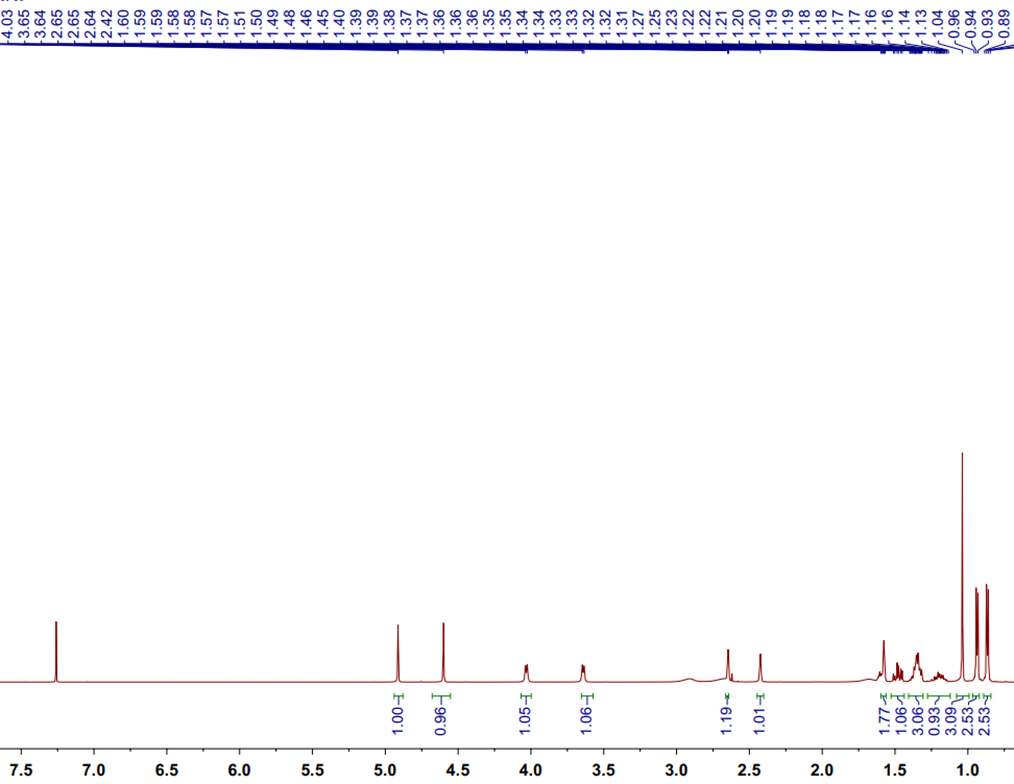


**Fig. S65** ^1^H NMR spectrum of **12.**


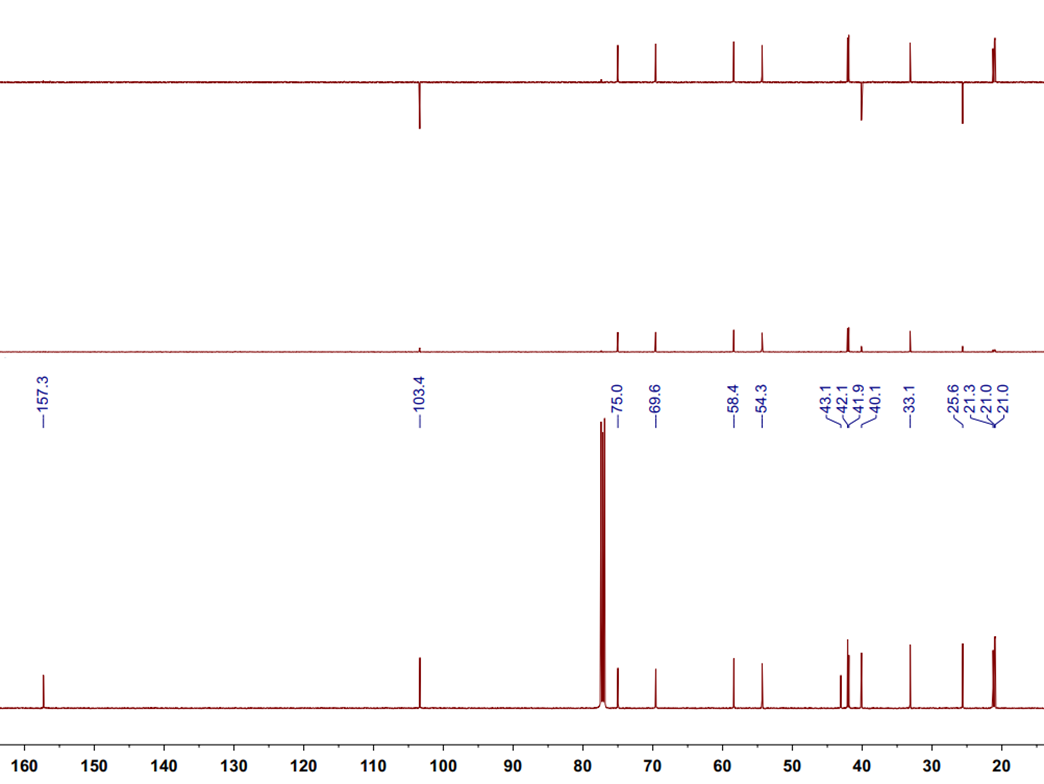


**Fig. S66** ^13^C NMR spectrum of **12.**


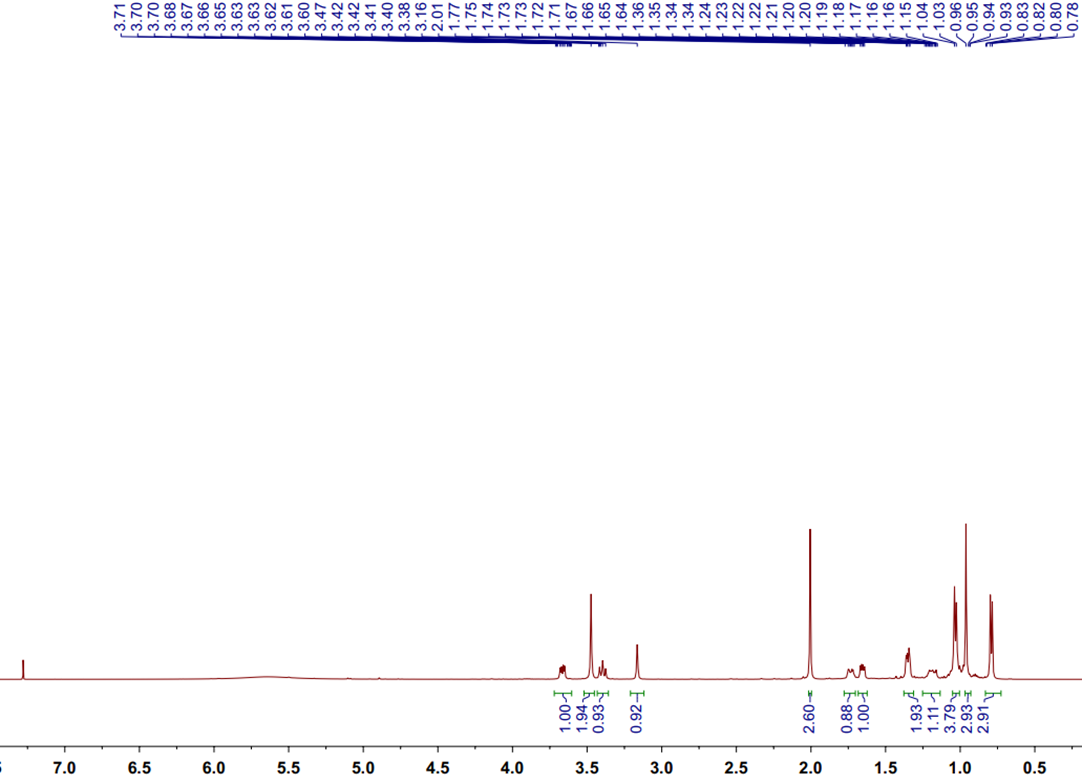


**Fig. S67** ^1^H NMR spectrum of **13.**


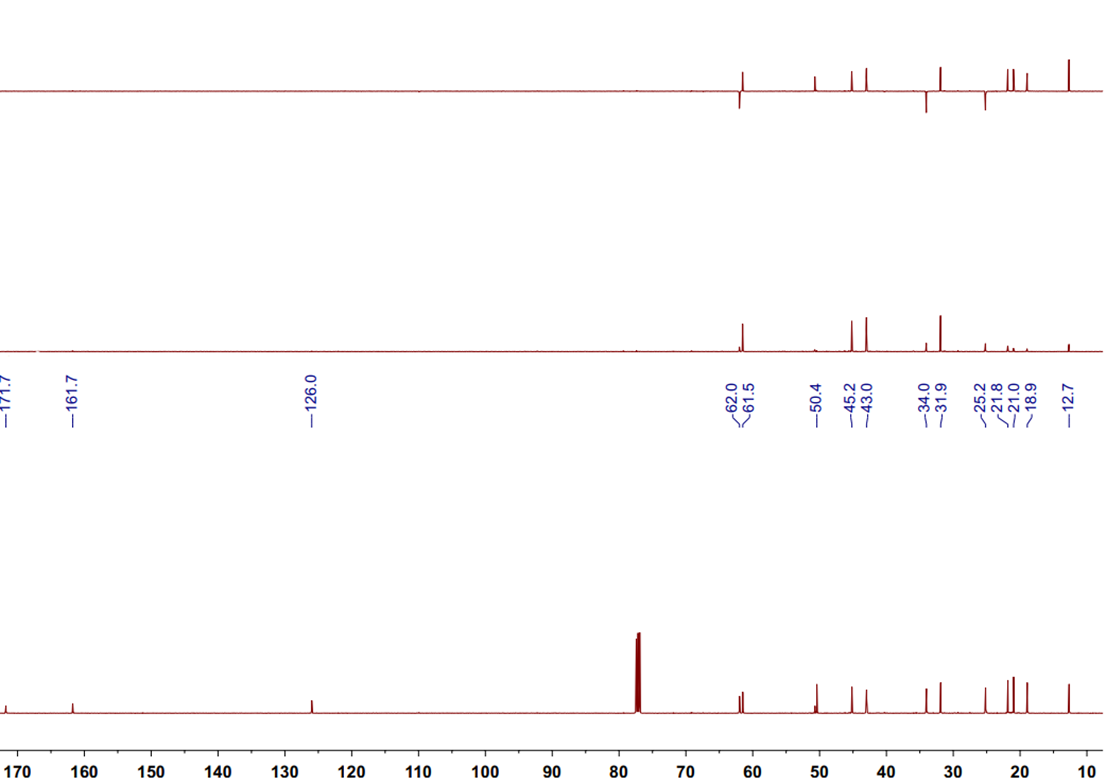


**Fig. S68** ^13^C NMR spectrum of **13.**


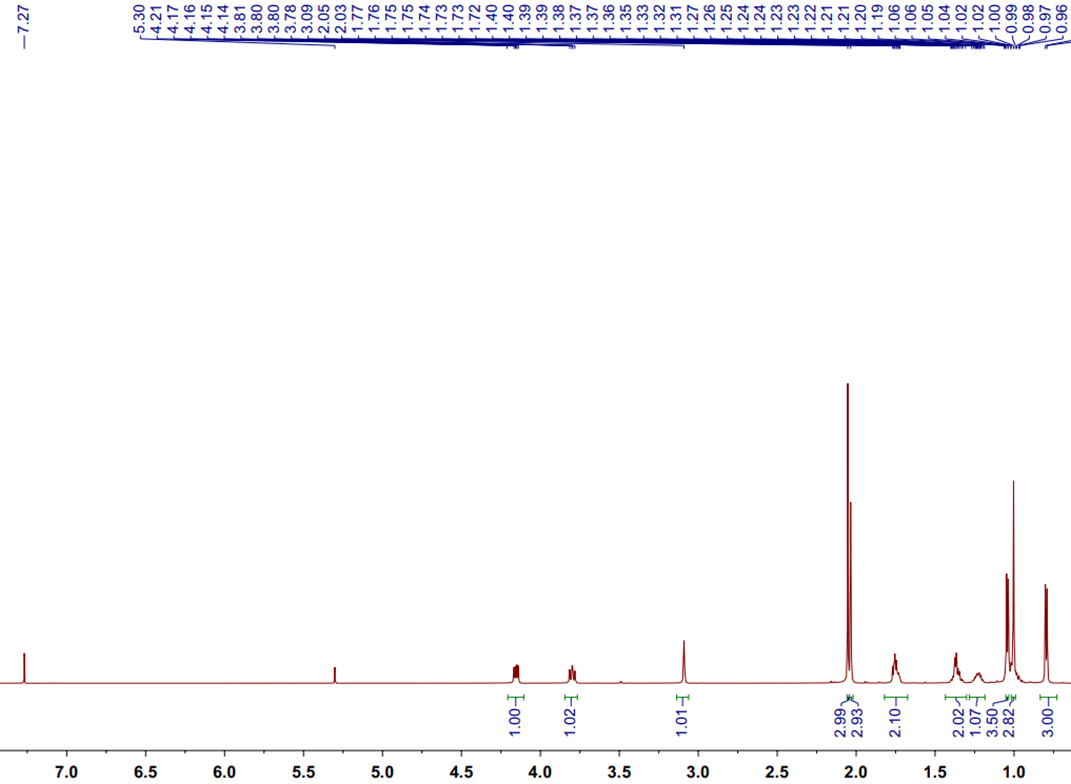


**Fig. S69** ^1^H NMR spectrum of **14.**


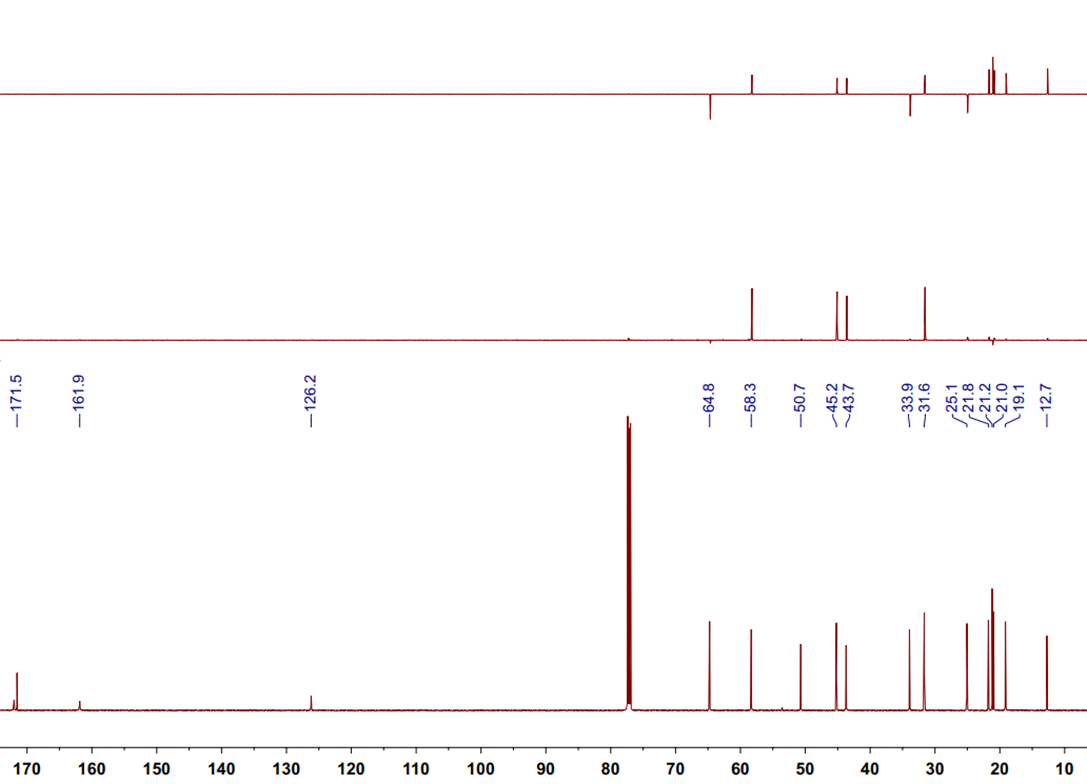


**Fig. S70** ^13^C NMR spectrum of **14.**


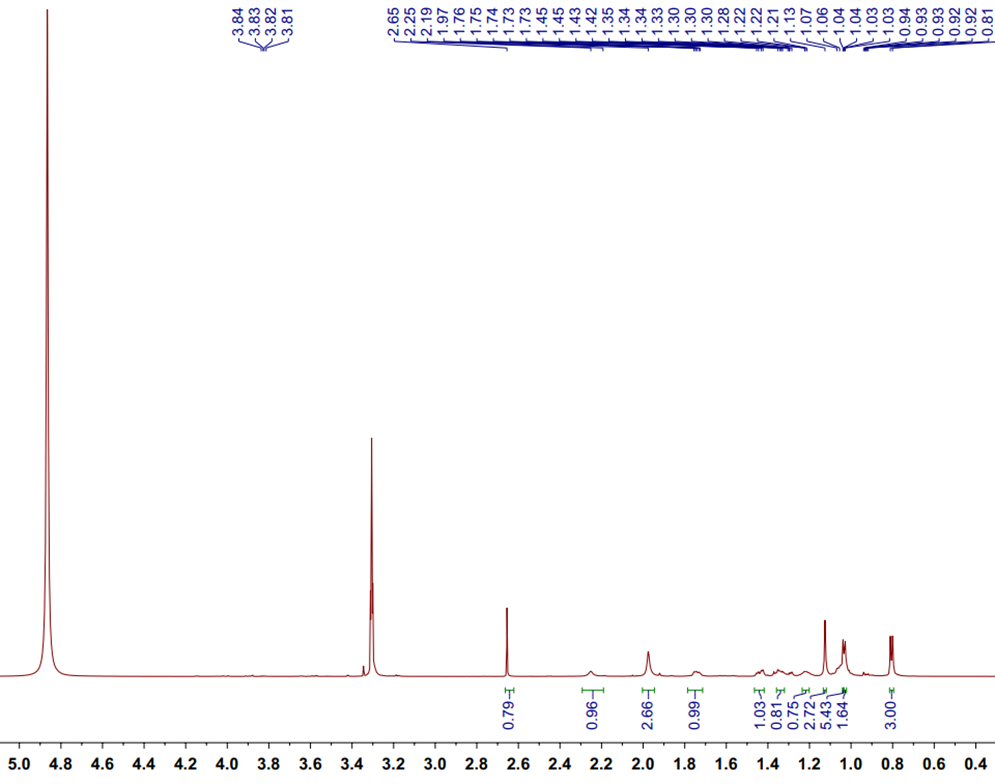


**Fig. S71** ^1^H NMR spectrum of **15.**


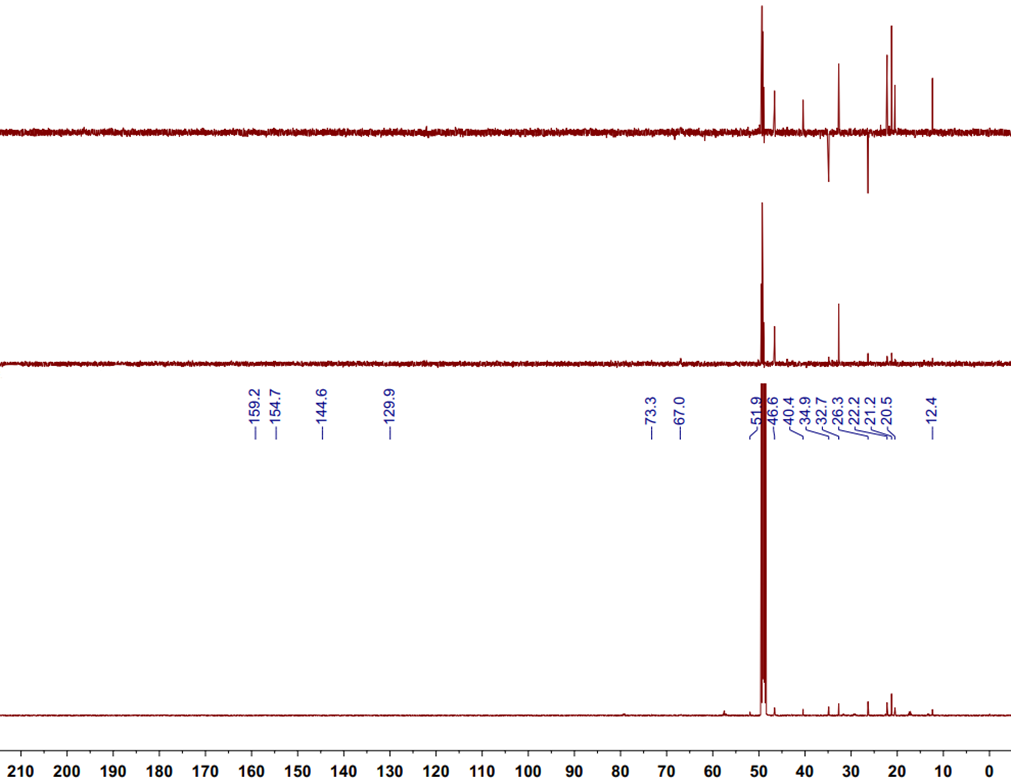


**Fig. S72** ^13^C NMR spectrum of **15.**


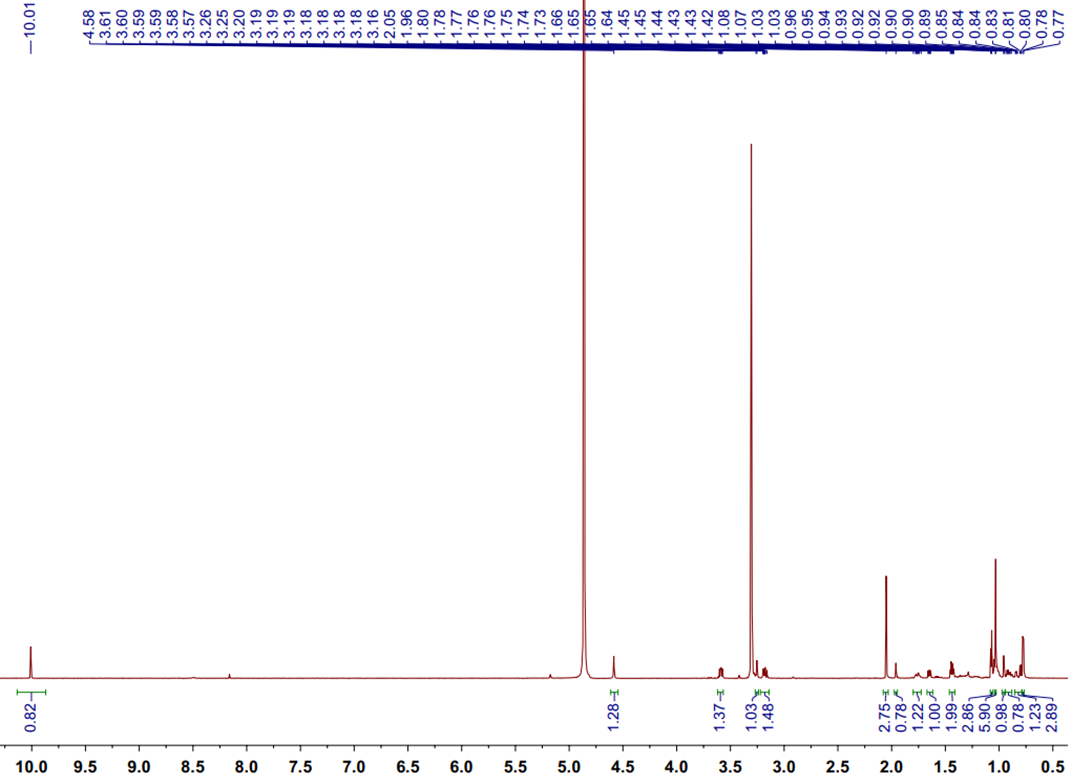


**Fig. S73** ^1^H NMR spectrum of **16.**


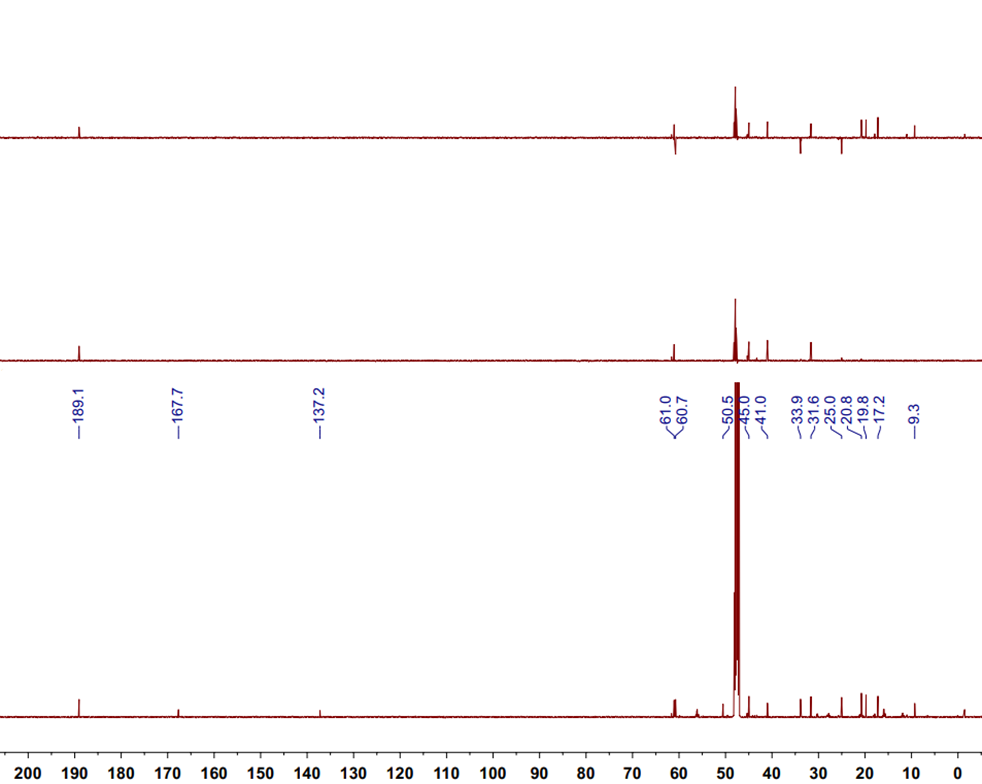


**Fig. S74** ^13^C NMR spectrum of **16.**


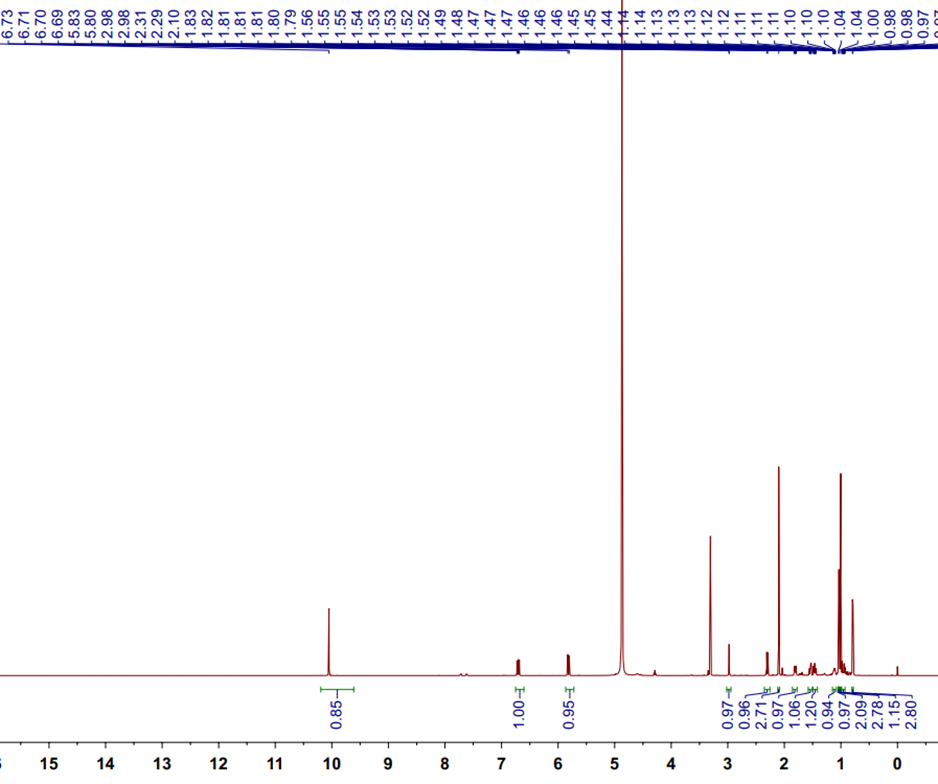


**Fig. S75** ^1^H NMR spectrum of **17.**


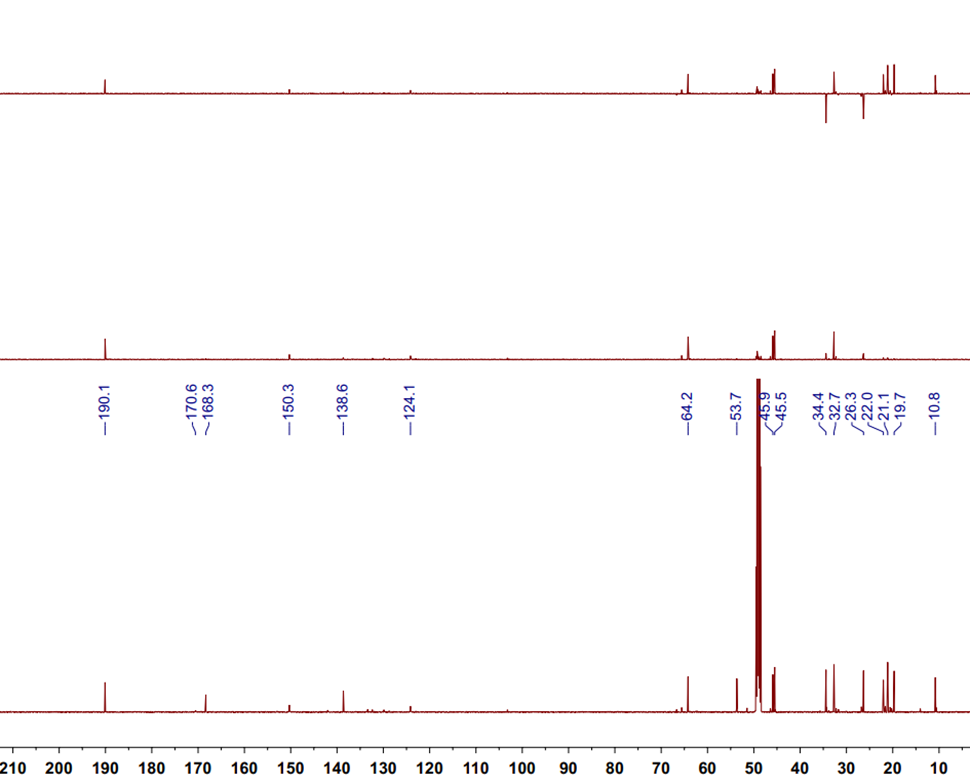


**Fig. S76** ^13^C NMR spectrum of **17.**

**Quantum chemical calculation**

DP4+(all date) 100.00%

**Fig. S77**. Correlations between calculated and experimental ^13^C NMR chemical shifts of **1A** and **1B.**

**
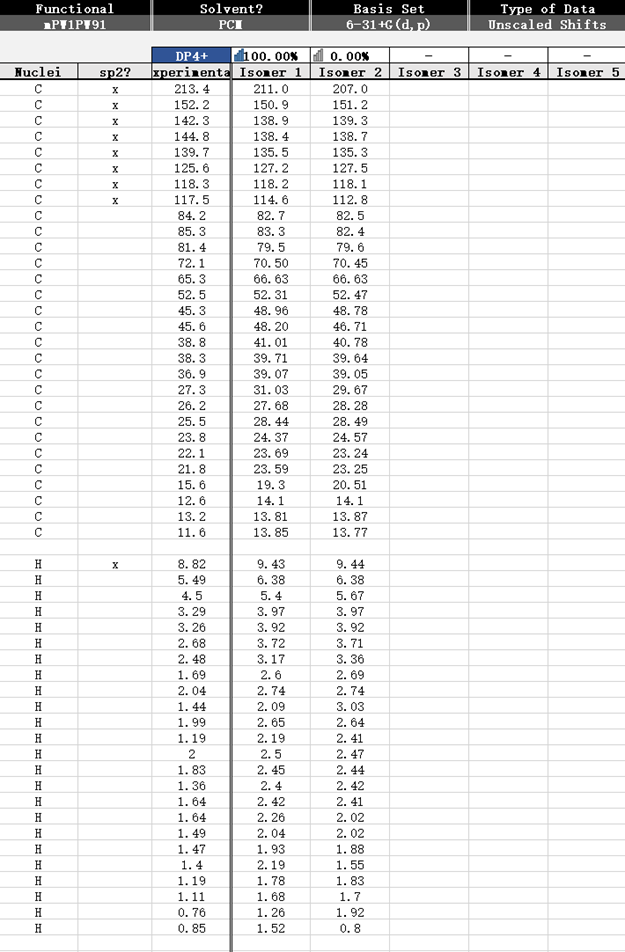
**

**
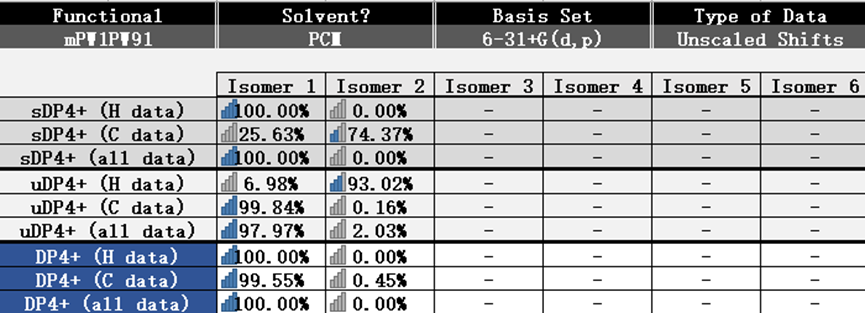
**

**Fig. S78**. DP4+ analysis results of **1.**

**Table S1.** Energy analysis for conformers of **1Aa**~**1Ae** at M062X/def2svp level in the gas phase.

| Temperature: 298.15 | | Q（relat）: 2.655928 | |
| --- | --- | --- | --- |
| conformer | ΔG (kcal/mol)^a^ | Qi(Relat) | Population^b^ |
| **1Aa** | 0 | 1 | 37.65% |
| **1Ab** | 0.116715 | 0.821091 | 30.92% |
| **1Ac** | 0.518315 | 0.416699 | 15.69% |
| **1Ad** | 0.939368 | 0.204638 | 7.70% |
| **1Ae** | 0.914268 | 0.2135 | 8.04% |

^a^The relative Gibbs free energy; ^b^The Boltzmann distribution of each conformer.

**Table S2**. Cartesian coordinates for the low-energy optimized conformers of **1A** at M062X/def2svp level.

| Conformer **1Aa** | | | | | | | | | | | |
| --- | --- | --- | --- | --- | --- | --- | --- | --- | --- | --- | --- |
| C | 0.791919 | 4.951973 | 0.426007 | C | -1.78441 | -5.12228 | 0.384376 | H | -1.84544 | 3.320656 | -1.14821 |
| C | 2.088292 | 5.015878 | -0.39485 | S | -3.97573 | -3.11724 | 2.044351 | H | -0.95604 | 2.909737 | -2.61129 |
| O | 1.921794 | 4.268584 | -1.62085 | C | -4.2799 | -1.46076 | 2.295271 | H | -0.66447 | 4.430273 | -1.82837 |
| C | 1.64416 | 2.892494 | -1.36293 | N | -3.47175 | -0.62479 | 1.689325 | H | 4.643511 | 6.036086 | -0.9648 |
| C | 0.228907 | 2.724008 | -0.75122 | C | -1.64985 | -5.67115 | 1.806373 | H | 3.7468 | 5.872621 | -2.49164 |
| C | 0.265849 | 3.518544 | 0.587395 | C | -0.29826 | -5.47694 | 2.497915 | H | 4.108291 | 7.468488 | -1.87049 |
| C | 1.841459 | 2.092465 | -2.64581 | C | 0.346959 | -6.83396 | 2.81203 | H | 3.331764 | 6.998443 | 1.15888 |
| C | 1.639358 | 0.606067 | -2.37538 | C | 0.739876 | -7.60256 | 1.557207 | H | 1.657995 | 7.550559 | 0.902476 |
| C | 0.260405 | 0.274404 | -1.76324 | C | -3.01584 | -5.67415 | -0.33691 | H | 2.971357 | 8.386327 | 0.110778 |
| C | -0.01096 | 1.181716 | -0.52294 | O | -2.56924 | -6.27004 | 2.369606 | H | 1.318915 | 6.303028 | -2.3095 |
| O | 0.377164 | -1.10248 | -1.31784 | C | -0.50003 | -4.66388 | 3.774652 | H | -2.26547 | 0.949038 | 1.814704 |
| C | -0.63712 | -1.56045 | -0.51163 | H | 0.014241 | 5.558372 | -0.05208 | H | 0.757218 | -3.07558 | -1.5583 |
| C | -1.50472 | -0.721 | 0.187917 | H | 0.951289 | 5.38167 | 1.421487 | H | -0.9241 | -5.54342 | -0.15061 |
| C | -1.36107 | 0.779961 | 0.100686 | H | 2.904723 | 4.560479 | 0.181954 | H | -5.09515 | -1.12423 | 2.923033 |
| C | -0.74282 | -2.95085 | -0.4156 | H | 2.396627 | 2.526516 | -0.64805 | H | 0.3673 | -4.91208 | 1.835231 |
| C | -1.73419 | -3.60052 | 0.341342 | H | 0.90915 | 2.9954 | 1.306949 | H | -0.33256 | -7.45361 | 3.409817 |
| C | -2.62931 | -2.7335 | 1.01911 | H | -0.73049 | 3.580762 | 1.03761 | H | 1.249162 | -6.67678 | 3.415711 |
| C | -2.52823 | -1.33634 | 0.951438 | H | 1.177561 | 2.451654 | -3.43929 | H | 1.419865 | -7.01388 | 0.933251 |
| C | -0.78846 | 0.277163 | -2.89672 | H | 2.857459 | 2.259632 | -3.02517 | H | -0.1372 | -7.86652 | 0.958515 |
| C | -0.8645 | 3.372133 | -1.63195 | H | 1.792182 | 0.0359 | -3.30091 | H | 1.249675 | -8.53208 | 1.830005 |
| C | 2.505745 | 6.462508 | -0.78904 | H | 2.428946 | 0.256414 | -1.69539 | H | -2.98088 | -6.7691 | -0.36925 |
| C | 3.829091 | 6.453039 | -1.56603 | H | 0.747991 | 0.892482 | 0.225417 | H | -3.9524 | -5.39982 | 0.156859 |
| C | 2.627627 | 7.39184 | 0.418824 | H | -2.19634 | 1.170387 | -0.48972 | H | -3.06195 | -5.30849 | -1.36866 |
| O | 1.509762 | 7.012442 | -1.66414 | H | -0.71741 | 1.147057 | -3.55037 | H | -1.15855 | -5.18171 | 4.480689 |
| O | -1.4621 | 1.336714 | 1.408884 | H | -1.81439 | 0.217453 | -2.52336 | H | -0.95339 | -3.69247 | 3.552971 |
| O | 0.170209 | -3.71357 | -1.10467 | H | -0.65268 | -0.6046 | -3.5363 | H | 0.456796 | -4.48057 | 4.274477 |

| Conformer **1Ab** | | | | | | | | | | | |
| --- | --- | --- | --- | --- | --- | --- | --- | --- | --- | --- | --- |
| C | 4.79388 | 1.498758 | -0.00742 | C | -5.14341 | -1.34227 | -1.13779 | H | 3.443662 | -1.48426 | -1.14865 |
| C | 5.000382 | 1.557271 | 1.509432 | S | -3.44676 | -0.96098 | -4.06247 | H | 3.275424 | -2.23665 | 0.434317 |
| O | 4.46203 | 0.374888 | 2.143544 | C | -1.85515 | -0.70804 | -4.61211 | H | 4.648841 | -1.22879 | 0.105087 |
| C | 3.061552 | 0.242206 | 1.897802 | N | -0.92762 | -0.63451 | -3.68827 | H | 7.225697 | 3.003394 | 0.369017 |
| C | 2.789839 | -0.07782 | 0.403778 | C | -5.9172 | -0.181 | -1.76557 | H | 6.515927 | 3.860164 | 1.75602 |
| C | 3.348586 | 1.144148 | -0.38562 | C | -5.83438 | 1.171031 | -1.05293 | H | 8.107787 | 3.138572 | 1.902106 |
| C | 2.484332 | -0.79991 | 2.849291 | C | -7.22245 | 1.610364 | -0.56641 | H | 7.477179 | 0.410372 | 0.487273 |
| C | 0.973145 | -0.89194 | 2.674367 | C | -7.77439 | 0.70741 | 0.528916 | H | 6.929658 | -0.44176 | 1.947698 |
| C | 0.541182 | -1.21684 | 1.227549 | C | -5.57488 | -2.69852 | -1.69963 | H | 8.348521 | 0.582022 | 2.019792 |
| C | 1.230224 | -0.23809 | 0.225918 | O | -6.60288 | -0.32079 | -2.7812 | H | 5.902504 | 1.023792 | 3.683288 |
| O | -0.89004 | -0.97418 | 1.207534 | C | -5.2415 | 2.205595 | -2.00687 | H | 0.605665 | 0.354349 | -2.91461 |
| C | -1.47804 | -0.95105 | -0.03427 | H | 5.45662 | 0.751171 | -0.45459 | H | -2.79574 | -1.21564 | 1.844639 |
| C | -0.76815 | -0.7596 | -1.22016 | H | 5.057753 | 2.458809 | -0.46501 | H | -5.46758 | -1.3481 | -0.08966 |
| C | 0.72763 | -0.55075 | -1.19657 | H | 4.447202 | 2.415292 | 1.91675 | H | -1.6297 | -0.61203 | -5.66658 |
| C | -2.86582 | -1.11727 | -0.04149 | H | 2.583994 | 1.199192 | 2.157226 | H | -5.16671 | 1.081918 | -0.18836 |
| C | -3.63494 | -1.14682 | -1.21851 | H | 2.717574 | 2.022522 | -0.19726 | H | -7.93359 | 1.631375 | -1.40122 |
| C | -2.8945 | -0.98493 | -2.4176 | H | 3.326155 | 0.96121 | -1.46497 | H | -7.16322 | 2.632289 | -0.17238 |
| C | -1.50471 | -0.79738 | -2.43062 | H | 2.966863 | -1.77261 | 2.706466 | H | -7.08955 | 0.661266 | 1.381625 |
| C | 0.728227 | -2.73187 | 0.993222 | H | 2.715291 | -0.51475 | 3.883438 | H | -7.93963 | -0.31051 | 0.16298 |
| C | 3.572942 | -1.32284 | -0.07338 | H | 0.563178 | -1.63538 | 3.370258 | H | -8.73442 | 1.093537 | 0.886068 |
| C | 6.482125 | 1.696145 | 1.959116 | H | 0.519305 | 0.061094 | 2.980703 | H | -5.38891 | -2.7889 | -2.77363 |
| C | 7.116248 | 2.993231 | 1.457669 | H | 0.822138 | 0.76068 | 0.461621 | H | -5.04548 | -3.51635 | -1.19841 |
| C | 7.351995 | 0.496022 | 1.570484 | H | 1.205468 | -1.45384 | -1.59009 | H | -6.64962 | -2.85046 | -1.54791 |
| O | 6.484358 | 1.7564 | 3.398126 | H | 1.696399 | -3.10568 | 1.327688 | H | -4.2484 | 1.898406 | -2.34995 |
| O | 1.065583 | 0.526466 | -2.0665 | H | 0.59905 | -3.01689 | -0.05441 | H | -5.13923 | 3.177188 | -1.51241 |
| O | -3.50151 | -1.26428 | 1.168641 | H | -0.03214 | -3.29458 | 1.550199 | H | -5.86986 | 2.34031 | -2.89417 |

| Conformer **1Ac** | | | | | | | | | | | |
| --- | --- | --- | --- | --- | --- | --- | --- | --- | --- | --- | --- |
| C | 1.731353 | 4.69597 | -0.67834 | C | -2.87914 | -4.47272 | 0.997751 | H | -0.53562 | 2.935207 | -2.64173 |
| C | 3.204975 | 4.341085 | -0.9253 | S | -4.98367 | -1.80595 | 1.089632 | H | 0.669441 | 1.966904 | -3.48477 |
| O | 3.288099 | 3.252452 | -1.87249 | C | -4.97603 | -0.1373 | 0.748315 | H | 1.02024 | 3.61925 | -3.08887 |
| C | 2.640231 | 2.079209 | -1.38159 | N | -3.84734 | 0.357589 | 0.300502 | H | 5.965192 | 4.827063 | -0.72559 |
| C | 1.102929 | 2.279813 | -1.33707 | C | -3.44867 | -4.52785 | 2.420304 | H | 5.608057 | 4.266 | -2.37474 |
| C | 0.874455 | 3.464277 | -0.35249 | C | -2.53362 | -4.07468 | 3.56756 | H | 6.092955 | 5.927569 | -2.11522 |
| C | 3.064027 | 0.882303 | -2.22572 | C | -1.43861 | -5.11041 | 3.863352 | H | 4.278981 | 6.577366 | 0.392413 |
| C | 2.456603 | -0.3984 | -1.6652 | C | -1.95089 | -6.46844 | 4.325163 | H | 2.951456 | 7.19358 | -0.62249 |
| C | 0.915891 | -0.35458 | -1.56269 | C | -3.82533 | -5.10323 | -0.02787 | H | 4.603963 | 7.569705 | -1.04435 |
| C | 0.462115 | 0.936906 | -0.81339 | O | -4.58352 | -4.95778 | 2.644566 | H | 3.417588 | 4.996567 | -3.25783 |
| O | 0.567901 | -1.50963 | -0.7549 | C | -3.35164 | -3.71801 | 4.809511 | H | -2.42856 | 1.744174 | 0.449768 |
| C | -0.7303 | -1.55981 | -0.30703 | H | 1.311081 | 5.190989 | -1.56117 | H | 0.551993 | -3.48286 | -0.29537 |
| C | -1.5685 | -0.44562 | -0.24593 | H | 1.646353 | 5.411306 | 0.147549 | H | -2.00042 | -5.12674 | 1.026457 |
| C | -1.07164 | 0.915757 | -0.67119 | H | 3.662174 | 4.016081 | 0.019151 | H | -5.85649 | 0.474643 | 0.897896 |
| C | -1.17035 | -2.81912 | 0.110158 | H | 3.013296 | 1.890842 | -0.36348 | H | -2.04185 | -3.14806 | 3.248857 |
| C | -2.47387 | -3.06619 | 0.575476 | H | 1.110636 | 3.142143 | 0.670112 | H | -0.76773 | -4.7105 | 4.633833 |
| C | -3.3245 | -1.93141 | 0.597834 | H | -0.17373 | 3.78141 | -0.35231 | H | -0.82116 | -5.26034 | 2.970231 |
| C | -2.89696 | -0.65623 | 0.20153 | H | 2.796512 | 1.028477 | -3.2776 | H | -2.60828 | -6.92419 | 3.578804 |
| C | 0.327314 | -0.61295 | -2.96687 | H | 4.15833 | 0.801449 | -2.21725 | H | -2.49645 | -6.39291 | 5.270038 |
| C | 0.534923 | 2.71504 | -2.70792 | H | 2.773583 | -1.25651 | -2.27201 | H | -1.10793 | -7.14878 | 4.485227 |
| C | 4.041716 | 5.519862 | -1.50214 | H | 2.878836 | -0.58707 | -0.6681 | H | -4.04271 | -6.14377 | 0.238639 |
| C | 5.509179 | 5.107812 | -1.68049 | H | 0.847129 | 0.830867 | 0.216276 | H | -4.78442 | -4.57994 | -0.08914 |
| C | 3.96451 | 6.77787 | -0.63674 | H | -1.55593 | 1.177136 | -1.61768 | H | -3.37824 | -5.09989 | -1.02819 |
| O | 3.538964 | 5.854321 | -2.80428 | H | 0.802242 | -0.02239 | -3.75089 | H | -3.9363 | -4.56724 | 5.177417 |
| O | -1.47051 | 1.882562 | 0.297196 | H | -0.75012 | -0.43272 | -3.01325 | H | -4.05969 | -2.91306 | 4.582516 |
| O | -0.27935 | -3.86414 | 0.052103 | H | 0.465082 | -1.66617 | -3.24393 | H | -2.70025 | -3.37396 | 5.619487 |

| Conformer **1Ad** | | | | | | | | | | | |
| --- | --- | --- | --- | --- | --- | --- | --- | --- | --- | --- | --- |
| C | 3.865294 | 1.449192 | 2.788312 | C | -3.49211 | -2.54064 | -3.38225 | H | 1.950144 | -1.42998 | 3.158892 |
| C | 3.228299 | 2.722005 | 3.365086 | S | -0.64142 | -4.38922 | -3.21965 | H | 0.593237 | -0.64262 | 3.958495 |
| O | 1.898869 | 2.42204 | 3.846597 | C | 0.815207 | -4.44816 | -2.33943 | H | 2.211668 | -0.11862 | 4.299277 |
| C | 1.052736 | 1.95666 | 2.795549 | N | 0.96512 | -3.54017 | -1.40546 | H | 3.320884 | 5.38285 | 4.262951 |
| C | 1.491617 | 0.54641 | 2.32216 | C | -3.00783 | -2.53115 | -4.83508 | H | 2.334069 | 4.436738 | 5.400106 |
| C | 2.944963 | 0.727538 | 1.793465 | C | -2.49525 | -1.19749 | -5.38725 | H | 3.897769 | 5.036922 | 5.907959 |
| C | -0.39843 | 2.007829 | 3.260582 | C | -3.6558 | -0.47581 | -6.08536 | H | 5.570953 | 4.271828 | 3.335787 |
| C | -1.32976 | 1.601735 | 2.124328 | C | -3.3095 | 0.940571 | -6.51966 | H | 6.035485 | 2.688636 | 4.006447 |
| C | -1.01913 | 0.20408 | 1.54442 | C | -4.25298 | -3.82167 | -3.0335 | H | 6.001322 | 4.089187 | 5.04928 |
| C | 0.495626 | 0.093914 | 1.185702 | O | -3.07707 | -3.53092 | -5.55434 | H | 3.080049 | 2.100312 | 5.71169 |
| O | -1.79536 | 0.133131 | 0.319585 | C | -1.30403 | -1.44253 | -6.31175 | H | 2.092519 | -2.11762 | -0.59242 |
| C | -1.50203 | -0.91692 | -0.51693 | H | 4.117786 | 0.755285 | 3.598133 | H | -3.49706 | 0.219953 | -0.77477 |
| C | -0.30964 | -1.6394 | -0.45861 | H | 4.807212 | 1.69138 | 2.283181 | H | -4.23416 | -1.73407 | -3.3391 |
| C | 0.756694 | -1.28504 | 0.55055 | H | 3.148714 | 3.478138 | 2.572255 | H | 1.571289 | -5.19483 | -2.54642 |
| C | -2.48354 | -1.21782 | -1.46539 | H | 1.137207 | 2.665456 | 1.957888 | H | -2.14256 | -0.58904 | -4.54701 |
| C | -2.3688 | -2.2728 | -2.38816 | H | 2.926598 | 1.305967 | 0.860536 | H | -4.5172 | -0.42744 | -5.40764 |
| C | -1.16773 | -3.02101 | -2.29161 | H | 3.403797 | -0.23898 | 1.559842 | H | -3.98737 | -1.05115 | -6.95881 |
| C | -0.16257 | -2.72334 | -1.36016 | H | -0.54928 | 1.383854 | 4.147872 | H | -4.19543 | 1.433517 | -6.93281 |
| C | -1.60982 | -0.85957 | 2.495546 | H | -0.64022 | 3.028222 | 3.583762 | H | -2.53704 | 0.945427 | -7.29411 |
| C | 1.558164 | -0.46324 | 3.491551 | H | -2.37268 | 1.650068 | 2.463476 | H | -2.95562 | 1.535759 | -5.67197 |
| C | 4.023616 | 3.332638 | 4.55546 | H | -1.25974 | 2.34878 | 1.321138 | H | -3.62933 | -4.71646 | -3.12046 |
| C | 3.355488 | 4.622624 | 5.05001 | H | 0.659012 | 0.820778 | 0.370433 | H | -4.63878 | -3.78168 | -2.0088 |
| C | 5.485302 | 3.614123 | 4.206615 | H | 0.780192 | -2.06933 | 1.314124 | H | -5.10559 | -3.95742 | -3.70847 |
| O | 4.0071 | 2.405388 | 5.650967 | H | -1.37485 | -0.68027 | 3.545142 | H | -1.60695 | -1.96412 | -7.22622 |
| O | 2.027264 | -1.27563 | -0.0953 | H | -1.28927 | -1.87463 | 2.245719 | H | -0.54703 | -2.05944 | -5.8154 |
| O | -3.61788 | -0.44151 | -1.48553 | H | -2.70481 | -0.8658 | 2.418432 | H | -0.82565 | -0.50141 | -6.59948 |

| Conformer **1Ae** | | | | | | | | | | | |
| --- | --- | --- | --- | --- | --- | --- | --- | --- | --- | --- | --- |
| C | 3.545122 | 2.056936 | -2.935 | C | -4.44694 | -2.08665 | 2.266815 | H | 2.28063 | -1.1713 | -2.99045 |
| C | 4.611574 | 2.297437 | -1.86179 | S | -4.94522 | -1.80526 | -1.08314 | H | 3.224103 | -1.7703 | -1.62985 |
| O | 4.762826 | 1.130214 | -1.02237 | C | -4.07335 | -1.42025 | -2.49469 | H | 3.937353 | -0.6413 | -2.73759 |
| C | 3.541454 | 0.802044 | -0.35914 | N | -2.80383 | -1.13492 | -2.33225 | H | 5.431189 | 3.879011 | -4.13573 |
| C | 2.479779 | 0.297802 | -1.37252 | C | -5.67114 | -1.16821 | 2.173035 | H | 5.575007 | 4.781784 | -2.61063 |
| C | 2.24447 | 1.490474 | -2.34756 | C | -5.45781 | 0.3267 | 2.453147 | H | 7.02306 | 4.279886 | -3.46345 |
| C | 3.831158 | -0.19855 | 0.754136 | C | -5.25241 | 0.599895 | 3.950631 | H | 6.11521 | 1.362776 | -4.17697 |
| C | 2.559404 | -0.49277 | 1.540796 | C | -6.43621 | 0.232748 | 4.83594 | H | 6.704554 | 0.60641 | -2.68053 |
| C | 1.40171 | -1.01659 | 0.662847 | C | -4.8228 | -3.56511 | 2.134176 | H | 7.692522 | 1.798305 | -3.49894 |
| C | 1.179347 | -0.07007 | -0.55812 | O | -6.79434 | -1.61395 | 1.922562 | H | 6.712897 | 2.107306 | -0.69007 |
| O | 0.23215 | -0.95462 | 1.520697 | C | -6.59249 | 1.160802 | 1.856887 | H | -1.29546 | 0.109865 | -2.66731 |
| C | -0.97985 | -1.1324 | 0.897995 | H | 3.913474 | 1.353907 | -3.68875 | H | -0.83531 | -1.36446 | 3.193442 |
| C | -1.17065 | -0.97959 | -0.47609 | H | 3.321419 | 2.989381 | -3.4653 | H | -4.08949 | -1.96648 | 3.295639 |
| C | -0.0207 | -0.58812 | -1.37378 | H | 4.287101 | 3.118768 | -1.20739 | H | -4.54805 | -1.41089 | -3.46763 |
| C | -2.04198 | -1.46758 | 1.742449 | H | 3.171044 | 1.714979 | 0.131301 | H | -4.54513 | 0.632152 | 1.928024 |
| C | -3.34755 | -1.71134 | 1.281197 | H | 1.723906 | 2.299623 | -1.81889 | H | -5.02854 | 1.664597 | 4.091827 |
| C | -3.51765 | -1.58716 | -0.12144 | H | 1.605588 | 1.195143 | -3.18635 | H | -4.37198 | 0.053719 | 4.308272 |
| C | -2.46972 | -1.23364 | -0.98329 | H | 4.27823 | -1.11431 | 0.353003 | H | -6.68658 | -0.82887 | 4.751072 |
| C | 1.649707 | -2.51335 | 0.374065 | H | 4.589365 | 0.218524 | 1.428882 | H | -7.32371 | 0.820925 | 4.585859 |
| C | 3.003964 | -0.88455 | -2.22003 | H | 2.77781 | -1.20457 | 2.347449 | H | -6.19264 | 0.433941 | 5.884392 |
| C | 6.018428 | 2.662854 | -2.41342 | H | 2.236463 | 0.424063 | 2.053836 | H | -5.27337 | -3.79671 | 1.164231 |
| C | 6.003527 | 3.968701 | -3.20749 | H | 0.842143 | 0.890167 | -0.12932 | H | -3.94249 | -4.20613 | 2.25537 |
| C | 6.656648 | 1.544564 | -3.24415 | H | 0.260515 | -1.46192 | -1.97056 | H | -5.5511 | -3.84692 | 2.903059 |
| O | 6.877968 | 2.872156 | -1.27687 | H | 2.667914 | -2.72891 | 0.048886 | H | -7.568 | 0.889099 | 2.27274 |
| O | -0.45409 | 0.425918 | -2.27671 | H | 0.962705 | -2.91934 | -0.37337 | H | -6.65003 | 1.008464 | 0.773245 |
| O | -1.78642 | -1.5689 | 3.089174 | H | 1.481769 | -3.10407 | 1.283937 | H | -6.42815 | 2.22803 | 2.037871 |

**Fig. S79.** Correlations between calculated and experimental ^13^C NMR chemical shifts of **2A** and **2B.**

**
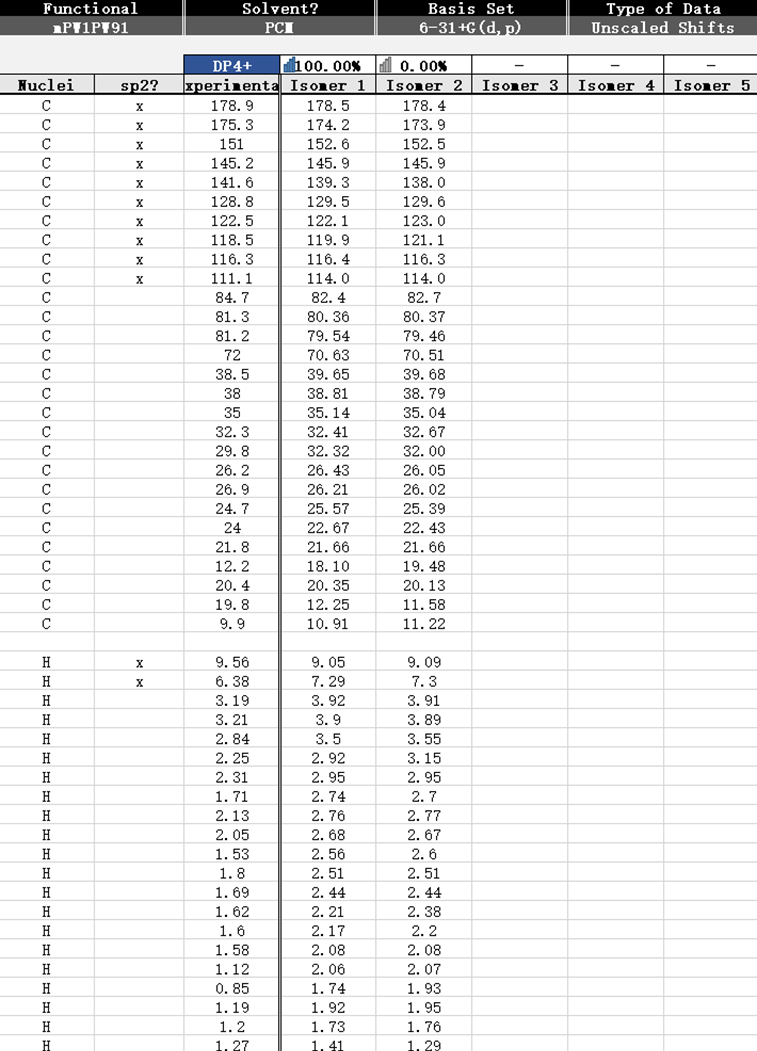
**

**
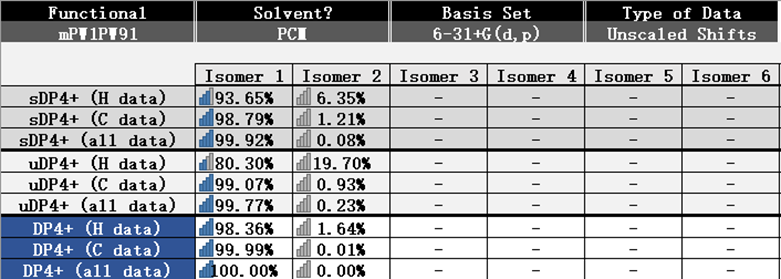
**

**Fig. S80**. DP4+ analysis results of **2.**

**Table S3.** Energy analysis for conformers of **2Aa**~**2Ae** at M062X/def2svp level in the gas phase.

| Temperature: 298.15 | | Q（relat）: 1.676510 | |
| --- | --- | --- | --- |
| conformer | ΔG (kcal/mol)^a^ | Qi(Relat) | Population^b^ |
| **2Aa** | 0.515742 | 0.418514 | 24.96% |
| **2Ab** | 0.830622 | 0.245895 | 14.67% |
| **2Ac** | 2.914549 | 0.007281 | 0.43% |
| **2Ad** | 3.158835 | 0.00482 | 0.29% |
| **2Ae** | 0 | 1 | 59.65% |

^a^The relative Gibbs free energy; ^b^The Boltzmann distribution of each conformer.

**Table S4**. Cartesian coordinates for the low-energy optimized conformers of **2A** at M062X/def2svp level.

| Conformer **2Aa** | | | | | | | | | | | |
| --- | --- | --- | --- | --- | --- | --- | --- | --- | --- | --- | --- |
| C | -2.73364 | -3.23748 | -2.42722 | C | -3.0097 | -2.14093 | 0.443065 | H | -2.04554 | -7.17528 | -0.9725 |
| C | -2.0914 | -4.5814 | -2.04832 | C | 3.240479 | 6.488555 | 0.387773 | H | -4.00149 | -5.00427 | -4.16026 |
| O | -1.86722 | -4.62562 | -0.62141 | C | 2.743862 | 5.073249 | 0.560766 | H | -2.4638 | -5.81745 | -4.54275 |
| C | -0.98063 | -3.58891 | -0.19377 | C | 3.14675 | 4.063441 | 1.431283 | H | -3.92004 | -6.74864 | -4.13435 |
| C | -1.66176 | -2.20131 | -0.31655 | C | 1.466967 | 3.278424 | 0.140491 | H | -0.18984 | -0.99749 | 3.699304 |
| C | -1.97921 | -2.03892 | -1.83492 | C | 4.217974 | 4.120909 | 2.466373 | H | -1.29251 | -0.06836 | 2.700852 |
| C | -0.507 | -3.8911 | 1.224621 | N | 1.717483 | 4.572945 | -0.21076 | H | -1.6026 | -1.78564 | 3.00585 |
| C | 0.525241 | -2.85495 | 1.650454 | C | 3.772941 | 6.703831 | -1.03316 | H | -3.72969 | -2.8797 | 0.078337 |
| C | 0.028415 | -1.39631 | 1.564837 | C | 2.156183 | 7.530883 | 0.719605 | H | -2.90724 | -2.33657 | 1.510201 |
| C | -0.71537 | -1.11565 | 0.244378 | C | 1.726593 | 7.502191 | 2.181598 | H | -3.47329 | -1.15307 | 0.335201 |
| O | 1.239815 | -0.60649 | 1.703591 | O | 3.068988 | 1.241733 | 2.638136 | H | 4.084207 | 6.654165 | 1.068393 |
| C | 1.266412 | 0.667538 | 1.155769 | H | -2.76672 | -3.12736 | -3.51709 | H | 4.628938 | 5.127248 | 2.580303 |
| C | 0.425474 | 1.033025 | 0.165645 | H | -3.77285 | -3.2052 | -2.08161 | H | 3.823394 | 3.815846 | 3.441023 |
| C | -0.49794 | 0.059603 | -0.37661 | H | -1.12118 | -4.67439 | -2.55492 | H | 5.039425 | 3.449096 | 2.197558 |
| C | 0.479065 | 2.412997 | -0.43246 | H | -2.57229 | -1.13393 | -2.01749 | H | 1.217955 | 5.077716 | -0.93392 |
| C | -2.96536 | -5.81489 | -2.41844 | H | -1.04172 | -1.92098 | -2.39572 | H | 4.215376 | 7.700906 | -1.13278 |
| O | -4.17961 | -5.76921 | -1.65488 | H | -0.05715 | -4.89144 | 1.253658 | H | 4.547581 | 5.968164 | -1.27724 |
| C | -2.24896 | -7.12035 | -2.04767 | H | -1.34711 | -3.93231 | 1.925956 | H | 2.979145 | 6.614424 | -1.78317 |
| C | -3.34832 | -5.84337 | -3.89839 | H | 1.418158 | -2.96858 | 1.018805 | H | 2.543271 | 8.533734 | 0.499428 |
| C | -0.82042 | -1.05239 | 2.803162 | H | 0.87129 | -3.07692 | 2.668468 | H | 1.272994 | 7.389787 | 0.084446 |
| C | 2.312573 | 1.593868 | 1.74049 | H | -0.99718 | 0.332175 | -1.30228 | H | 1.014189 | 8.309704 | 2.378797 |
| C | 2.330519 | 2.922616 | 1.150708 | H | -3.89833 | -5.58257 | -0.73729 | H | 1.237776 | 6.557852 | 2.439377 |
| O | -0.27031 | 2.755775 | -1.34063 | H | -2.87721 | -7.98802 | -2.28053 | H | 2.585868 | 7.639775 | 2.845653 |
| H | -0.08881 | -3.6203 | -0.83832 | H | -1.30188 | -7.22443 | -2.58694 |  |  |  |  |

| Conformer **2Ab** | | | | | | | | | | | |
| --- | --- | --- | --- | --- | --- | --- | --- | --- | --- | --- | --- |
| C | -1.56108 | -3.07628 | -3.45017 | C | -2.84472 | -2.2148 | -0.78436 | H | -0.25932 | -5.58473 | -5.35457 |
| C | -1.06242 | -4.44246 | -2.96518 | C | 2.785554 | 6.40168 | 1.975549 | H | -3.44992 | -5.83642 | -2.44395 |
| O | -1.33026 | -4.61189 | -1.55496 | C | 2.298908 | 4.976941 | 1.861752 | H | -3.74446 | -4.97412 | -3.96982 |
| C | -0.681 | -3.60657 | -0.77164 | C | 2.399932 | 3.903602 | 2.743542 | H | -3.55425 | -6.73501 | -3.94327 |
| C | -1.3152 | -2.2137 | -1.02584 | C | 1.299126 | 3.218778 | 0.893295 | H | -1.37446 | -1.32215 | 3.32759 |
| C | -1.08632 | -1.93163 | -2.54388 | C | 3.040121 | 3.882005 | 4.089634 | H | -2.08641 | -0.3222 | 2.074856 |
| C | -0.72361 | -4.01617 | 0.697413 | N | 1.621108 | 4.536527 | 0.745667 | H | -2.43198 | -2.05886 | 2.129045 |
| C | 0.06493 | -3.01431 | 1.530947 | C | 3.775887 | 6.725109 | 0.851478 | H | -3.37002 | -2.92772 | -1.427 |
| C | -0.41292 | -1.55452 | 1.382269 | C | 1.625528 | 7.414898 | 1.978549 | H | -3.11553 | -2.4882 | 0.235145 |
| C | -0.65623 | -1.1733 | -0.09141 | C | 0.711936 | 7.274342 | 3.190321 | H | -3.27041 | -1.22348 | -0.98054 |
| O | 0.649825 | -0.77551 | 1.994036 | O | 1.980256 | 0.998641 | 3.642875 | H | 3.332461 | 6.515518 | 2.919195 |
| C | 0.829883 | 0.537102 | 1.583703 | H | -1.217 | -2.88749 | -4.47327 | H | 3.363025 | 4.875896 | 4.409754 |
| C | 0.378267 | 0.976821 | 0.390537 | H | -2.65468 | -3.05719 | -3.48432 | H | 2.336479 | 3.508205 | 4.840543 |
| C | -0.26872 | 0.046881 | -0.50995 | H | 0.028576 | -4.49324 | -3.08868 | H | 3.918441 | 3.228666 | 4.079817 |
| C | 0.597581 | 2.399157 | -0.04966 | H | -1.5989 | -1.01228 | -2.8539 | H | 1.392398 | 5.094919 | -0.06866 |
| C | -1.67812 | -5.66089 | -3.70934 | H | -0.01501 | -1.77513 | -2.73107 | H | 4.197548 | 7.727228 | 0.984849 |
| O | -1.08025 | -6.8453 | -3.15033 | H | -0.2838 | -5.01499 | 0.810574 | H | 4.60689 | 6.010812 | 0.843501 |
| C | -1.3421 | -5.65395 | -5.20039 | H | -1.75421 | -4.11269 | 1.055091 | H | 3.297894 | 6.692506 | -0.13395 |
| C | -3.19102 | -5.79659 | -3.50766 | H | 1.124964 | -3.07682 | 1.245285 | H | 2.037527 | 8.431893 | 1.980513 |
| C | -1.65056 | -1.30972 | 2.265825 | H | 0.040482 | -3.31397 | 2.586875 | H | 1.025065 | 7.322147 | 1.065206 |
| C | 1.57801 | 1.417211 | 2.563581 | H | -0.41903 | 0.38993 | -1.52972 | H | -0.04632 | 8.063937 | 3.181596 |
| C | 1.764714 | 2.787154 | 2.11388 | H | -1.141 | -6.73002 | -2.18104 | H | 0.18995 | 6.31273 | 3.191901 |
| O | 0.201175 | 2.81039 | -1.13488 | H | -1.66085 | -6.58922 | -5.67523 | H | 1.279984 | 7.361415 | 4.121982 |
| H | 0.380301 | -3.58598 | -1.06308 | H | -1.82264 | -4.82353 | -5.72643 |  |  |  |  |

| Conformer **2Ac** | | | | | | | | | | | |
| --- | --- | --- | --- | --- | --- | --- | --- | --- | --- | --- | --- |
| C | -2.97633 | -3.13063 | -2.34996 | C | -3.26613 | -2.02768 | 0.516512 | H | -2.32153 | -7.06992 | -0.88397 |
| C | -2.34464 | -4.47764 | -1.96435 | C | 3.004172 | 6.572935 | 0.445794 | H | -4.24228 | -4.8929 | -4.08899 |
| O | -2.13073 | -4.52076 | -0.53583 | C | 2.531616 | 5.154488 | 0.660773 | H | -2.70672 | -5.71569 | -4.45931 |
| C | -1.24113 | -3.48852 | -0.10367 | C | 2.924039 | 4.142193 | 1.535446 | H | -4.17123 | -6.63767 | -4.05963 |
| C | -1.91327 | -2.09718 | -0.2335 | C | 1.244961 | 3.364394 | 0.236568 | H | -0.46262 | -0.89525 | 3.790637 |
| C | -2.21909 | -1.93549 | -1.75433 | C | 3.990179 | 4.17455 | 2.577075 | H | -1.55282 | 0.038577 | 2.782925 |
| C | -0.77923 | -3.7911 | 1.318501 | N | 1.506282 | 4.655997 | -0.11488 | H | -1.87502 | -1.67636 | 3.088537 |
| C | 0.256028 | -2.76026 | 1.749888 | C | 2.73623 | 7.433272 | 1.683444 | H | -3.98785 | -2.76282 | 0.147921 |
| C | -0.23167 | -1.2989 | 1.658414 | C | 4.481579 | 6.654113 | 0.022545 | H | -3.17232 | -2.22215 | 1.584655 |
| C | -0.9645 | -1.01612 | 0.332282 | C | 4.758718 | 5.98203 | -1.31718 | H | -3.72316 | -1.0373 | 0.403795 |
| O | 0.983298 | -0.5159 | 1.804441 | O | 2.815764 | 1.323115 | 2.749373 | H | 2.409589 | 7.01166 | -0.36852 |
| C | 1.021242 | 0.756992 | 1.254646 | H | -3.00111 | -3.02214 | -3.44022 | H | 4.457098 | 5.156661 | 2.668624 |
| C | 0.189464 | 1.125638 | 0.25805 | H | -4.01774 | -3.0917 | -2.01173 | H | 3.572595 | 3.917415 | 3.55602 |
| C | -0.73583 | 0.156788 | -0.2891 | H | -1.37145 | -4.57714 | -2.46397 | H | 4.776846 | 3.452781 | 2.334351 |
| C | 0.25566 | 2.504189 | -0.34174 | H | -2.80557 | -1.02737 | -1.94255 | H | 1.015366 | 5.161037 | -0.84377 |
| C | -3.22318 | -5.70661 | -2.33858 | H | -1.27701 | -1.82397 | -2.30872 | H | 2.976683 | 8.482779 | 1.481503 |
| O | -4.44248 | -5.65255 | -1.58367 | H | -0.33543 | -4.79398 | 1.352327 | H | 1.680887 | 7.383339 | 1.974451 |
| C | -2.51706 | -7.01562 | -1.96064 | H | -1.62447 | -3.82624 | 2.013982 | H | 3.333068 | 7.117376 | 2.544208 |
| C | -3.59589 | -5.73535 | -3.82114 | H | 1.152675 | -2.88013 | 1.124711 | H | 4.770812 | 7.709312 | -0.06189 |
| C | -1.08719 | -0.948 | 2.890165 | H | 0.593628 | -2.98255 | 2.770667 | H | 5.13651 | 6.213711 | 0.781742 |
| C | 2.06867 | 1.67825 | 1.845099 | H | -1.22694 | 0.430728 | -1.2187 | H | 5.803574 | 6.136871 | -1.60499 |
| C | 2.099477 | 3.005947 | 1.252541 | H | -4.16657 | -5.46606 | -0.66442 | H | 4.583668 | 4.902981 | -1.27018 |
| O | -0.4852 | 2.850095 | -1.25568 | H | -3.14873 | -7.88 | -2.19649 | H | 4.125609 | 6.401793 | -2.10533 |
| H | -0.345 | -3.5262 | -0.74189 | H | -1.56684 | -7.12614 | -2.49306 |  |  |  |  |

| Conformer **2Ad** | | | | | | | | | | | |
| --- | --- | --- | --- | --- | --- | --- | --- | --- | --- | --- | --- |
| C | -1.85847 | -3.00495 | -3.40868 | C | -3.12964 | -2.10217 | -0.75057 | H | -0.58664 | -5.54694 | -5.28865 |
| C | -1.37446 | -4.37156 | -2.91025 | C | 2.58587 | 6.48961 | 1.863396 | H | -3.77669 | -5.7336 | -2.37252 |
| O | -1.64265 | -4.52363 | -1.49812 | C | 2.099911 | 5.060222 | 1.816261 | H | -4.06331 | -4.88367 | -3.90683 |
| C | -0.98145 | -3.51765 | -0.72572 | C | 2.191364 | 3.993361 | 2.70913 | H | -3.89258 | -6.6462 | -3.86251 |
| C | -1.60046 | -2.12049 | -0.9935 | C | 1.076765 | 3.301336 | 0.867879 | H | -1.64525 | -1.18405 | 3.350649 |
| C | -1.37008 | -1.85646 | -2.51456 | C | 2.835112 | 3.958092 | 4.053407 | H | -2.34742 | -0.18911 | 2.088433 |
| C | -1.02702 | -3.91176 | 0.747478 | N | 1.413891 | 4.613001 | 0.706847 | H | -2.7121 | -1.9212 | 2.160684 |
| C | -0.22655 | -2.91026 | 1.57 | C | 1.891811 | 7.267674 | 2.9844 | H | -3.66349 | -2.81571 | -1.38542 |
| C | -0.68837 | -1.44687 | 1.406905 | C | 4.116755 | 6.597992 | 1.977874 | H | -3.40237 | -2.36218 | 0.271925 |
| C | -0.92899 | -1.078 | -0.07037 | C | 4.850179 | 6.014499 | 0.775792 | H | -3.54452 | -1.10823 | -0.95642 |
| O | 0.383557 | -0.67348 | 2.009728 | O | 1.734401 | 1.102356 | 3.639303 | H | 2.296015 | 6.980424 | 0.923072 |
| C | 0.577814 | 0.632772 | 1.585783 | H | -1.51341 | -2.83042 | -4.43397 | H | 3.222451 | 4.931691 | 4.358373 |
| C | 0.129872 | 1.065212 | 0.388639 | H | -2.95182 | -2.97409 | -3.44198 | H | 2.112599 | 3.640179 | 4.812178 |
| C | -0.52839 | 0.13346 | -0.50171 | H | -0.28423 | -4.43571 | -3.03428 | H | 3.67178 | 3.251905 | 4.052708 |
| C | 0.36476 | 2.480366 | -0.06598 | H | -1.87278 | -0.93469 | -2.83345 | H | 1.191615 | 5.164214 | -0.11417 |
| C | -2.00442 | -5.59062 | -3.64135 | H | -0.29731 | -1.71378 | -2.70436 | H | 2.163418 | 8.32808 | 2.94159 |
| O | -1.41909 | -6.77583 | -3.07088 | H | -0.59818 | -4.9142 | 0.870397 | H | 0.801963 | 7.198969 | 2.892532 |
| C | -1.66995 | -5.60259 | -5.13272 | H | -2.05824 | -3.99322 | 1.107108 | H | 2.165215 | 6.896473 | 3.976559 |
| C | -3.51851 | -5.70747 | -3.43683 | H | 0.832423 | -2.98742 | 1.283978 | H | 4.39393 | 7.656414 | 2.062404 |
| C | -1.9223 | -1.1794 | 2.289071 | H | -0.25318 | -3.19887 | 2.628953 | H | 4.482183 | 6.109024 | 2.887072 |
| C | 1.336738 | 1.514533 | 2.555834 | H | -0.67595 | 0.467759 | -1.52479 | H | 5.926752 | 6.187641 | 0.87351 |
| C | 1.539248 | 2.877801 | 2.09167 | H | -1.47755 | -6.65003 | -2.10275 | H | 4.693624 | 4.934696 | 0.693857 |
| O | -0.0281 | 2.885166 | -1.1549 | H | -1.99955 | -6.53906 | -5.5977 | H | 4.515601 | 6.484729 | -0.15434 |
| H | 0.079697 | -3.5118 | -1.01837 | H | -2.14184 | -4.77229 | -5.66674 |  |  |  |  |

| Conformer **2Ae** | | | | | | | | | | | |
| --- | --- | --- | --- | --- | --- | --- | --- | --- | --- | --- | --- |
| C | -2.77081 | -3.36079 | -2.30772 | C | -3.06865 | -2.20233 | 0.535946 | H | -2.1032 | -7.26776 | -0.76309 |
| C | -2.13459 | -4.69749 | -1.89466 | C | 3.207574 | 6.409931 | 0.339221 | H | -4.02778 | -5.16195 | -4.01309 |
| O | -1.92248 | -4.71148 | -0.4653 | C | 2.701505 | 5.000271 | 0.544191 | H | -2.48864 | -5.98609 | -4.36506 |
| C | -1.03735 | -3.66753 | -0.05254 | C | 3.090713 | 4.011361 | 1.444275 | H | -3.9502 | -6.90551 | -3.94906 |
| C | -1.71452 | -2.28159 | -0.2108 | C | 1.421662 | 3.200769 | 0.155163 | H | -0.27387 | -0.99464 | 3.790574 |
| C | -2.01887 | -2.15123 | -1.73493 | C | 4.152444 | 4.092458 | 2.487603 | H | -1.36618 | -0.08508 | 2.763141 |
| C | -0.57626 | -3.94011 | 1.375952 | N | 1.67774 | 4.486906 | -0.22215 | H | -1.68238 | -1.79483 | 3.102291 |
| C | 0.45453 | -2.89702 | 1.788151 | C | 3.692465 | 6.608439 | -1.10058 | H | -3.78711 | -2.94737 | 0.181078 |
| C | -0.03852 | -1.43961 | 1.667075 | C | 2.12772 | 7.43024 | 0.748444 | H | -2.97557 | -2.37523 | 1.607857 |
| C | -0.77058 | -1.18595 | 0.33467 | C | 2.627588 | 8.868124 | 0.760983 | H | -3.52925 | -1.21612 | 0.40302 |
| O | 1.173312 | -0.6493 | 1.799086 | O | 2.999949 | 1.213804 | 2.707581 | H | 4.076878 | 6.559927 | 0.990999 |
| C | 1.207241 | 0.612613 | 1.224261 | H | -2.79451 | -3.27403 | -3.39995 | H | 4.513955 | 5.114508 | 2.628075 |
| C | 0.37546 | 0.958309 | 0.219435 | H | -3.81282 | -3.31911 | -1.97166 | H | 3.768654 | 3.747784 | 3.453252 |
| C | -0.54545 | -0.02474 | -0.30956 | H | -1.16036 | -4.80319 | -2.39096 | H | 5.004991 | 3.465498 | 2.207779 |
| C | 0.437197 | 2.324884 | -0.40779 | H | -2.60849 | -1.24923 | -1.94187 | H | 1.185741 | 4.976701 | -0.96062 |
| C | -3.008 | -5.93696 | -2.24559 | H | -1.07646 | -2.04716 | -2.2902 | H | 4.206954 | 7.567589 | -1.2155 |
| O | -4.22851 | -5.87258 | -1.49346 | H | -0.12876 | -4.94044 | 1.430234 | H | 4.400116 | 5.822053 | -1.3873 |
| C | -2.29747 | -7.23554 | -1.84088 | H | -1.42231 | -3.96465 | 2.0709 | H | 2.861646 | 6.585208 | -1.81444 |
| C | -3.37859 | -5.99648 | -3.72777 | H | 1.352477 | -3.02588 | 1.166638 | H | 1.261172 | 7.360836 | 0.07883 |
| C | -0.89701 | -1.06757 | 2.890525 | H | 0.791561 | -3.09775 | 2.813575 | H | 1.761787 | 7.186925 | 1.75457 |
| C | 2.250655 | 1.549141 | 1.797609 | H | -1.0363 | 0.228827 | -1.24505 | H | 1.848402 | 9.532262 | 1.148853 |
| C | 2.275624 | 2.865489 | 1.180632 | H | -3.95455 | -5.66682 | -0.57774 | H | 3.509727 | 8.972362 | 1.400424 |
| O | -0.30335 | 2.649291 | -1.32985 | H | -2.92556 | -8.1068 | -2.06036 | H | 2.884173 | 9.212864 | -0.24485 |
| H | -0.14021 | -3.71447 | -0.68873 | H | -1.34612 | -7.35298 | -2.36979 |  |  |  |  |

**Fig. S81.** Correlations between calculated and experimental ^13^C NMR chemical shifts of **4A** and **4B**


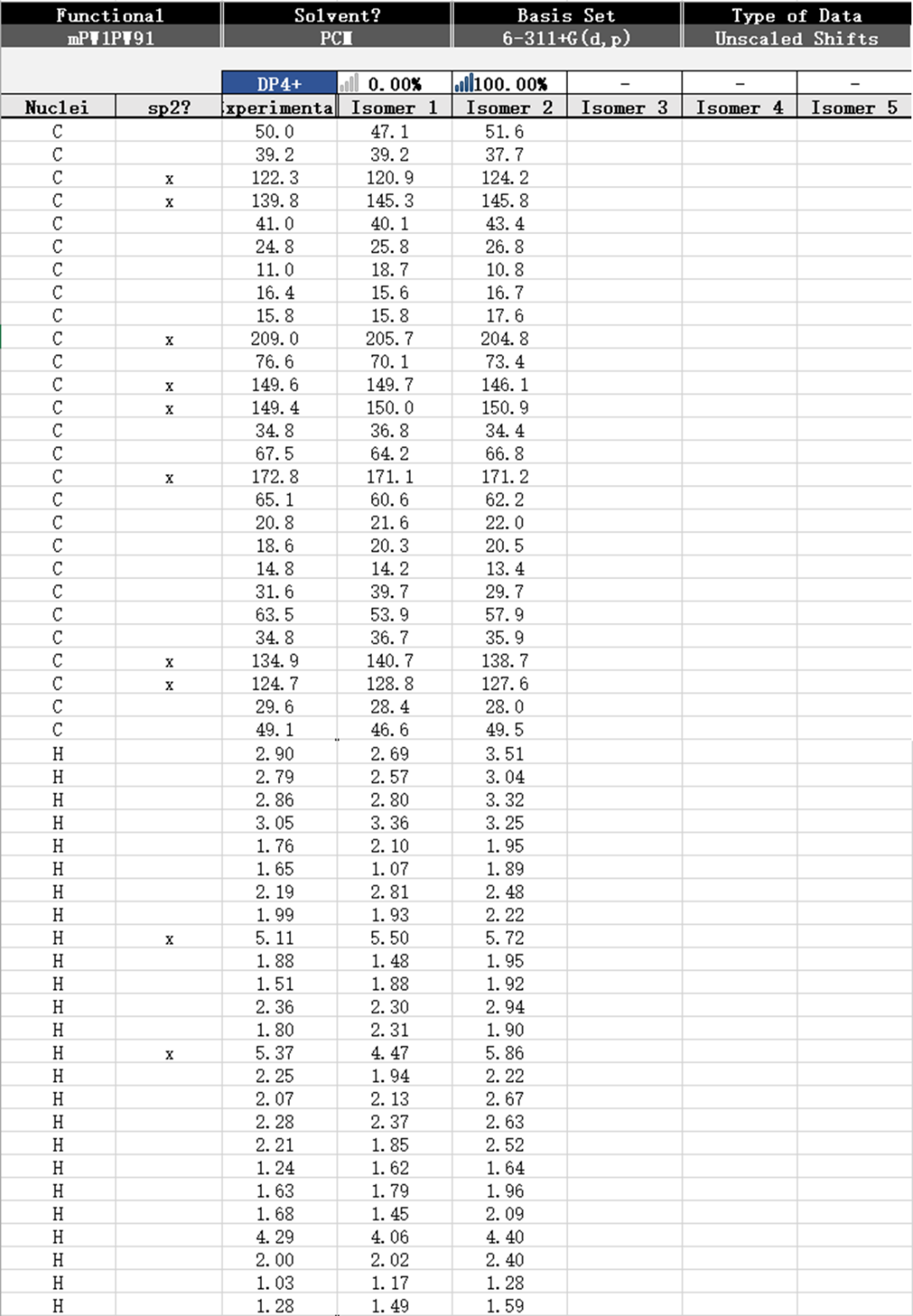


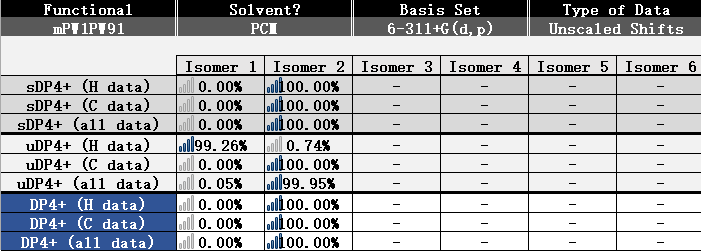


**Fig. S82.** DP4+ analysis results of **4**

**Table S5.** Energy analysis for conformers of **4Aa**~**4Ae** at mpw1pw91/6-31+g(d,p) level in the gas phase

| Temperature: 298.15 | | Q（relat）: 1.083485 | |
| --- | --- | --- | --- |
| conformer | ΔG (kcal/mol)^a^ | Qi(Relat) | Population^b^ |
| **4Aa** | 0 | 0.301427 | 30.14% |
| **4Ab** | 0.035141 | 0.284058 | 28.41% |
| **4Ac** | 0.363956 | 0.163014 | 16.30% |
| **4Ad** | 0.557856 | 0.117490 | 11.75% |
| **4Ae** | 1.142696 | 0.043755 | 4.38% |
| **4Af** | 1.226782 | 0.037962 | 3.80% |
| **4Ag** | 1.436998 | 0.026617 | 2.66% |
| **4Ah** | 1.458333 | 0.025675 | 2.57% |

^a^The relative Gibbs free energy; ^b^The Boltzmann distribution of each conformer.

**Table S6.** Cartesian coordinates for the low-energy optimized conformers of **4A** at M062X/def2svp level.

| Conformer **4Aa** | | | | | | | | | | | |
| --- | --- | --- | --- | --- | --- | --- | --- | --- | --- | --- | --- |
| C | 2.751548 | -2.282077 | 0.049366 | O | 3.051726 | -3.416193 | 0.83302 | H | 4.418448 | 3.170719 | -0.236813 |
| C | 1.250706 | -2.168234 | -0.160806 | H | -3.10704 | -1.558732 | -1.30148 | H | 1.06796 | -4.29202 | -0.334089 |
| C | 3.535101 | -2.389922 | -1.253394 | C | -2.814589 | -2.54464 | 1.33343 | H | -0.490737 | -3.429035 | -0.407566 |
| C | 0.677311 | -0.817189 | -0.287604 | O | -2.748397 | -3.279291 | 0.380389 | H | 0.611215 | -3.409084 | -1.813047 |
| C | 3.756041 | -1.035042 | -1.942573 | C | -1.970317 | -2.65697 | 2.568518 | H | 5.535314 | -1.270862 | 0.375496 |
| C | 4.454464 | -0.043621 | -1.038404 | O | -4.682571 | 2.198905 | -0.771149 | H | 6.339904 | -1.071446 | -1.185358 |
| C | 3.911602 | 1.153218 | -0.769234 | H | -1.547561 | -0.015655 | 0.882192 | H | 6.352927 | 0.25986 | -0.006919 |
| C | -0.64214 | -0.544335 | -0.962215 | C | -0.244983 | 2.360962 | -1.241321 | H | 1.010098 | 4.519431 | 0.767634 |
| C | -1.528053 | 0.401656 | -0.141644 | C | -4.804091 | -0.515142 | -2.087367 | H | 2.727863 | 4.744902 | 1.181127 |
| C | -1.074344 | 1.892817 | -0.037406 | O | 0.607575 | -1.522025 | 0.944021 | H | 2.273302 | 4.099293 | -0.401117 |
| C | -0.404806 | 2.293262 | 1.300085 | H | 1.385971 | 0.02035 | -0.258664 | H | -5.010027 | -2.24584 | 0.020011 |
| C | 1.037535 | 1.90507 | 1.478633 | H | 3.060096 | -1.361887 | 0.5806 | H | -5.257661 | -0.565162 | 0.609417 |
| C | 2.101911 | 2.684 | 1.222782 | H | 4.502682 | -2.84946 | -1.003672 | H | 2.564455 | -3.334601 | 1.661974 |
| C | 3.509371 | 2.198604 | 1.479862 | H | 3.032771 | -3.090187 | -1.938332 | H | -1.786307 | -3.717022 | 2.776918 |
| C | 4.401575 | 2.168508 | 0.221573 | H | 4.368977 | -1.2058 | -2.843846 | H | -2.444935 | -2.160567 | 3.421115 |
| C | 0.562416 | -3.394127 | -0.714172 | H | 2.797232 | -0.614276 | -2.285599 | H | -1.005032 | -2.177221 | 2.345735 |
| C | 5.73897 | -0.543711 | -0.428404 | H | 2.965345 | 1.400055 | -1.267125 | H | -4.628729 | 3.156645 | -0.611696 |
| C | 2.011226 | 4.083189 | 0.669814 | H | -0.465553 | -0.147945 | -1.974185 | H | -0.751816 | 2.118394 | -2.186879 |
| C | -2.391953 | 2.65054 | -0.136864 | H | -1.166006 | -1.501935 | -1.076447 | H | -0.120262 | 3.451955 | -1.191986 |
| C | -3.447728 | 1.719426 | -0.552725 | H | -0.51882 | 3.384243 | 1.380944 | H | 0.751514 | 1.897771 | -1.243131 |
| C | -2.978499 | 0.458437 | -0.619558 | H | -1.002123 | 1.857288 | 2.117194 | H | -5.312922 | -1.453522 | -2.350666 |
| O | -2.580139 | 3.832079 | 0.048806 | H | 1.226122 | 0.895152 | 1.862014 | H | -5.556856 | 0.224705 | -1.786342 |
| C | -3.785652 | -0.76227 | -0.971337 | H | 3.481378 | 1.191022 | 1.923389 | H | -4.301722 | -0.135413 | -2.987411 |
| C | -4.512592 | -1.295837 | 0.267718 | H | 3.977106 | 2.869601 | 2.221614 |  |  |  |  |
| O | -3.649452 | -1.496722 | 1.384414 | H | 5.433342 | 1.952833 | 0.52847 |  |  |  |  |
|  |  |  |  |  |  |  |  |  |  |  |  |

| Conformer **4Ab** | | | | | | | | | | | |
| --- | --- | --- | --- | --- | --- | --- | --- | --- | --- | --- | --- |
| C | -3.177054 | -2.245118 | -0.465772 | O | -3.637631 | -3.266104 | -1.322635 | H | -4.07672 | 3.263136 | 1.142468 |
| C | -1.657646 | -2.254207 | -0.437374 | H | 4.319602 | -1.255085 | -1.669273 | H | -0.984091 | -3.601806 | 1.117708 |
| C | -3.808804 | -2.454447 | 0.901876 | C | 3.903084 | -0.94162 | 1.870868 | H | -1.619825 | -4.391053 | -0.353456 |
| C | -0.950626 | -0.965947 | -0.385005 | O | 2.72773 | -0.969467 | 2.12614 | H | -0.003537 | -3.643311 | -0.37046 |
| C | -3.692211 | -1.225268 | 1.812311 | C | 4.926733 | -0.089281 | 2.563163 | H | -5.870183 | -0.784629 | -0.112369 |
| C | -4.36864 | -0.001838 | 1.233009 | O | 4.829743 | 1.103268 | -0.65545 | H | -6.336115 | -0.796426 | 1.592298 |
| C | -3.703062 | 1.153071 | 1.080377 | H | 0.852055 | 0.094739 | -1.780069 | H | -6.349596 | 0.729877 | 0.682023 |
| C | 0.466352 | -0.828638 | 0.098648 | C | 0.695672 | 1.893195 | 1.116773 | H | -0.682759 | 4.57972 | 0.115758 |
| C | 1.261711 | 0.178382 | -0.755745 | C | 2.489968 | -2.211527 | -2.284844 | H | -2.411885 | 5.008858 | 0.087461 |
| C | 1.191455 | 1.679339 | -0.317396 | O | -1.111036 | -1.661589 | -1.616955 | H | -1.799884 | 3.863522 | 1.289167 |
| C | 0.422377 | 2.578747 | -1.313402 | H | -1.572686 | -0.063631 | -0.308737 | H | 4.161634 | -3.245161 | -0.331194 |
| C | -1.062059 | 2.35029 | -1.3621 | H | -3.479472 | -1.253744 | -0.855196 | H | 2.694681 | -2.658784 | 0.508143 |
| C | -2.000715 | 3.046384 | -0.700433 | H | -4.865793 | -2.708803 | 0.733344 | H | -3.226421 | -3.120497 | -2.183981 |
| C | -3.468958 | 2.734153 | -0.870639 | H | -3.359294 | -3.334465 | 1.386934 | H | 4.492734 | 0.354378 | 3.463957 |
| C | -4.20442 | 2.417658 | 0.445924 | H | -4.162207 | -1.470073 | 2.780143 | H | 5.230472 | 0.695399 | 1.85532 |
| C | -1.023505 | -3.547229 | 0.019941 | H | -2.633575 | -1.001483 | 2.021559 | H | 5.817571 | -0.682757 | 2.802021 |
| C | -5.805349 | -0.209838 | 0.826221 | H | -2.656692 | 1.177359 | 1.411625 | H | 4.998267 | 2.053895 | -0.540696 |
| C | -1.697679 | 4.184314 | 0.239293 | H | 0.493922 | -0.564803 | 1.162541 | H | 1.279679 | 1.290256 | 1.827712 |
| C | 2.647788 | 2.12299 | -0.334775 | H | 0.943355 | -1.810208 | 0.030426 | H | 0.812839 | 2.954124 | 1.38064 |
| C | 3.493472 | 0.960791 | -0.624896 | H | 0.656416 | 3.61982 | -1.046584 | H | -0.366069 | 1.621902 | 1.209962 |
| C | 2.746843 | -0.133252 | -0.860187 | H | 0.847202 | 2.409245 | -2.316789 | H | 2.934165 | -3.191969 | -2.510593 |
| O | 3.082683 | 3.238299 | -0.150205 | H | -1.403867 | 1.53261 | -2.008111 | H | 1.449245 | -2.365194 | -1.964008 |
| C | 3.326914 | -1.468854 | -1.240262 | H | -3.591754 | 1.889101 | -1.566078 | H | 2.45643 | -1.636055 | -3.220333 |
| C | 3.61143 | -2.350092 | -0.011868 | H | -3.954979 | 3.610754 | -1.334366 |  |  |  |  |
| O | 4.467106 | -1.66366 | 0.890549 | H | -5.280033 | 2.352332 | 0.237296 |  |  |  |  |
|  |  |  |  |  |  |  |  |  |  |  |  |

| Conformer **4Ac** | | | | | | | | | | | |
| --- | --- | --- | --- | --- | --- | --- | --- | --- | --- | --- | --- |
| C | 3.164501 | -2.360567 | 0.355736 | O | 3.386624 | -3.510195 | 1.142518 | H | 4.402334 | 3.287934 | 0.364123 |
| C | 1.740245 | -2.367994 | -0.174907 | H | -2.519145 | -2.23595 | -0.500285 | H | 1.642663 | -3.51746 | -2.009909 |
| C | 4.222781 | -2.324355 | -0.738086 | C | -4.633727 | -0.793971 | 2.379162 | H | 1.822381 | -4.478856 | -0.517199 |
| C | 1.04632 | -1.079758 | -0.32191 | O | -5.81036 | -1.010572 | 2.301914 | H | 0.250969 | -3.740379 | -0.914477 |
| C | 4.409268 | -0.92915 | -1.350084 | C | -3.905772 | -0.291862 | 3.596355 | H | 5.706114 | -1.026696 | 1.276267 |
| C | 4.789505 | 0.110556 | -0.318189 | O | -4.431515 | 1.13977 | -1.903935 | H | 6.777074 | -0.679799 | -0.086037 |
| C | 4.077576 | 1.238877 | -0.177997 | H | -1.155647 | -0.161229 | 0.508897 | H | 6.388918 | 0.602576 | 1.081599 |
| C | -0.144036 | -0.871765 | -1.221594 | C | 0.126818 | 1.992344 | -1.799219 | H | 0.83595 | 4.525653 | 0.124657 |
| C | -1.166726 | 0.075778 | -0.571168 | C | -3.79666 | -2.031665 | -2.237732 | H | 2.382129 | 4.86434 | 0.941242 |
| C | -0.93748 | 1.608154 | -0.767961 | O | 0.811455 | -1.90777 | 0.806936 | H | 2.363643 | 3.98583 | -0.593622 |
| C | -0.685396 | 2.338996 | 0.573646 | H | 1.624783 | -0.174732 | -0.087038 | H | -4.86667 | -2.38545 | 0.2827 |
| C | 0.652707 | 2.046617 | 1.19207 | H | 3.257941 | -1.450022 | 0.977704 | H | -5.141251 | -0.692089 | -0.198143 |
| C | 1.754376 | 2.810655 | 1.112605 | H | 5.163288 | -2.669647 | -0.28394 | H | 2.7196 | -3.51401 | 1.840406 |
| C | 3.037513 | 2.403781 | 1.79908 | H | 3.977569 | -3.056807 | -1.522596 | H | -3.502252 | 0.708887 | 3.389315 |
| C | 4.253337 | 2.31215 | 0.856141 | H | 5.205447 | -0.992636 | -2.111338 | H | -3.057413 | -0.950959 | 3.820877 |
| C | 1.336836 | -3.596974 | -0.956501 | H | 3.493272 | -0.613056 | -1.875003 | H | -4.598122 | -0.248445 | 4.442099 |
| C | 5.975602 | -0.253691 | 0.53752 | H | 3.232971 | 1.386593 | -0.863231 | H | -4.522529 | 2.093886 | -2.066165 |
| C | 1.82418 | 4.11378 | 0.359581 | H | 0.177717 | -0.498667 | -2.204589 | H | -0.093661 | 1.559761 | -2.78555 |
| C | -2.27603 | 2.099024 | -1.308735 | H | -0.619171 | -1.846122 | -1.395533 | H | 0.13525 | 3.086291 | -1.909145 |
| C | -3.177187 | 0.950945 | -1.462327 | H | -0.814854 | 3.414512 | 0.385217 | H | 1.126887 | 1.663101 | -1.482963 |
| C | -2.58389 | -0.179992 | -1.039898 | H | -1.485439 | 2.039712 | 1.271498 | H | -4.29876 | -3.001767 | -2.111351 |
| O | -2.591784 | 3.240409 | -1.560012 | H | 0.729865 | 1.106789 | 1.752663 | H | -4.5166 | -1.314413 | -2.656669 |
| C | -3.254046 | -1.518844 | -0.901461 | H | 2.897277 | 1.435718 | 2.304882 | H | -2.980934 | -2.157736 | -2.962967 |
| C | -4.374604 | -1.412892 | 0.126061 | H | 3.26348 | 3.150549 | 2.580589 |  |  |  |  |
| O | -3.791021 | -0.972541 | 1.348961 | H | 5.151678 | 2.132931 | 1.460832 |  |  |  |  |
|  |  |  |  |  |  |  |  |  |  |  |  |

| Conformer **4Ad** | | | | | | | | | | | |
| --- | --- | --- | --- | --- | --- | --- | --- | --- | --- | --- | --- |
| C | 3.099847 | -1.970955 | 0.687116 | O | 3.849603 | -2.95253 | 1.369413 | H | 4.137804 | 3.35414 | -0.327909 |
| C | 1.698613 | -2.506129 | 0.445134 | H | -3.446328 | -1.265743 | 1.603257 | H | 2.364573 | -4.380908 | -0.311286 |
| C | 3.85315 | -1.623936 | -0.588226 | C | -3.613891 | 1.500374 | 2.280738 | H | 0.605141 | -4.134949 | -0.447799 |
| C | 0.547705 | -1.666252 | 0.828475 | O | -3.661976 | 0.690742 | 3.165809 | H | 1.720099 | -3.295749 | -1.570945 |
| C | 5.158598 | -0.88588 | -0.314516 | C | -2.890418 | 2.818256 | 2.338535 | H | 7.109838 | 1.044612 | -0.358005 |
| C | 5.063902 | 0.566117 | 0.100611 | O | -4.428668 | -0.525904 | -2.351218 | H | 6.326644 | 2.24302 | 0.696765 |
| C | 3.917473 | 1.260433 | 0.120099 | H | -1.447986 | 0.158615 | 0.793058 | H | 6.821408 | 0.655319 | 1.340104 |
| C | -0.855304 | -1.826258 | 0.280657 | C | 0.364602 | -0.262023 | -1.923155 | H | 0.96393 | 3.225519 | -2.441186 |
| C | -1.523041 | -0.497085 | -0.09445 | C | -4.796739 | -2.329762 | 0.296411 | H | 2.113079 | 4.325119 | -1.63793 |
| C | -0.956405 | 0.236088 | -1.347167 | O | 1.015684 | -2.695696 | 1.678842 | H | 2.61147 | 2.681098 | -2.059921 |
| C | -0.88292 | 1.763943 | -1.064833 | H | 0.78085 | -0.654398 | 1.194776 | H | -5.564286 | -0.087234 | 1.766535 |
| C | 0.194063 | 2.137542 | -0.087965 | H | 3.002639 | -1.06698 | 1.317173 | H | -5.540473 | 0.273885 | 0.002296 |
| C | 1.336843 | 2.79796 | -0.33451 | H | 4.08081 | -2.571011 | -1.100894 | H | 3.357545 | -3.168297 | 2.172242 |
| C | 2.302396 | 3.098138 | 0.790412 | H | 3.20599 | -1.042358 | -1.263278 | H | -2.522875 | 2.986077 | 3.35516 |
| C | 3.757518 | 2.714827 | 0.488324 | H | 5.706405 | -1.439977 | 0.466848 | H | -3.563285 | 3.627185 | 2.027422 |
| C | 1.58116 | -3.64302 | -0.536628 | H | 5.80085 | -0.93253 | -1.210856 | H | -2.045901 | 2.803877 | 1.634091 |
| C | 6.391852 | 1.173047 | 0.467574 | H | 2.991353 | 0.749368 | -0.166057 | H | -4.250011 | -0.221899 | -3.257011 |
| C | 1.762749 | 3.282338 | -1.693618 | H | -0.877809 | -2.508302 | -0.583152 | H | 0.537237 | 0.236378 | -2.888686 |
| C | -2.067433 | 0.066729 | -2.377097 | H | -1.453207 | -2.306178 | 1.072455 | H | 1.201112 | -0.024275 | -1.252195 |
| C | -3.262998 | -0.442854 | -1.689831 | H | -0.751738 | 2.277941 | -2.026883 | H | 0.352183 | -1.346726 | -2.100367 |
| C | -2.990973 | -0.708797 | -0.400059 | H | -1.867543 | 2.06917 | -0.666968 | H | -5.324954 | -2.181156 | -0.655935 |
| O | -2.041042 | 0.370073 | -3.548165 | H | 0.028152 | 1.820647 | 0.949683 | H | -4.135803 | -3.20059 | 0.188104 |
| C | -3.984691 | -1.084505 | 0.660628 | H | 1.965828 | 2.583326 | 1.703863 | H | -5.534413 | -2.556311 | 1.079895 |
| C | -4.922609 | 0.104802 | 0.894034 | H | 2.26802 | 4.180974 | 1.002284 |  |  |  |  |
| O | -4.208068 | 1.324484 | 1.089442 | H | 4.371101 | 2.961086 | 1.368114 |  |  |  |  |
|  |  |  |  |  |  |  |  |  |  |  |  |

| Conformer **4Ae** | | | | | | | | | | | |
| --- | --- | --- | --- | --- | --- | --- | --- | --- | --- | --- | --- |
| C | 2.605417 | -1.968032 | -0.902611 | O | 3.149609 | -3.257562 | -1.083017 | H | 4.638357 | 2.410022 | 1.707333 |
| C | 1.134499 | -1.992671 | -1.274832 | H | -4.032182 | -1.300256 | -0.442565 | H | 1.451974 | -3.086591 | -3.076637 |
| C | 3.427712 | -1.000495 | -1.740203 | C | -3.006306 | -2.666975 | 1.737834 | H | -0.235021 | -2.55767 | -2.838737 |
| C | 0.165167 | -1.346039 | -0.370895 | O | -3.947421 | -3.221645 | 1.240047 | H | 1.012989 | -1.377451 | -3.342369 |
| C | 4.846878 | -0.83052 | -1.210324 | C | -1.729162 | -3.32815 | 2.169964 | H | 7.132077 | 0.277005 | -0.182041 |
| C | 5.0195 | -0.038628 | 0.067421 | O | -4.433683 | 2.390126 | -1.554367 | H | 6.563149 | 0.595798 | 1.472385 |
| C | 4.023588 | 0.629617 | 0.665752 | H | -1.542614 | 0.249363 | 0.964245 | H | 6.72343 | -1.076948 | 0.875486 |
| C | -1.218511 | -0.897755 | -0.79727 | C | 0.262736 | 1.547726 | -1.476611 | H | 1.562997 | 4.197119 | 0.35409 |
| C | -1.68218 | 0.396381 | -0.118342 | C | -5.64144 | 0.105503 | -0.295668 | H | 2.881921 | 4.283403 | 1.548536 |
| C | -0.951585 | 1.699539 | -0.562461 | O | 0.385169 | -2.747952 | -0.327676 | H | 3.058767 | 3.271349 | 0.108707 |
| C | -0.58254 | 2.565481 | 0.675442 | H | 0.580766 | -0.810893 | 0.496775 | H | -5.026862 | -1.170207 | 1.869937 |
| C | 0.519308 | 1.990034 | 1.516449 | H | 2.666363 | -1.679548 | 0.162895 | H | -4.091754 | 0.340042 | 2.122165 |
| C | 1.781798 | 2.434615 | 1.622424 | H | 3.475202 | -1.407341 | -2.762061 | H | 2.634975 | -3.86038 | -0.531735 |
| C | 2.758422 | 1.75078 | 2.553807 | H | 2.911772 | -0.02955 | -1.805862 | H | -1.008915 | -3.291114 | 1.337742 |
| C | 4.126125 | 1.455968 | 1.92351 | H | 5.277027 | -1.835415 | -1.059997 | H | -1.934888 | -4.378154 | 2.402553 |
| C | 0.815041 | -2.264044 | -2.721774 | H | 5.473415 | -0.355958 | -1.985092 | H | -1.295176 | -2.809762 | 3.032394 |
| C | 6.428009 | -0.052204 | 0.598574 | H | 3.02563 | 0.59704 | 0.213616 | H | -4.126506 | 3.206447 | -1.984768 |
| C | 2.334708 | 3.612814 | 0.867097 | H | -1.288022 | -0.7899 | -1.890936 | H | 0.554283 | 2.543929 | -1.840995 |
| C | -2.039445 | 2.471048 | -1.28775 | H | -1.910415 | -1.711835 | -0.530232 | H | 1.113641 | 1.113245 | -0.933951 |
| C | -3.331337 | 1.798091 | -1.071324 | H | -0.328489 | 3.570349 | 0.31088 | H | 0.044653 | 0.920485 | -2.352589 |
| C | -3.16242 | 0.644713 | -0.391757 | H | -1.49262 | 2.669437 | 1.291195 | H | -5.90879 | 1.035552 | 0.226823 |
| O | -1.926899 | 3.506716 | -1.904226 | H | 0.250844 | 1.112358 | 2.11746 | H | -5.744716 | 0.296867 | -1.369795 |
| C | -4.218808 | -0.332196 | 0.053261 | H | 2.310633 | 0.815541 | 2.924807 | H | -6.35373 | -0.67987 | -0.007471 |
| C | -4.136515 | -0.604306 | 1.561973 | H | 2.916415 | 2.399589 | 3.432862 |  |  |  |  |
| O | -2.974864 | -1.335174 | 1.947175 | H | 4.752186 | 0.946054 | 2.671323 |  |  |  |  |
|  |  |  |  |  |  |  |  |  |  |  |  |

| Conformer **4Af** | | | | | | | | | | | |
| --- | --- | --- | --- | --- | --- | --- | --- | --- | --- | --- | --- |
| C | -2.64917 | 2.470846 | 0.262221 | O | -2.889696 | 3.636558 | 1.018226 | H | -4.590715 | -2.390942 | -0.378685 |
| C | -1.165861 | 2.321346 | -0.033995 | H | 3.131656 | 1.144293 | -1.474628 | H | -0.837543 | 4.425534 | -0.182075 |
| C | -3.526879 | 2.55117 | -0.981071 | C | 3.030498 | 2.540277 | 1.00871 | H | 0.610247 | 3.449546 | -0.557332 |
| C | -0.652783 | 0.946665 | -0.137614 | O | 3.006549 | 3.13227 | -0.040924 | H | -0.729936 | 3.566304 | -1.735712 |
| C | -3.362316 | 1.397221 | -1.968283 | C | 2.263462 | 2.928948 | 2.237568 | H | -3.42043 | -2.092621 | -2.059349 |
| C | -3.700855 | -0.002837 | -1.49244 | O | 4.284673 | -2.688274 | -0.559439 | H | -3.867981 | -0.998471 | -3.390647 |
| C | -4.344216 | -0.234513 | -0.339549 | H | 1.526731 | 0.004513 | 0.998894 | H | -2.227297 | -0.961265 | -2.741292 |
| C | 0.620736 | 0.558629 | -0.836545 | C | -0.086362 | -2.301948 | -0.999532 | H | -1.498834 | -4.220201 | 0.935188 |
| C | 1.410643 | -0.455287 | 0.000238 | C | 4.706367 | -0.158148 | -2.116144 | H | -3.225737 | -4.363799 | 1.347551 |
| C | 0.765144 | -1.864605 | 0.201413 | O | -0.491815 | 1.69232 | 1.062844 | H | -2.738632 | -3.759838 | -0.241579 |
| C | 0.019531 | -2.101856 | 1.540416 | H | -1.400793 | 0.149154 | -0.051869 | H | 5.128678 | 1.812536 | -0.284287 |
| C | -1.404559 | -1.621169 | 1.658676 | H | -2.916692 | 1.577077 | 0.850953 | H | 5.218104 | 0.217422 | 0.539543 |
| C | -2.507318 | -2.333648 | 1.369967 | H | -4.571391 | 2.635774 | -0.643532 | H | -2.39354 | 3.556026 | 1.841537 |
| C | -3.893715 | -1.776126 | 1.598047 | H | -3.297762 | 3.499859 | -1.491817 | H | 2.761193 | 2.568569 | 3.144 |
| C | -4.709388 | -1.539268 | 0.305965 | H | -3.9713 | 1.608632 | -2.864773 | H | 1.265807 | 2.47035 | 2.157591 |
| C | -0.480627 | 3.505784 | -0.665308 | H | -2.320243 | 1.381648 | -2.336377 | H | 2.145074 | 4.018072 | 2.253159 |
| C | -3.285064 | -1.080212 | -2.458509 | H | -4.632849 | 0.634603 | 0.260029 | H | 4.114913 | -3.61329 | -0.312512 |
| C | -2.479598 | -3.741147 | 0.830068 | H | 0.390871 | 0.158141 | -1.836908 | H | 0.448249 | -2.128987 | -1.945382 |
| C | 1.981412 | -2.782696 | 0.174619 | H | 1.231286 | 1.460362 | -0.9764 | H | -0.301733 | -3.376746 | -0.923146 |
| C | 3.131223 | -2.034015 | -0.349812 | H | 0.05614 | -3.188492 | 1.710484 | H | -1.041941 | -1.76126 | -1.022107 |
| C | 2.818887 | -0.734882 | -0.522812 | H | 0.619288 | -1.644338 | 2.343096 | H | 4.153974 | -0.619161 | -2.946144 |
| O | 2.029065 | -3.955884 | 0.470046 | H | -1.542485 | -0.603909 | 2.043519 | H | 5.28927 | 0.68274 | -2.518421 |
| C | 3.738018 | 0.339048 | -1.039592 | H | -3.828811 | -0.831107 | 2.160519 | H | 5.398107 | -0.911018 | -1.716999 |
| C | 4.542648 | 0.965698 | 0.104044 | H | -4.444475 | -2.488254 | 2.235589 |  |  |  |  |
| O | 3.737453 | 1.415383 | 1.190808 | H | -5.777444 | -1.506086 | 0.573352 |  |  |  |  |
|  |  |  |  |  |  |  |  |  |  |  |  |

| Conformer **4Ag** | | | | | | | | | | | |
| --- | --- | --- | --- | --- | --- | --- | --- | --- | --- | --- | --- |
| C | -3.266825 | -1.826244 | -0.3745 | O | -4.162575 | -2.700864 | -1.024233 | H | -4.011583 | 3.817656 | 0.305689 |
| C | -1.908652 | -2.493596 | -0.240235 | H | 3.18867 | -1.813154 | -1.78576 | H | -2.641833 | -4.292208 | 0.635689 |
| C | -3.877688 | -1.413248 | 0.951685 | C | 3.812124 | 1.782008 | -1.456556 | H | -0.863678 | -4.173417 | 0.609318 |
| C | -0.740018 | -1.75565 | -0.751618 | O | 4.281566 | 2.039896 | -0.38244 | H | -1.810831 | -3.221445 | 1.794677 |
| C | -5.14428 | -0.565333 | 0.783609 | C | 2.918554 | 2.695997 | -2.251527 | H | -5.191184 | -0.442037 | -1.913652 |
| C | -4.91113 | 0.649642 | -0.095618 | O | 4.553633 | -0.542624 | 1.888398 | H | -6.565249 | 0.56819 | -1.469126 |
| C | -4.21218 | 1.695239 | 0.368959 | H | 1.339574 | -0.059321 | -1.000626 | H | -5.109201 | 1.327541 | -2.165796 |
| C | 0.691254 | -1.974833 | -0.314756 | C | -0.237152 | -0.278508 | 1.913041 | H | -0.591298 | 3.66151 | 1.984382 |
| C | 1.448574 | -0.660163 | -0.077559 | C | 4.615926 | -2.704236 | -0.430561 | H | -1.673902 | 4.721878 | 1.05338 |
| C | 1.005024 | 0.187468 | 1.157814 | O | -1.355381 | -2.77189 | -1.522102 | H | -2.341256 | 3.357808 | 1.955248 |
| C | 0.85363 | 1.687295 | 0.783154 | H | -0.942663 | -0.744123 | -1.13168 | H | 5.423507 | -0.8212 | -2.226307 |
| C | -0.324579 | 1.969937 | -0.102723 | H | -3.109898 | -0.918443 | -0.989529 | H | 5.345192 | 0.018301 | -0.637213 |
| C | -1.343869 | 2.815068 | 0.11974 | H | -4.109602 | -2.319636 | 1.533166 | H | -3.726651 | -2.986286 | -1.838379 |
| C | -2.428876 | 2.99239 | -0.919665 | H | -3.127595 | -0.839072 | 1.517781 | H | 3.171058 | 2.652124 | -3.317483 |
| C | -3.866276 | 2.958658 | -0.369342 | H | -5.931633 | -1.194058 | 0.34246 | H | 1.876915 | 2.359177 | -2.136593 |
| C | -1.790514 | -3.608059 | 0.765368 | H | -5.495326 | -0.252201 | 1.778061 | H | 3.006964 | 3.715658 | -1.86374 |
| C | -5.46357 | 0.538484 | -1.491249 | H | -3.843306 | 1.627989 | 1.401393 | H | 4.491377 | -0.075649 | 2.738494 |
| C | -1.480881 | 3.677317 | 1.345883 | H | 0.750865 | -2.608356 | 0.583528 | H | -0.19702 | -1.349059 | 2.159177 |
| C | 2.22137 | 0.125751 | 2.074968 | H | 1.194258 | -2.530653 | -1.122222 | H | -0.302219 | 0.283368 | 2.856665 |
| C | 3.333007 | -0.496451 | 1.340179 | H | 0.794497 | 2.2525 | 1.72316 | H | -1.144755 | -0.079971 | 1.326754 |
| C | 2.92731 | -0.917944 | 0.129914 | H | 1.789531 | 2.005973 | 0.291515 | H | 3.937899 | -3.500418 | -0.093829 |
| O | 2.318714 | 0.566294 | 3.196927 | H | -0.345672 | 1.419909 | -1.052645 | H | 5.255421 | -3.111187 | -1.227366 |
| C | 3.816098 | -1.500482 | -0.935142 | H | -2.319307 | 2.218723 | -1.69609 | H | 5.249652 | -2.415547 | 0.419233 |
| C | 4.753382 | -0.410941 | -1.457857 | H | -2.278271 | 3.969159 | -1.414057 |  |  |  |  |
| O | 3.991607 | 0.61369 | -2.095748 | H | -4.560532 | 3.116565 | -1.206515 |  |  |  |  |
|  |  |  |  |  |  |  |  |  |  |  |  |

| Conformer **4Ah** | | | | | | | | | | | |
| --- | --- | --- | --- | --- | --- | --- | --- | --- | --- | --- | --- |
| C | 3.15366 | -2.487532 | 0.306951 | O | 3.514064 | -3.589269 | 1.109896 | H | 4.735968 | 2.984288 | -0.825886 |
| C | 1.638817 | -2.376998 | 0.242613 | H | -4.218776 | -0.66493 | 1.544728 | H | 0.904776 | -3.632099 | -1.362612 |
| C | 3.810729 | -2.659607 | -1.054846 | C | -5.679312 | -1.015801 | -0.815451 | H | 1.427333 | -4.500811 | 0.10575 |
| C | 1.038573 | -1.034905 | 0.206955 | O | -6.258936 | -1.378 | 0.170068 | H | -0.121376 | -3.621834 | 0.09696 |
| C | 3.876049 | -1.355721 | -1.860936 | C | -6.267934 | -0.213776 | -1.940203 | H | 6.539654 | -1.228501 | -1.466835 |
| C | 4.643176 | -0.264524 | -1.145599 | O | -4.486737 | 1.572868 | 0.144128 | H | 6.64455 | 0.195938 | -0.409349 |
| C | 4.095699 | 0.942052 | -0.935517 | H | -0.65061 | 0.238586 | 1.56046 | H | 5.961117 | -1.326985 | 0.200008 |
| C | -0.357305 | -0.767801 | -0.289904 | C | -0.110256 | 1.957362 | -1.312747 | H | 1.37627 | 4.584276 | -0.132089 |
| C | -1.038782 | 0.343996 | 0.530994 | C | -2.493409 | -1.76864 | 2.20402 | H | 3.123889 | 4.840194 | 0.09813 |
| C | -0.794961 | 1.81259 | 0.049238 | O | 1.110518 | -1.768957 | 1.422338 | H | 2.545331 | 3.786111 | -1.198917 |
| C | -0.065453 | 2.667084 | 1.114324 | H | 1.729918 | -0.180828 | 0.16996 | H | -4.323744 | -2.767071 | 0.400025 |
| C | 1.377974 | 2.299635 | 1.314388 | H | 3.515161 | -1.546907 | 0.764924 | H | -2.814104 | -2.435805 | -0.504286 |
| C | 2.443954 | 2.910936 | 0.772733 | H | 4.823903 | -3.048002 | -0.874166 | H | 3.103843 | -3.46087 | 1.974385 |
| C | 3.847957 | 2.441994 | 1.076088 | H | 3.285478 | -3.438854 | -1.628244 | H | -7.354983 | -0.168291 | -1.825493 |
| C | 4.679566 | 2.096148 | -0.174169 | H | 4.370726 | -1.572529 | -2.823092 | H | -5.990197 | -0.652234 | -2.906431 |
| C | 0.914048 | -3.602524 | -0.263259 | H | 2.860743 | -0.999628 | -2.100047 | H | -5.841228 | 0.796417 | -1.885501 |
| C | 6.019613 | -0.662702 | -0.678029 | H | 3.083297 | 1.10725 | -1.326211 | H | -4.571499 | 2.51778 | -0.06689 |
| C | 2.355883 | 4.093103 | -0.156876 | H | -0.349521 | -0.525802 | -1.362032 | H | -0.674374 | 1.443987 | -2.104315 |
| C | -2.202978 | 2.374569 | -0.10621 | H | -0.931147 | -1.693897 | -0.185095 | H | -0.065478 | 3.024372 | -1.573819 |
| C | -3.169942 | 1.317639 | 0.207415 | H | -0.174807 | 3.719256 | 0.814291 | H | 0.913603 | 1.55753 | -1.289817 |
| C | -2.548191 | 0.192947 | 0.602469 | H | -0.607759 | 2.551789 | 2.067553 | H | -2.330672 | -1.11457 | 3.07205 |
| O | -2.512775 | 3.504976 | -0.409729 | H | 1.570368 | 1.437194 | 1.964366 | H | -3.062104 | -2.644061 | 2.548526 |
| C | -3.269444 | -1.024448 | 1.115636 | H | 3.810615 | 1.565156 | 1.741203 | H | -1.503345 | -2.108493 | 1.863881 |
| C | -3.683321 | -1.975726 | -0.015868 | H | 4.370056 | 3.245328 | 1.62516 |  |  |  |  |
| O | -4.379412 | -1.288963 | -1.044983 | H | 5.708906 | 1.880209 | 0.139771 |  |  |  |  |
|  |  |  |  |  |  |  |  |  |  |  |  |

**Table S7** Inhibitory effect of compounds on four strains of bacteria.

| **Compound** | **Density (µM)** | **Inhibition rate (%)** | | | |
| --- | --- | --- | --- | --- | --- |
|  |  | ***E. coli*** | ***S. aureus subsp. aureus*** | ***S. enterica subsp. enterica*** | ***P. aeruginosa*** |
| Penicillin G Sodium | 7 |  | 99.725±0.16 |  |  |
|  | 14 |  |  | 100.128±0.074 |  |
| Ceftazidime | 4 | 99.506±0 |  |  | 99.945±0.079 |
| **4** | 100 | 12.755±0.7 | -38.73±0.78 | 30.607±3.602 | 4.175±1.578 |
| **5** | 100 | 2.92±2.331 | 31.232±0.78 | 25.323±1.711 | -7.707±1.657 |
| **10** | 100 | 14.513±0.078 | -40.108±3.896 | 24.495±0.721 | -4.584±2.761 |
| **11** | 100 | 13.304±0.7 | 30.452±2.922 | 20.23±2.972 | -4.249±0.553 |

**Table S8** Inhibitory effect of compounds on five strains of Cytotoxicity.

| **Compound** | **Density (µM)** | **HL-60** | | **A549** | | **SMMC-7721** | | **MDA-MB-231** | | **SW480** | |
| --- | --- | --- | --- | --- | --- | --- | --- | --- | --- | --- | --- |
|  |  | **Average** | **SD** | **Average** | **SD** | **Average** | **SD** | **Average** | **SD** | **Average** | **SD** |

| **4** | 40 | -39.66 | 0.40 | 8.88 | 1.73 | 11.67 | 1.60 | 7.48 | 0.36 | 6.00 | 4.68 |
| --- | --- | --- | --- | --- | --- | --- | --- | --- | --- | --- | --- |
| **5** | 40 | 0.00 | 2.90 | 3.94 | 0.61 | 13.79 | 2.76 | 15.22 | 0.65 | 4.51 | 1.27 |
| **10** | 40 | -19.95 | 3.29 | 10.09 | 0.52 | 37.59 | 2.00 | 1.49 | 2.61 | 6.70 | 1.17 |
| **11** | 40 | -28.00 | 2.95 | 17.28 | 2.00 | 46.84 | 0.46 | 1.93 | 1.56 | 11.57 | 3.14 |
